# Artificial Photosynthases: Single-Chain Nanoparticles with Manifold Visible-Light Photocatalytic Activity for Challenging “in Water” Organic Reactions

**DOI:** 10.1021/jacs.4c02718

**Published:** 2024-04-19

**Authors:** Davide Arena, Ester Verde-Sesto, Iván Rivilla, José A. Pomposo

**Affiliations:** † Centro de Física de Materiales (CSIC-UPV/EHU)-Materials Physics Center MPC, P° Manuel Lardizabal 5, E-20018 Donostia, Spain; ‡ IKERBASQUE-Basque Foundation for Science, Plaza Euskadi 5, E-48009 Bilbao, Spain; § Departamento de Química Orgánica I, Centro de Innovación en Química Avanzada (ORFEO−CINQA), University of the Basque Country (UPV/EHU), Faculty of Chemistry, P° Manuel Lardizabal 3, E-20018 Donostia, Spain; ∥ Donostia International Physics Center (DIPC), P° Manuel Lardizabal 4, E-20018 Donostia, Spain; ⊥ Departamento de Polímeros y Materiales Avanzados: Física, Química y Tecnología, University of the Basque Country (UPV/EHU), Faculty of Chemistry, P° Manuel Lardizabal 3, E-20018 Donostia, Spain

## Abstract

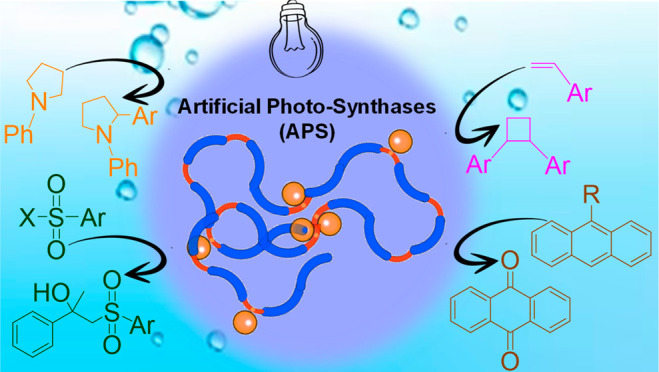

Photocatalyzed reactions of organic substances in aqueous media are challenging transformations, often because of scarce solubility of substrates and catalyst deactivation. Herein, we report single-chain nanoparticles, SCNPs, capable of efficiently catalyzing four different “in water” organic reactions by employing visible light as the only external energy source. Specifically, we decorated a high-molecular-weight copolymer, poly(OEGMA_300_-*r*-AEMA), with iridium(III) cyclometalated complex pendants at varying content amounts. The isolated functionalized copolymers demonstrated self-assembly into noncovalent, amphiphilic SCNPs in water, which enabled efficient visible-light photocatalysis of two reactions unprecedentedly reported in water, namely, [2 + 2] photocycloaddition of vinyl arenes and α-arylation of *N*-arylamines. Additionally, aerobic oxidation of 9-substituted anthracenes and β-sulfonylation of α-methylstyrene were successfully carried out in aqueous media. Hence, by merging metal-mediated photocatalysis and SCNPs for the fabrication of artificial photoenzyme-like nano-objects—i.e., artificial photosynthases (APS)—our work broadens the possibilities for performing challenging “in water” organic transformations via visible-light photocatalysis.

In nature, only three types of enzymes carry out purely photocatalytic organic reactions.^1−3^ Indeed, the design of abiotic protein nanoreactors capable of custom photoreactions requires extensive genetic engineering effort.^4−6^ In organic photochemistry, the scarce solubility of reactants in aqueous media and severe catalyst deactivation are of central concern for the replacement of organic solvents with water. While several strategies have been recently proposed,^7−10^ only a few works have been devoted to the use of ultrafine soft nano-objects as efficient visible-light photocatalysts of “in water” organic reactions.^11,12^

Single-chain nanoparticles (SCNPs)—as intramolecularly self-folded synthetic polymer chains with ultrasmall size (2–20 nm)—are posed as perfect candidates for advanced, next-generation enzyme-mimetic catalyst preparation.^13−16^ Despite the extensive use of SCNPs as nanoreactors for a plethora of organic reactions,^13−19^ only a few works have disclosed the use of SCNPs for photocatalytic applications.^12,20^ Herein, we report the construction of unimolecular soft nano-objects endowed with broad, manifold photocatalytic activity in water and constructed by taking advantage of the protein-mimetic architecture of polymeric SCNPs (see Scheme 1). These artificial photosynthases (APS) are used to perform a collection of four visible-light-induced transformations using water as the sole medium.

**Scheme 1:**
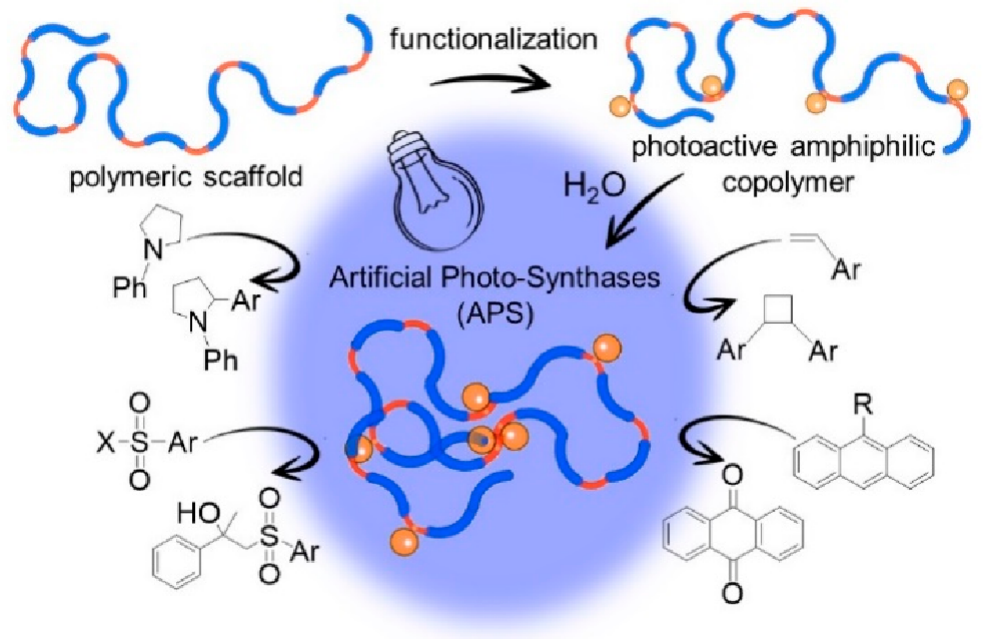
Illustration of the Preparation of an Artificial Photosynthase (APS) Endowed with Manifold Photocatalytic Activity toward “in Water” Reactions

As APS precursor, we prepared the polymeric scaffold poly(OEGMA_300_-*r*-AEMA) **P1** of high molecular weight (>150 kDa), low dispersity (1.06), and controlled chemical composition (Table 1) via reversible addition–fragmentatation chain transfer (RAFT) copolymerization of the monomers 4-acetoacetoxyethyl methacrylate (AEMA) and (oligoethylene glycol monomethyl ether) methacrylate (OEGMA_300_). To endow **P**_**1**_ with photocatalytic activity, we exploited the β-ketoester reactivity provided by the hydrophobic AEMA groups. We prepared iridium (Ir)(III)-containing copolymers at three loading (L^Ir^) regimes, **P**_**1**_**–Ir**_**10**_, **P**_**1**_**–Ir**_**23**_, and **P**_**1**_**–Ir**_**40**_ (L^Ir^ = 10, 23, and 40 mol % with respect to AEMA units, respectively), by decorating the copolymer **P**_**1**_ through reaction with the dihydroxotetrakis[2-(2-pyridinyl)phenyl]diiridium(III) dimer [Ir(ppy)_2_OH]_2_**C**_**1**_ under mild conditions [see the Supporting Information (SI)].^21^

**Table 1:**
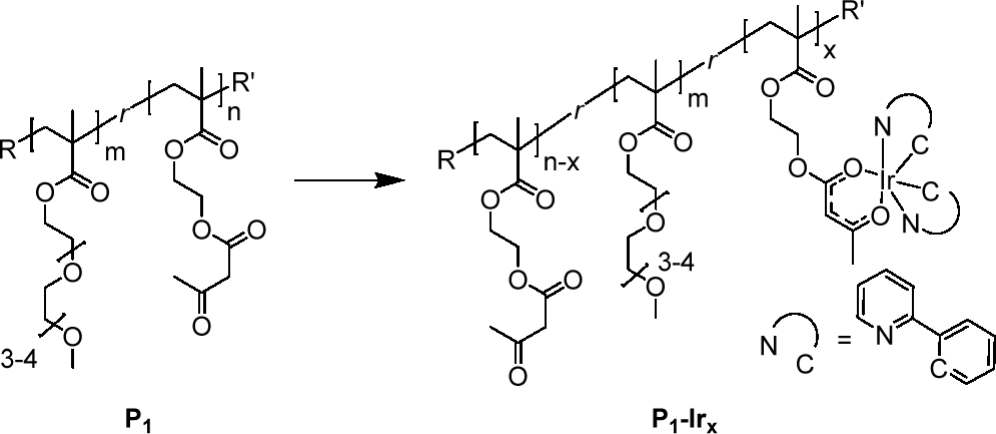
Properties of Neat (**P_1_**) and Functionalized Copolymers (**P_1_−Ir_*x*_**) Synthesized in This Work

sample	*M*_w_ (kDa)^a^	*Đ*^b^	AEMA (mol %)^c^	L^Ir^ (mol %)^d^
**P**_**1**_	169.8	1.06	20	
**P**_**1**_**–Ir**_**10**_	178.6	1.10	18	10
**P**_**1**_**–Ir**_**23**_	174.5	1.13	15.4	23
**P**_**1**_**–Ir**_**40**_	211.7	1.03	12	40

a Weight-average molecular weight.

b Dispersity.

c Molar content of AEMA units.

d Iridium content (see the SI).

Thanks to their amphiphilic nature, high molecular weight (>150 kDa), and finely tuned composition, stable, self-assembled SCNPs^22,23^ that we denoted as **SCNP-P**_**1**_, **APS-Ir**_**10**_, **APS-Ir**_**23**_, and **APS-Ir**_**40**_ were obtained upon simple dissolution of **P**_**1**_, **P**_**1**_**–Ir**_**10**_**, P**_**1**_**–Ir**_**23**_, and **P**_**1**_**–Ir**_**40**_, respectively, in water. Self-assembly was ascertained by measuring via dynamic light scattering (DLS) the difference Δ*D*_h_ between hydrodynamic diameters *D*_h_ of **P**_**1**_, **P**_**1**_**–Ir**_**10**_**, P**_**1**_**–Ir**_**23**_, and **P**_**1**_**–Ir**_**40**_ in tetrahydrofuran, THF, (*good* solvent for AEMA and OEGMA_300_) and those of **SCNP-P**_**1**_, **APS-Ir**_**10**_, **APS-Ir**_**23**_, and **APS-Ir**_**40**_ measured in water (*selective* solvent for OEGMA_300_). Iridium-functionalized copolymers all presented a positive Δ*D*_h_ (Table 2) because of the formation of a self-collapsed architecture (see Figure 1 and the SI).

**Table 2:** Hydrodynamic Radii Measured in THF (Good Solvent) and Water (Selective Solvent)

sample	*D*_h_(THF) (nm)	sample	*D*_h_(water) (nm)
**P**_**1**_	9.9	**SCNP-P**_**1**_	8.7
**P**_**1**_**–Ir**_**10**_	12.3	**APS-Ir**_**10**_	10.4
**P**_**1**_**–Ir**_**23**_	11.8	**APS-Ir**_**23**_	9.6
**P**_**1**_**–Ir**_**40**_	10.4	**APS-Ir**_**40**_	9.1

**Figure 1:**
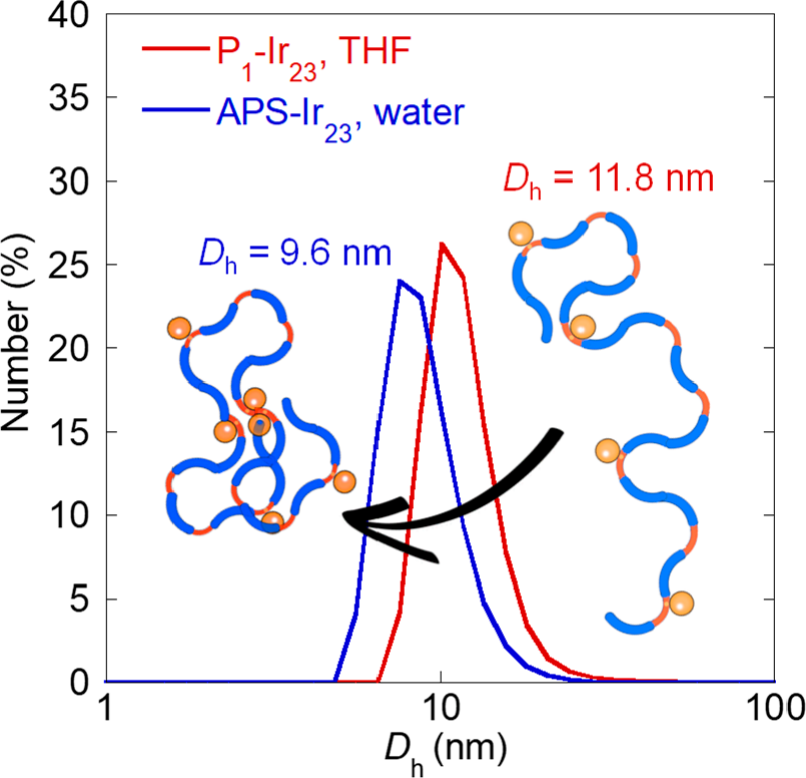
Illustration of the reduction in hydrodynamic size due to self-assembly of P_1_–Ir_23_ (linear architecture) to APS–Ir_23_ (SCNP architecture) upon change from THF (good solvent) to water (selective solvent).

Additional evidence of the formation of a self-assembled conformation in **APS-Ir**_**10**_, **APS-Ir**_**23**_, and **APS-Ir**_**40**_ was obtained through photoluminescence (PL) experiments. As illustrated in Figure 2a, we measured the PL of eight solutions of a model compound, bis(2-phenylpyridine)(methyl acetoacetonate)iridium(III) **C**_**2**_ (see the SI), in THF/water mixtures upon increase of the water content (from 0% to 70%). We observed a decrease in the PL intensity of **C**_**2**_ at high water content, which is consistent with literature data reported for the analogous complex bis(2-phenylpyridine)(acetylacetonate)iridium(III), Ir(ppy)_2_(acac).^24,25^ Conversely, **APS-Ir**_**10**_, **APS-Ir**_**23**_, and **APS-Ir**_**40**_ in water displayed significant PL intensity enhancement with respect to **P**_**1**_**–Ir**_**10**_**, P**_**1**_**–Ir**_**23**_, and **P**_**1**_**–Ir**_**40**_ recorded in two different nonselective solvents (chloroform and THF) (see Figure 2b). This finding suggests that confinement of the hydrophobic photocatalyst in the limited space of the self-assembled **APS-Ir**_**10**_, **APS-Ir**_**23**_, and **APS-Ir**_**40**_ induces significant aggregation-enhanced emission (AEE).

**Figure 2:**
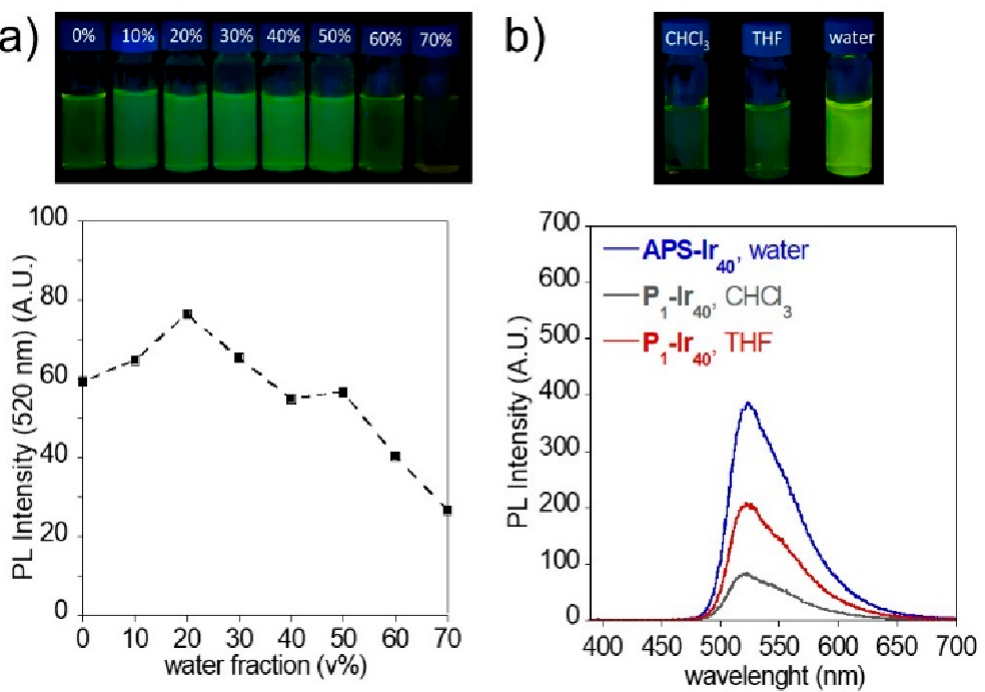
(a) PL emission of model compound bis(2-phenylpyridine)(methyl acetoacetonate)iridium(III) **C**_**2**_ in THF/water mixtures (from 0% to 70%) ([Ir(III)] = 3 μM, λ_exc_ = 450 nm). (b) Illustration of the aggregation-enhanced emission (AEE) of **APS-Ir_40_** in water vs **P_1_–Ir_40_** in CHCl_3_ and THF under LED illumination ([Ir(III)] = 3 μM, λ_exc_ = 450 nm, λ_em_ (max) = 521 nm).

With the self-assembled SCNPs in hand, we tested their suitability as artificial photosynthases (APS) for in water photocatalysis of a variety of organic transformations, which were selected from among the plethora of iridium(III) cyclometalated complex-mediated reactions in organic solvents.^26−28^

Visible light has been recently applied successfully as an energy source for [2 + 2] cycloadditions in organic solvents;^29^ herein, we report an unprecedented procedure that employs water as the sole reaction medium. For instance, an **APS-Ir**_**40**_ solution was prepared by dissolving 2 mg of **P**_**1**_**–Ir**_**40**_ in 1 mL of deionized water, which was then charged with 58 μmol of the vinylic compound **1a**, and the resulting mixture was left stirring at room temperature and under LED illumination (λ_max_ = 450 nm) for 12 h. After this time, the extracted crude product was analyzed via quantitative ^1^H NMR (see the SI) for conversion (*c*%) determination. Multiplets at 3.54 and 3.98 ppm (see Figure 3) indicate the formation of the 1,2-bis-substituted cyclobutane product **2a**, which was then isolated in 90% yield as a mixture of cis and trans diastereomers. To our delight, we observed similar results when exploring a variety of substrates, as illustrated in Table 3.

**Figure 3:**
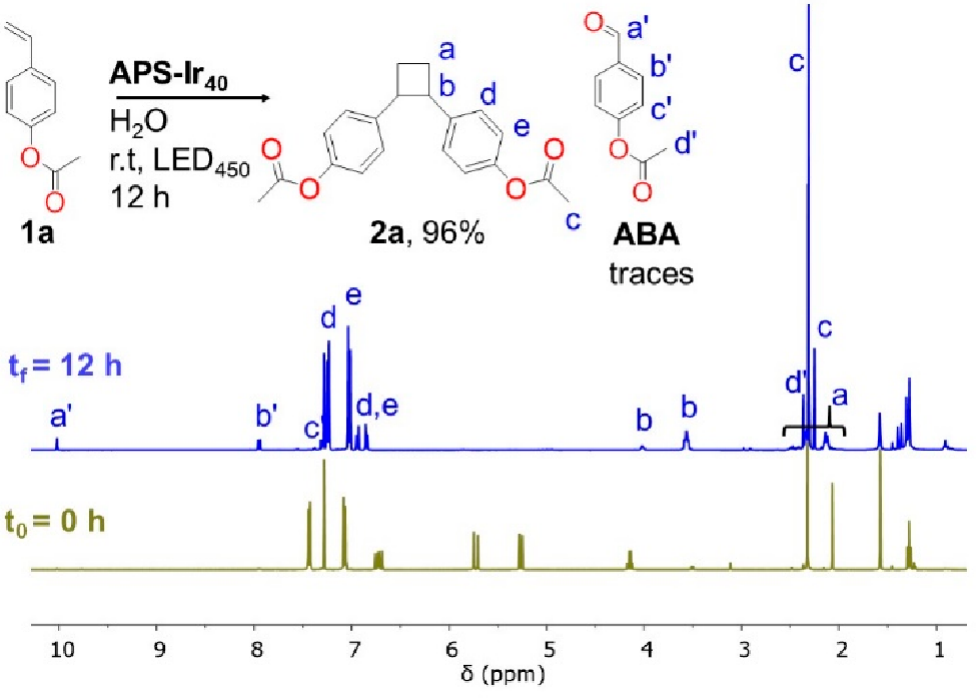
^1^H NMR of the crude of “in water” [2 + 2] photocycloaddition reaction of **1a** in the presence of **APS-Ir_40_** (see the text).

**Table 3:**
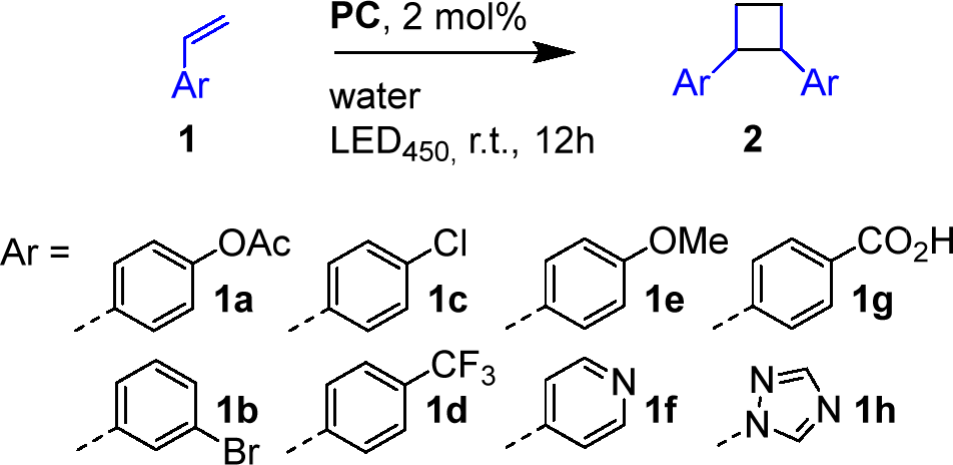
Photocatalyzed “in Water” [2 + 2] Cycloaddition of Vinyl Arenes (CA Reaction)^a^

entry	photocatalyst (PC)	transformation	*c*%^b^	trans/cis^c^
1	**APS-Ir**_**40**_	**1a → 2a**	96(90)^d^	1:0.3
2	**APS-Ir**_**23**_	**1a → 2a**	94	1:0.3
3	**APS-Ir**_**10**_	**1a → 2a**	96	1:0.3
4	**APS-Ir**_**40**_	**1b → 2b**	97	1:0.2
5	**APS-Ir**_**40**_	**1c → 2c**	96	1:0.3
6	**APS-Ir**_**40**_	**1d → 2d**	90	1:0.3
7	**APS-Ir**_**40**_	**1e → 2e**	60	1:0.3
8	**APS-Ir**_**40**_	**1f → 2f**	79	1:0.3
9	**APS-Ir**_**40**_	**1g → 2g**	n.p.^e^	
10	**APS-Ir**_**40**_	**1h → 2h**	n.p^e^	
11	no PC	**1a → 2a**	n.p.^e^	
12	**C_2_**	**1a → 2a**	63	1:0.3

a See SI for experimental details.

b Conversion from ^1^H NMR.

c Determined by ^1^H NMR.

d Isolated yield in parentheses.

e No product.

Considering typical redox potentials of vinyl arenes (e.g., *E*_ox_ = 1.97 V vs SCE, *E*_red_ = −2.53 V vs SCE)^30,31^ and photocatalyst excited-state potentials (e.g., E*_ox_ = 0.43 V vs SCE and E*_red_ = −2.57 V vs SCE),^26^ it seems difficult to imagine the activation of olefins **1a**–**1f** by typical single-electron transfer (SET).^32^ We hypothesized that, helped by the locally hydrophobic packed environment of the APS, these unlike processes could be allowed via an energy transfer (*E*_T_) mechanism because of the similar triplet energies of the involved species (e.g., *E*_T_ = ∼60 kcal mol^–1^ for vinyl arenes, E_T_ = ∼55 kcal mol^–1^ for **C**_**2**_).^33^

Byproducts formed in trace amounts, among which we assigned^34^ the structure of 4-acetoxybenzaldehyde, **ABA**, to be the major constituent in agreement with the reactivity of electron-rich vinyl arenes with reactive oxygen species that may be generated during irradiation.^35^ Performance of the same reaction using the model photocatalyst **C**_**2**_ dropped conversion to 63% (see the SI) with the remaining components of the crude being the reactant and byproducts. No conversion was observed by employing water-soluble substrates (**1g**, **1h**, Table 3), probably because of differences in the *E*_T_ values.

Interestingly, when 9-substituted anthracenes were employed as the substrate, we observed the formation of anthraquinone **4**. Since singlet oxygen and other reactive oxygen species (ROS) are generated upon excitation of **C**_**2**_ in organic solvents under aerobic conditions,^36^ we surmised that **APS-Ir**_**10**_, **APS-Ir**_**23**_, and **APS-Ir**_**40**_ could be efficient photocatalysts for the “in water” oxidation of 9-substituted anthracenes **3** to anthraquinone **4** via reaction with ROS species^37^ (see Table 4). The formation of **4** that was confirmed by ^1^H NMR, which is consistent with literature data,^38^ resulted in being completely neglected when the complex is not confined within the SCNP (see the SI).

**Table 4:**
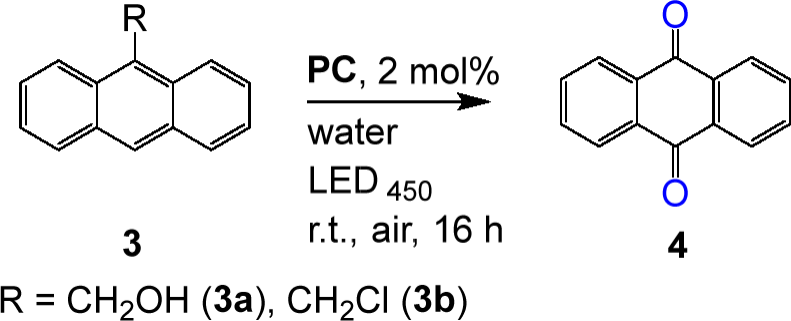
Photocatalyzed “in Water” Oxidation of 9-Substituted Anthracenes (OA Reaction)^a^

entry	photocatalyst (PC)	substrate	*c*%^b^
1	**APS-Ir**_**40**_	**3a**	62(58)^c^
2	**APS-Ir**_**23**_	**3a**	21
3	**APS-Ir**_**10**_	**3a**	19
4	**APS-Ir**_**40**_	**3b**	59
5	no PC	**3a**	n.p.^d^
6	**C_2_**	**3a**	n.p.^d^

a See SI for experimental details.

b Conversion from ^1^H NMR.

c Isolated yield in parentheses.

d No product.

Next, we evaluated the capability of **APS-Ir**_**10**_, **APS-Ir**_**23**_, and **APS-Ir**_**40**_ to catalyze visible-light-induced α-arylation of arylamines that, since its discovery reported by McMillan et al.,^39,40^ have never been reported in water. Table 5, entries 1–3 show the procedure in organic solvents. To enable the photocatalytic cycle to occur in water, we prepared **APS-Ir**_**40**_ by dissolving 2 mg of **P1–Ir40** in 1 mL of degassed deionized water. We then charged the APS aqueous solution with 29 μmol of **6** and with a large excess of sodium acetate, NaOAc, (84 equiv). The resulting mixture was deoxygenated by three consecutive vacuum/argon backfill cycles, finally 87 μmol of **5** were added and left stirring at room temperature and under blue LED (λ_max_ = 450 nm) irradiation for 12 h. Under these conditions, product **7** was obtained by using **APS-Ir**_**40**_ as photocatalyst in 57% conversion and 48% purified yield (Table 5, entry 5). Conversion was found to decrease upon reduction of the iridium loading in the APS photocatalyst (Table 5, entries 6 and 7). We attributed the striking conversion drop upon either lowering or increasing the base concentration (Table 5, entries 4 and 10) to the altered acidities of the reactants and additives in aqueous media with respect to the reaction conditions reported in literature for organic solvent.^39,41^

**Table 5:**
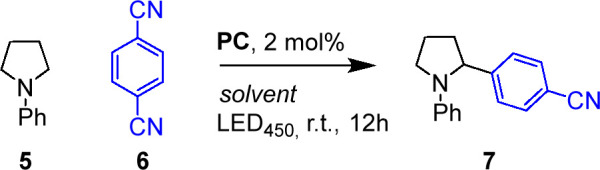
Photocatalyzed “in Water” α-Arylation of Arylamines (AA Reaction)^a^

entry	PC	mol % PC	[NaOAc] M^b^	solvent^b^	*c*%^c^
1	**C_2_**	0.5	0.06	DMA	>95(95)^d^
2	**P**_**1**_**–Ir**_**40**_	0.5	0.06	DMA	>95
3	**C_2_**	0.5	0.06	MeOH	8
4	**APS-Ir**_**40**_	2	0.06	H_2_O	n.p.^e^
5	**APS-Ir**_**40**_	2	2.4	H_2_O	57(48)^d^
6	**APS-Ir**_**23**_	2	2.4	H_2_O	22
7	**APS-Ir**_**10**_	2	2.4	H_2_O	21
8	**C_2_**	2	2.4	H_2_O	n.p.^e^
9	**APS-Ir**_**40**_	2	2.4	H_2_O+DMA	33
10	**APS-Ir**_**40**_	2	sat.^f^	H_2_O	24
11	no PC		2.4	H_2_O	n.p.^e^

a See the SI for experimental details.

b NaOAc = sodium acetate; DMA = dimethylacetamide; MeOH = methanol.

c Conversion of **7** from ^1^H NMR.

d Isolated yield of **7** in parentheses.

e No product.

f Saturated in NaOAc.

Inspired by pioneering work by Lipshutz et al.,^11^ we tested **APS-Ir**_**10**_, **APS-Ir**_**23**_, and **APS-Ir**_**40**_ as photocatalysts for the “in water” β-hydroxysulfonylation of α-methylstyrene **8**. We carried out the photocatalytic reactions under oxygen-free conditions by charging the readily prepared APS aqueous solutions with **8** and sulfonyl halides **9** (see the SI). Despite the intrinsic water-sensitive nature of these reactants, we could observe up to 67% conversion into desired product **10** (58% isolated yield) (Table 6, entry 2). As expected, a significant reduction was observed with the model compound **C**_**2**_ as a photocatalyst (Table 6, entry 6). For this reaction, no significant effect of iridium loading in the APS photocatalyst on conversion is observed (Table 6, entries 1–3).

**Table 6:**
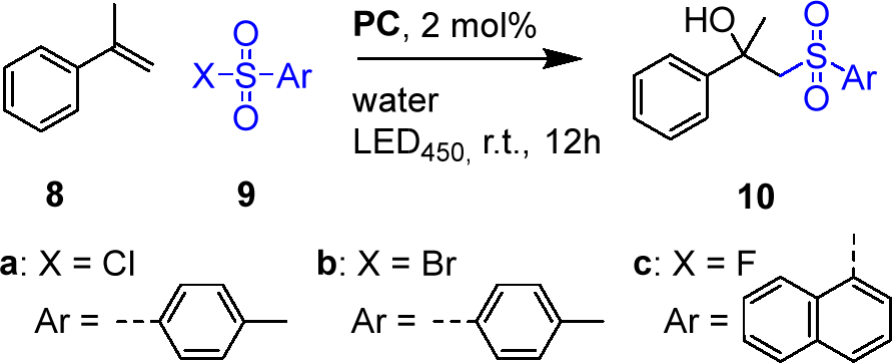
Photocatalyzed “in Water” β-Hydroxysulfonylation of α-Methylstyrene (HS Reaction)^a^

entry	PC	transformation	*c*%^b^
1	**APS-Ir**_**40**_	**9a → 10a**	50
2	**APS-Ir**_**23**_	**9a → 10a**	67(58)^d^
3	**APS-Ir**_**10**_	**9a → 10a**	52
4	**APS-Ir**_**23**_	**9b → 10b**	n.p.^e^
5	**APS-Ir**_**23**_	**9c → 10c**	21
6	C_2_	**9a → 10a**	23
7	no PC	**9a → 10a**	n.p.^e^

a See the SI for experimental details.

b Conversion from ^1^H NMR.

d Isolated yield in parentheses.

e No product.

In summary, artificial photosynthases (APS) arise by endowing enzyme-mimetic single-chain nanoparticles, SCNPs, with broad visible-light photocatalytic activity for challenging “in water” organic reactions. We introduce a first generation of APS resulting from the decoration of an amphiphilic high-molecular-weight copolymer, poly(OEGMA_300_-*r*-AEMA), with iridium(III) cyclometalated complex pendants followed by its own self-assembly in water. APS enabled efficient visible-light photocatalysis of a variety of organic transformations in an aqueous solution at room temperature and under LED illumination (λ_max_ = 450 nm). This work broadens the possibilities for performing challenging “in water” organic transformations via APS-mediated visible-light photocatalysis.

## Supporting Information Available

Materials and methods, synthesis and characterization of compounds, effect of APS type on conversion, kinetic assays of APS, recyclability of APS, SEC chromatograms, DLS data, UV–vis spectra, NMR spectra and supplementary references (PDF)
