# Supplementary material for: Artificial Photosynthases: Single-Chain Nanoparticles with Manifold Visible-Light Photocatalytic Activity for Challenging “in Water” Organic Reactions
Source: J Am Chem Soc. 2024 Apr 19;146(21):14397–403. doi: 10.1021/jacs.4c02718 (PMC11140743; doi:10.1021/jacs.4c02718)
Supplement: Supplementary file 1 — ja4c02718_si_001.pdf [file ja4c02718_si_001.pdf]

Supporting Information for:

Artificial Photo-Synthases: SCNPs with Manifold Visible-Light Photocatalytic Activity for Challenging "*in Water*" Organic Reactions

Davide Arena,[1]* Ester Verde-Sesto,[1,2] Iván Rivilla,[2,3,4] and José A. Pomposo[1,2,5]*

[1]Centro de Física de Materiales (CSIC-UPV/EHU)-Materials Physics Center MPC, P° Manuel Lardizabal 5, E-20018 Donostia, Spain

[2]IKERBASQUE-Basque Foundation for Science, Plaza Euskadi 5, E-48009 Bilbao, Spain

[3]Departamento de Química Orgánica I, Centro de Innovación en Química Avanzada (ORFEO−CINQA), University of the Basque Country (UPV/EHU), Faculty of Chemistry, P° Manuel Lardizabal 3, E-20018 Donostia, Spain

[4]Donostia International Physics Center (DIPC), P° Manuel Lardizabal 4, E-20018 Donostia, Spain

[5]Departamento de Polímeros y Materiales Avanzados: Física, Química y Tecnología. University of the Basque Country (UPV/EHU), Faculty of Chemistry, P° Manuel Lardizabal 3, E-20018 Donostia, Spain

*E-mail: darena001@ikasle.ehu.eus, josetxo.pomposo@ehu.eus

Table of Contents

1. Materials and Methods S2
   1.1. Solvents and Reagents S2
   1.2. Analytical Methods and Techniques S2
2. Synthesis and Characterization of Compounds S6
   2.1. Synthesis of Artificial Photo-Synthases (APS) S6
   2.2. *"In Water"* [2+2] Photocycloaddition of Vinyl Arenes S9
   2.3. *"In Water"* Oxidation of 9-Substituted Anthracenes S11
   2.4. *"In Water"* α-Arylation of Arylamines S12
   2.5. *"In Water"* β-Hydroxysulfonylation of α-Methyl Styrene S14
3. Effect of APS type on Conversion S16
4. Kinetic Assays of APS S17
5. Recyclability of APS S21
6. SEC Chromatograms S22
7. DLS Data S24
8. UV-Vis Spectra S25
9. NMR Spectra S28
10. Supplementary References S61

1. Materials and Methods

1.1. Solvents and Reagents

Unless otherwise noted, all reagents and solvents were used as received from vendors. (Oligoethylene glycol monomethyl ether) methacrylate ($OEGMA_{300}$, average molecular weight = 300 Da) (>99%) and 4-acetoacetoxyethyl methacrylate (AEMA) (>95%) were purchased from TCI Europe N.V. and were filtered over basic alumina before use. *n*-Hexane (96%), acetone (>99%), methylene chloride (CH_2Cl_2) (>99%, +0.2 % EtOH), *n*-pentane (99%), ethyl acetate (AcOEt) (>99.8) and diethyl ether (Et_2O) (>99%, +7 ppm BHT) were purchased from Scharlab. Tetrahydrofuran (THF) (>99 %, +0.025% BHT) and methanol (MeOH) (>99%) were purchased from Fisher Scientific, inhibitor-free THF was obtained by filtration over basic alumina. 1,4-Dioxane (>99%), methyl acetoacetate (99%), 2-ethoxyethanol (≥99%), sodium hydroxide (NaOH) (≥98%), chloroform (>99%, +100-200 ppm amylene), α-methyl styrene (99%), 2-naphtalenesulfonyl fluoride (95%), sodium acetate (NaOAc) (>99%), anhydrous magnesium sulfate ($MgSO_4$) (>99%), sodium chloride (NaCl) (>99%), silver(I) trifluoromethansulfonate (AgOTf) (≥98%), 4-cyano-4-(thiobenzoylthio)pentanoic acid (CPADB), 1,4-dicyanobenzene (DCB) (98%), triethylamine (TEA) (>99%), linalool (97%) and dimethylacetamide (DMA) (≥99%) were purchased from Sigma-Aldrich. *p*-Toluenesulfonyl chloride (99%) and *p*-toluenesulfonyl bromide (98%) were purchased from Sigma-Aldrich and handled under argon atmosphere. 4-Acetoxystyrene (96%), 4-chlorostyrene (99%), 3-bromostyrene (97%), 4-trifluoromethylstyrene (99%), 4-vinylpyridine (≥95%) and 1-vinyl-1,2,4-triazole (≥97%) were purchased from Sigma-Aldrich and used after filtering over basic alumina. Bis(μ-chloro)tetrakis(2-phenyl-pyridinato)diiridium(III) (98%), iridium(III) chloride trihydrate (98%), 2-phenylpyridine (>99%), 4-carboxystyrene (>99%) and 4-methoxystyrene (>99%) were purchased from BLD Pharma. Azobisisobutyronitrile (AIBN) (98%) was purchased from Fluka and recrystallized from MeOH prior use. Silica gel for column chromatography (0.035-0.07 nm 60 A) was purchased from Acros Organics. Basic alumina (0.063-0.2 mm) was purchased from Merck. *N*-phenylpyrrolidine (>98%) was purchased from Alpha Aesar. Deuterated chloroform ($CDCl_3$, 99.8% D, + 0.03% tretramethylsilane) for NMR analysis was purchased from Eurisotop. Deionized water was obtained from a Thermoscientific Barnstead TII System.

1.2. Analytical Methods and Techniques

Nuclear Magnetic Resonance (NMR) Spectroscopy: ^1H and ^{13}C NMR spectra were recorded at room temperature (r.t.) on a Bruker spectrometer operating at 400 MHz, using $CDCl_3$ as solvent.

- Hydrophobic monomer content in the copolymer, %AEMA (mol%), was calculated according to Equation S1:

$$\%AEMA\ (mol\%) = \frac{S_{AEMA}^{OMe}}{S_{OEGMA}^{OMe} + S_{AEMA}^{OMe}} \times 100 \qquad \text{Eq.S1}$$

where $S^{OMe}{}_{AEMA}$ is the integrated area of the signal corresponding to the methoxylic protons of the AEMA units within the copolymer (2.29 ppm) and $S^{OMe}{}_{OEGMA}$ is the integrated area of the signal corresponding to the methoxylic protons of OEGMA units (3.37 ppm).

- NMR conversion of the photo [2+2] cycloaddition reaction, *c*% (mol%), was calculated according to Equation S2:

$$c\%\ (mol\%) = \frac{\Sigma^P}{S^R + \Sigma^P} \times 100 \qquad \text{Eq.S2}$$

where Σ^P is the sum of the normalized integrated area signals of the alkylic protons of all the isomers of the product (3.56 ppm and 4.02 ppm) and S^R is the normalized area of the signal of the vinylic proton of the reactant (5.74-5.69 ppm). The diastereomeric ratio *d.r.* was calculated using the integrated arylic proton signals of the product (7.03-7.01 ppm for the *trans* isomer and 6.86-6.83 ppm for the *cis* isomer).

- NMR conversion of the α-arylation of arylamines, *c*% (mol%), was calculated according to Equation S3:

$$c\%\ (mol\%) = \frac{S^P}{S_{DCB}^{Ar} + S^P} \times 100 \qquad \text{Eq.S3}$$

or Equation S4 when an internal standard was used:

$$c\%\ (mol\%) = \frac{S^P n^{SI}}{S^{SI} + n_0^P} \times 100 \qquad \text{Eq.S4}$$

where S^P is the normalized area of the arylic signals of the product (7.60-7.58 ppm), $S^{Ar}{}_{DCB}$ is the normalized area of the aromatic signal of the reactant 1,4-dicyanobenzene (7.79 ppm), n^{SI} is the internal standard (linalool) number of moles corresponding to the weighted amount in the tube, S^{SI} is the normalized intensity of the signal of the internal standard (5.95-5.89 ppm) and $n^P{}_0$ is the number of moles of the reactant 1,4-dicyanobenzene corresponding to the weighted amount before the reaction.

- NMR conversion of the oxidation of 9-substituted anthracenes, $c\%$ (mol%), was calculated according to Equation S5:

$$c\%\ (mol\%) = \frac{S^P}{S^R + S^P} \times 100 \qquad \text{Eq.S5}$$

where S^P is the normalized area of the arylic signals of the anthraquinone product (7.81-7.79 ppm), S^R is the normalized area of the signal of the reactant (*e.g,*. signal at 8.04-8.01 ppm for the 9-hydroxymethyl anthracene).

- NMR conversion of the β-hydroxysulfonylation reaction, $c\%$ (mol%), was calculated according to Equation S6:

$$c\%\ (mol\%) = \frac{S^P n^{SI}}{S^{SI} + n_0^P} \times 100 \qquad \text{Eq.S6}$$

where S^P is the normalized area of the arylic signals of the product (7.49-7.48 ppm), n^{SI} is the internal standard (1,4-dicyanobenzene) number of moles corresponding to the weighted amount in the tube, S^{SI} is the normalized intensity of the signal of the internal standard (7.79 ppm) and n^P_0 is the number of moles of the reactant α-methyl styrene corresponding to the amount before the reaction.

- The "*intra-particle photocatalytic unit density" **d*** is defined as in Equation S7:

$$\boldsymbol{d}\ (nm^{-3}) = 3\frac{n^{PC}}{4\pi r_h^3 n^{SCNP}} \qquad \text{Eq.S7}$$

where n^{PC} is the concentration (M) of the photocatalyst measured by UV-Vis spectrophotometry, n^{SCNP} is the concentration (M) of the nanoparticle in solution, which is derivable from the corresponding weighted amount and r_h is the hydrodynamic diameter measured by DLS.

Size-Exclusion Chromatography (SEC): SEC measurements were performed at 30 °C on an Agilent 1200 system equipped with PLgel 5μm Guard and PLgel 5μm MIXED-C columns, and triple detection: a differential refractive index (dRI) detector (Optilab Rex, Wyatt), a multi-angle laser light scattering (MALLS) detector (MiniDawn Treos, Wyatt), and a viscosimetric (VIS) detector (ViscoStar-II, Wyatt). Data analysis was performed with ASTRA Software (version 6.1) provided by Wyatt. THF was used as eluent at a flow rate of 1 mL min^{-1}. A value of dn/dc = 0.115 mLg^{-1} was used for copolymer **P1** and derivatives thereof.

Dynamic Light Scattering (DLS): DLS measurements were carried out at r.t. on a Malvern Zetasizer Nano ZS apparatus, using high precision quartz cells, light path 10×10 mm, provided by Hellma

Analytics. Data are given as by Number as an average of at least four measurements. All the solvents for sample preparation were filtered with 2 µM Teflon filters prior use.

UV-Vis Spectroscopy (UV-Vis): UV-Vis spectra were recorded at 25 °C in an Agilent 8453A apparatus with Peltier thermostatic cell holder, T-controller 89090A, using high precision quartz cells, light path 10×10 mm, provided by Hellma Analytics.

Photoluminescence (PL) Spectroscopy: PL spectra were recorded at r.t. on an Agilent Cary Eclipse spectrometer at an excitation wavelength of 365 nm, using high precision quartz cells, light path 10×10 mm, provided by Hellma Analytics. Samples were degassed by purging argon gas for 5 consecutive minutes before each measurement.

Photocatalytic Reactions, Irradiation Set-Up: Photoreactions were carried out using a Penn PhD Photoreactor, equipped with a 450 nm LED source, purchased from Merck. LED intensity was set at 100%, and a 4 mL vial-holder was used (10 cm light path to the reaction vessel and form the light source).

2. Synthesis and Characterization of Compounds

2.1. Synthesis of Artificial Photo-Synthases (APS)

*Synthesis of the Polymeric Precursor Poly(OEGMA$_{300}$-co-AEMA) (**P$_1$**)*

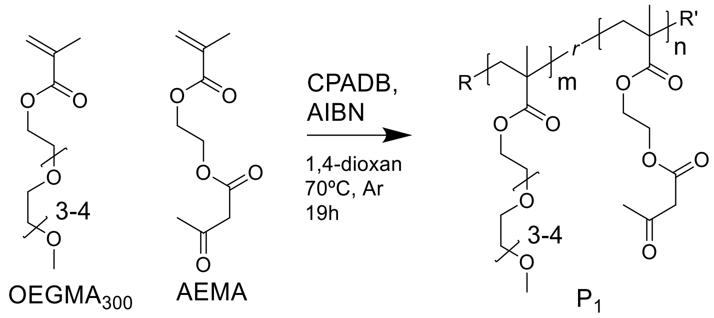

In an oven dried Schlenk flask equipped of a magnetic stir bar, 1.92 g (6.4 mmol) of OEGMA$_{300}$, 343 mg (1.6 mmol) of AEMA, 10.6 mg (38 µmol) of 4-cyano-4-(thiobenzoylthio)pentanoic acid (CPADB), 1.29 mg (7.7 µmol) of AIBN and 3.4 mL of 1,4-dioxane were added in this order. The flask was then sealed with a rubber septum and, after purging the solution with argon flow for 20 min., the reaction mixture was left stirring at 70ºC under argon atmosphere for 19 h. After this time, the reaction was quenched submerging the tube in liquid nitrogen. The crude was then precipitated in a large excess of *n*-hexane for three consecutive times, yielding the desired polymeric product **P$_1$**. Conversion: 75%, M_w (kDa) = 169.8, PDI = 1.06, %AEMA (mol%) = 20, average N = 566, ^{1}H NMR (400 MHz, $CDCl_3$): **δ** (ppm) = 4.33 (m., $CH_3COC\mathit{H_2}CO$), 4.14 (m., $CH_2CO_2C\mathit{H_2}CH_2$), 4.07 (m., $C\mathit{H_2}CO_2C$), 3.65-3.54 (m., $OC\mathit{H_2}C\mathit{H_2}O$), 3.37 ($COC\mathit{H_3}$), 2.29 (s., $C\mathit{H_3}COCH_2$), 2.09-1.17 (m., $C\mathit{H_2}CCH_3$), 1.01-0.86 (m., $C\mathit{H_2}CCH_3$). ^{13}C NMR (100 MHz, $CDCl_3$): **δ** (ppm) = 30.42, 44.90, 45.26, 49.75, 59.12, 64.03, 68.58, 70.69, 72.05.

*Synthesis of the Hydroxo-Bridged Iridium(III) Dimer Tetrakis(2-phenylpyridinato-N,C2)(m-dihydroxy)diiridium(III) ([Ir(ppy)$_2$OH]$_2$) (**C$_1$**)*

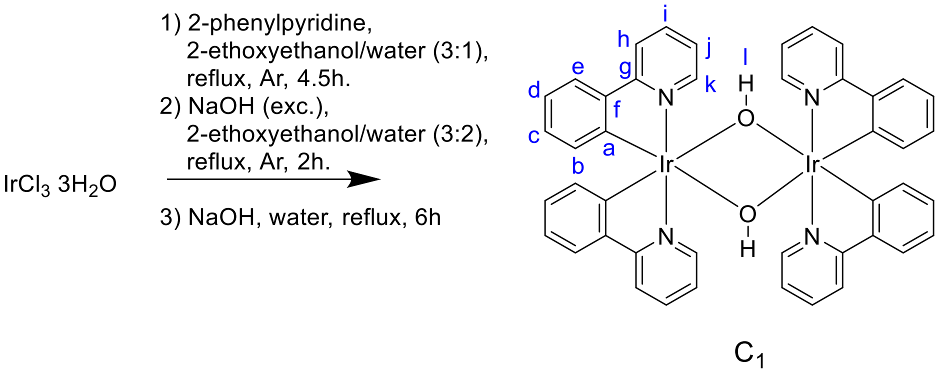

The dimeric complex **C1** was synthesized according to a procedure reported in literature.[1] A mixture of 257 mg (0.73 mmol) of $IrCl_3\ 3H_2O$, 276 mg (1.71 mmol) of 2-phenyl-pyridine in 12 ml of a solution of 2-ethoxy-ethanol/water (3:1) was refluxed under argon for 4.5 h. After this time, an excess of NaOH (1.25 g, 30 mmol) dissolved in 12.5 mL of H_2O was added and the resulting mixture was left stirring under reflux for 2 h. After cooling to r.t., 25 mL of H_2O was added. An orange-brown precipitate was filtered off, dissolved in 15 mL methylene chloride and, subsequently, filtered. The filtrate was treated with a NaOH solution (1.68 g, 0.04 mol in 4.5 mL H_2O) at reflux for 6 h. Afterwards, the organic solvent (CH_2Cl_2) was evaporated, and H_2O (125 mL) was added. The crude product was filtered off and washed with *n*-pentane (10 mL) and diethyl ether (10 mL). Further purification was carried out by precipitation (CH_2Cl_2 solution) in *n*-pentane, yielding $[Ir(ppy)_2OH]_2$ (**C1**) as a brown powder (295 mg, 78%). ^1H NMR (400 MHz, $CDCl_3$): δ (ppm) = 9.4-9.23 (m, 1H, H^k), 8.69-8.47 (m, 1H, H^h), 7.9-7.45 (m, 2H, $H^{e,i}$), 6.84-6.51 (m, 3H, $H^{c,d,j}$), 6.02-5.83 (m, 1H, H^b), –1.54 (s, 1H, H^l).

*Synthesis of the Cyclometalated Complex Bis[2-(2-pyridinyl-N)phenyl-C](methyl acetoacetato)iridium(III) (**C2**)*

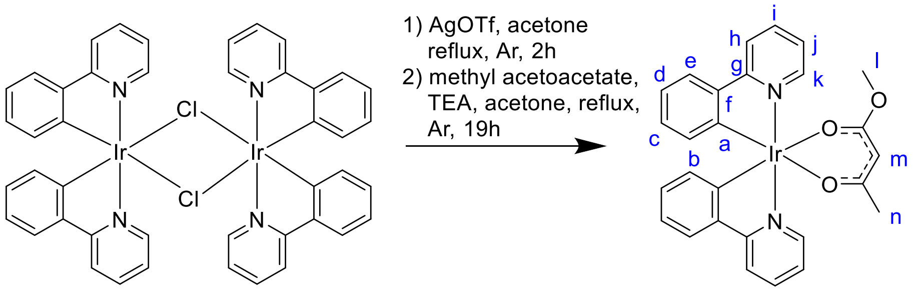

$[Ir(ppy)_2Cl]_2$ C_2

The complex **C2** was synthesized as follows and according to a procedure for the preparation of the analogue bis[2-(2-pyridinyl-N)phenyl-C](acetylacetonato)iridium(III), which is well described in literature.[2] Specifically, 117.9 mg (0.11 mmol) of $[Ir(ppy)_2Cl]_2$ and 84 mg (0.32 mmol) AgOTf were dissolved, in this order, in 8 mL of degassed acetone and refluxed at 55ºC under nitrogen atmosphere and continuous stirring for 2 h. The solution was cooled to r.t. and filtered to remove AgCl. The filtrate was refluxed under nitrogen atmosphere for 1 h and added under inert atmosphere to a 1 h refluxed solution of methyl acetoacetate (46 µl, 0.43 mmol) and triethylamine (113 µl, 0.81 mmol) dissolved in degassed acetone (4 ml), The resulting bright-brown solution was refluxed overnight under nitrogen atmosphere. The crude was then cooled to r.t. and filtered on cotton to eliminate last residues of AgCl. The volatiles were removed and the solid was sonicated in deionized water and

centrifuged for three consecutive times. The obtained solid was then dried under vacuum at r.t. for three days, affording the desired product **C2**. Yield % (weight %): 85 %. ^{1}H NMR (400 MHz, $CDCl_3$): δ (ppm) = 8.64-8.53 (m, 2H, H^k), 7.90-7.84 (m, 2H, H^h), 7.79-7.73 (m, 2H, H^i), 7.58-7.53 (m, 2H, H^e), 7.21-7.15 (m, 2H, H^j), 6.86-6.80 (m, 2H, H^d), 6.73-6.86 (m, 2H, H^c), 6.29-6.25 (m, 2H, H^b), 4.72 (s, 1H, H^m), 3.40 (s, 3H, H^l), 1.83 (s, 3H, H^n). ^{13}C NMR (100 MHz, $CDCl_3$): δ (ppm) = 28.73, 46.67, 51.28, 83.19, 99.95, 118.10, 118.39, 119.18, 120.60, 120.69, 121.42, 121.52, 123.49, 123.87, 128.78, 132.92, 133.10, 136.75, 136.96, 148.62, 148.80, 168.73, 186.37.

*Synthesis of Iridium(III)-Decorated Copolymers at Different Iridium(III) Loadings (**P_1-Ir_{40}**, **P_1-Ir_{23}** and **P_1-Ir_{10}**)*

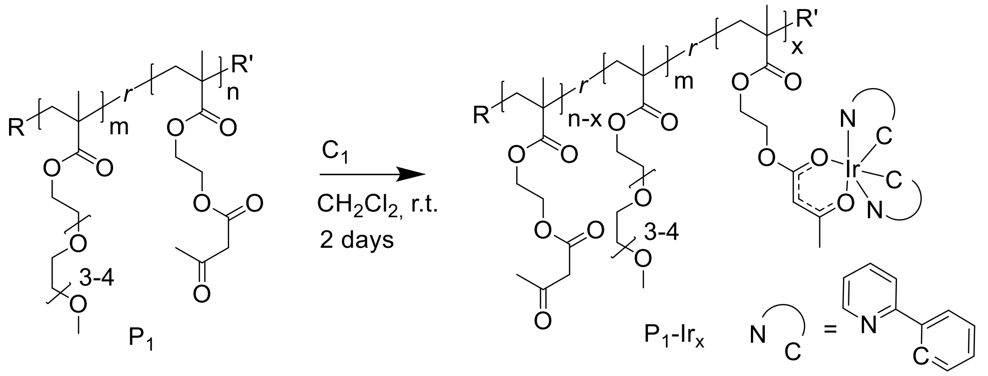

50 mg (0.707 mmol of AEMA) of polymer precursor **P1** and 18.6 mg (17.7 µmol) of **C1** were dissolved in 2 mL of anhydrous chloroform. The mixture was then degassed for 10 minutes and left stirring at r.t. under inert atmosphere and in the dark for 2 days. After this time, the crude was filtered and the volatiles were removed fluxing nitrogen, until a deep yellowish-brown film was obtained. **P1-Ir40**: Iridium(Ir)(III) loading, L^{Ir} (mol%) = 40, M_w (kDa) = 211.7, PDI = 1.03. **P1-Ir23** was obtained following the same procedure reported above, employing 50 mg of polymer precursor **P1** and 9.3 mg (8.8 µmol) of **C1**. **P1-Ir23**: L^{Ir} (mol%) = 23, M_w (kDa) = 174.5, PDI = 1.13. **P1-Ir10** was obtained following the same procedure reported above, employing 50 mg of polymer precursor **P1** and 4 mg (3.8 µmol) of **C1**. L^{Ir} (mol%) = 10, M_w (kDa) = 178.6, PDI = 1.10. ^{1}H NMR (400 MHz, $CDCl_3$) (**P1-Ir40**): δ (ppm) = 8.55-8.50 (m, 1 H), 7.84-7.75 (m, 1 H), 7.50 (m, 2H), 7.20 (m, 1 H), 6.77 (m, 1 H), 6.63 (m, 1 H), 6.19 (m, 1 H), 4.73 (m, 1 H), 4.31 (m., $CH_3COC\mathit{H_2}CO$), 4.13 (m., $CH_2CO_2C\mathit{H_2}CH_2$), 4.05 (m., $C\mathit{H_2}CO_2C$), 3.62-3.51 (m., $OC\mathit{H_2}C\mathit{H_2}O$), 3.34 ($COC\mathit{H_3}$), 2.27 (s., $C\mathit{H_3}COCH_2$), 2.09-1.78 (m., $C\mathit{H_2}CCH_3$), 0.99-0.85 (m., $C\mathit{H_2}CCH_3$). ^{13}C NMR (100 MHz, $CDCl_3$): **δ** (ppm) = 30.79, 45.46, 45.76, 50.30, 59.69, 64.60, 69.14, 71.25, 72.60.

*Preparation of Artificial Photo-Synthases based on Iridium(III)-Decorated Single Chain Nanoparticles in Aqueous Solutions (**APS-Ir$_{40}$, APS-Ir$_{23}$, APS-Ir$_{10}$**)*

As a general procedure, aqueous solutions of non-covalent, self-folded iridium(III) single chain nanoparticles at different metal loadings (**APS-Ir$_{40}$**, **APS-Ir$_{23}$**, **APS-Ir$_{10}$**) were obtained *via* direct dissolution of a film of the polymeric precursors (**P$_1$-Ir$_{40}$**, **P$_1$-Ir$_{23}$** and **P$_1$-Ir$_{10}$**) in water. In a typical procedure, 200 µL of a stock solution of iridium(III)-decorated copolymer (respectively **P$_1$-Ir$_{40}$**, **P$_1$-Ir$_{23}$** and **P$_1$-Ir$_{10}$**) in methylene chloride ([polymer] = 10 mg mL^{-1}) were left stirring at r.t. and in the dark for 2 h and until complete removal of the solvent, which was verified *via* gravimetry. The resulting deep-yellow polymeric film was then put in contact with a certain amount of deionized water to reach the desired final polymer concentration, and left stirring at r.t. overnight. After this time, the obtained transparent solutions were analyzed *via* DLS and UV-Vis spectrophotometry.

2.2. *"In Water"* [2+2] Photocycloaddition of Vinyl Arenes

General Procedure

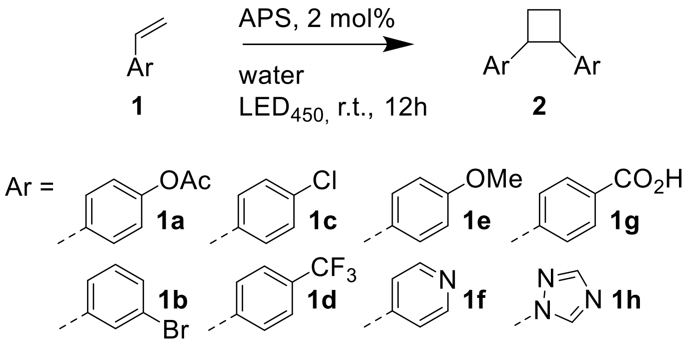

To a 4 mL oven dried vial, equipped of a magnetic stir bar, 100 µL of a stock solution of polymeric carrier **P$_1$-Ir$_{40}$** in methylene chloride ([polymer] = 20 mg mL^{-1}) was added. The polymer solution was then left stirring at r.t. and in the dark for 1 h, ensuring complete removal of the volatiles. Once a polymeric thin film was obtained, 1 mL of deionized water was added and the resulting mixture was then left stirring at r.t. and in the dark until a clear, pale-yellow solution was obtained. After adding 58 µmol of vinyl arene, the reaction was left open and stirring at r.t. and under LED illumination (λ_{max} = 450 nm) for 12 hours. After this time, 1 mL of saturated aqueous ammonium chloride was added to the mixture and the organic products were extracted by three consecutive times with 3 mL of diethyl ether each. The collected organic fractions were dried over anhydrous magnesium sulfate, filtered, and concentrated under reduced pressure. The extracted crude was then either purified via

silica gel column chromatography to afford the title compound **2a** (*n*-hexane/ethyl acetate 10:1 to 1:1) or directly diluted with 0.6 mL of $CDCl_3$ for quantitative NMR analysis.

The compounds **2b**, **2c**, **2d**, **2e**, **2f**, were isolated by following the general procedure. The NMR interpretations of the extracted reaction crudes NMR spectra resulted consistent with expected products according to literature data.[3, 4, 5]

2a: Isolated yield (weight %) = 90 %. NMR conversion (mol%) = 96 %. Trans/cis ratio = 1:0.3. ^{1}H NMR (400 MHz, $CDCl_3$): δ (ppm) = 7.23-7.21 (d, 4H, $CH_2CHCC\mathit{H}^{Ar}$), 7.01-6.99 (d, 4H, $OCC\mathit{H}^{Ar}$), 6.93-6.90 (d, 0.66H, $CH_2CHCC\mathit{H}^{Ar}$), 6.83-6.81 (d, 0.66H, $OCC\mathit{H}^{Ar}$), 4.01-3.97 (m, 0.4H, $CH_2C\mathit{H}C$), 3.56-3.52 (m, 2H, $CH_2C\mathit{H}C$), 2.46-2.31 (m, 2.5H, $C\mathit{H}_2C\mathit{H}_2$), 2.29 (s, 6H, CH_3), 2.23 (s, 1H, CH_3), 2.11 (m, 2.2H, $C\mathit{H}_2C\mathit{H}_2$). ^{13}C NMR (100 MHz, $CDCl_3$): δ (ppm) = 21.29, 24.45, 26.14, 29.86, 44.85, 47.55, 120.88, 121.47, 128.96, 142.06, 149.08, 169.81.

2b: NMR conversion (mol%) = 97 %. Trans/cis ratio = 1:0.2. ^{1}H NMR (400 MHz, $CDCl_3$): δ (ppm) = 7.39-7.34 (m, 4H, $C\mathit{H}CBrC\mathit{H}$), 7.20-7.15 (m, 5H, $C\mathit{H}C\mathit{H}CC$ and *cis*-$C\mathit{H}CBrC\mathit{H}$), 7.13-83 (m, 1.1H, *cis*-$CCC\mathit{H}C\mathit{H}$), 4.02-3.98 (m, 0.7H, *cis*-$C\mathit{H}CC$), 3.57-3.50 (m, 2H, *trans*-$C\mathit{H}CC$), 2.52-2.30 (m, 3.4H, *cis*-$C\mathit{H}_2C\mathit{H}_2$ and *trans*-$CH_2C\mathit{H}_2$), 2.20.2.08 (m, 2H, *trans*-$CH_2C\mathit{H}_2$).

2c: NMR conversion (mol%) = 96 %. Trans/cis ratio = 1:0.3. ^{1}H NMR (400 MHz, $CDCl_3$): δ (ppm) = 7.29-7.27 (m, 4H, $C\mathit{H}CCl$), 7.16-7.14 (m, 2H, $ClCCHC\mathit{H}$), 7.11-7.09 (m, 1.2H, *cis*-$C\mathit{H}CCl$), 6.88-6.86 (m, 1.2H, *cis*-$ClCCHC\mathit{H}$), 4.01-3.97 (m, 0.6H, *cis*-$CC\mathit{H}CH_2$), 3.53-3.45 (m, 2H, *trans*-$CC\mathit{H}CH_2$), 2.54-2.28 (m, 3.3H, $C\mathit{H}_2C\mathit{H}_2$), 2.17-2.07 (m, 2H, $CH_2C\mathit{H}_2$).

2d: NMR conversion (mol%) = 90 %. Trans/cis ratio = 1:0.3. ^{1}H NMR (400 MHz, $CDCl_3$): δ (ppm) = 7.57-7.55 (m, 4H, *trans*-$F_3CCC\mathit{H}$), 7.36-7.30 (m, 5.2H, *cis*-$F_3CCC\mathit{H}$ and *trans*-$C\mathit{H}^{Ar}CC$), 7.03-7.01 (m, 1.2H, *cis*-$C\mathit{H}^{Ar}CC$), 4.13-4.09 (m, 0.7H, *cis*-$CC\mathit{H}CH_2$), 3.67-3.59 (m, 2H, *trans*-$CC\mathit{H}CH_2$), 2.60-2.43 (m, 3.6H, $C\mathit{H}_2C\mathit{H}_2$), 2.42-2.14 (m, 2H, $C\mathit{H}_2C\mathit{H}_2$).

2e: NMR conversion (mol%) = 60 %. Trans/cis ratio = 1:0.3. ^{1}H NMR (400 MHz, $CDCl_3$): δ (ppm) = 7.17-7.15 (m, 4H, *trans*-$MeOCHC\mathit{H}$), 7.04-7.02 (m, 1.6H, *cis*-$MeOCHC\mathit{H}$), 6.93-6.91 (m, 1.6H, *cis*-$MeOC\mathit{H}$), 6.86-6.84 (m, 4H, *trans*-$MeOC\mathit{H}$), 3.92 (s, 2.1H, *cis*-$OC\mathit{H}_3$), 3.80 (s, 6H, *trans*-$OC\mathit{H}_3$), 3.46 (m, 2H, *trans*-$CC\mathit{H}CH_2$), 2.47-2.22 (m, 3.4H, $C\mathit{H}_2C\mathit{H}_2$), 2.14-2.03 (m, 3H, $C\mathit{H}_2C\mathit{H}_2$).

2f: NMR conversion (mol%) = 79 %. Trans/cis ratio = 1:0.3. ^{1}H NMR (400 MHz, $CDCl_3$): δ (ppm) = 8.55-8.53 (m, 4H, *trans*-$NC\mathit{H}$), 8.35-8.33 (m, 1.8H, *cis*-$NC\mathit{H}$), 7.15-7.13 (m, 4H, *trans*-$NCHC\mathit{H}$), 6.88-6.87 (m, 18H, *cis*-$NCHC\mathit{H}$), 4.06 (m, 1H, cis-$CC\mathit{H}CH_2$), 3.59 (m, 1H, *trans*-$CC\mathit{H}CH_2$), 2.59-2.44 (m, 2.30H, *cis*-$C\mathit{H}_2C\mathit{H}_2$), 2.41 (m, 2H, *trans*-$C\mathit{H}_2C\mathit{H}_2$), 2.22 (m, 2H, *trans*-$C\mathit{H}_2C\mathit{H}_2$).

2.3. *"In Water"* Oxidation of 9-Substituted Anthracenes

General Procedure

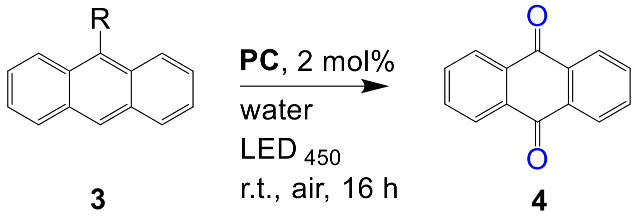

R = CH_2OH (**3a**), CH_2Cl (**3b**)

*Photocatalytic SCNPs-Mediated oxygenation of anthracenes in Water, general procedure (**OA-1**).* To a 4 mL oven dried vial, equipped of a magnetic stir bar, was added 100 µL of a stock solution of polymeric carrier **P$_1$-Ir$_{40}$** in methylene chloride ([polymer] = 20 mg/mL). The polymer solution was then added of 29 µmol of **3** and the resulting mixture was then left stirring at r.t. and in the dark for 2 hours and until all the volatiles were removed. After adding 1 mL of deionized water, the reaction was left open and stirring at r.t. and under LED illumination (λ_{max} = 450 nm) for 16 hours. After this time, the mixture was added of 1 mL of saturated aqueous ammonium chloride and extracted three consecutive times with 3 mL of ethyl acetate each. The collected organic fractions were dried over anhydrous magnesium sulfate, filtered, and concentrated under reduced pressure. The extracted crude was then either purified via silica gel column chromatography to afford the title compound **4** (*n*-hexane/ethyl acetate 2:1) or directly diluted with 0.6 mL of $CDCl_3$ for quantitative NMR analysis. Spectral data agree with literature data.[6,7,8] Isolated yield (weight %) = 58 %. Conversion NMR (mol%) = 62 %. ^{1}H NMR (400 MHz, $CDCl_3$): **δ** (ppm) = 8.35-8.31 (m, 4H, OCCCH^{Ar}), 7.83-7.79 (m, 4H, CCCH^{Ar}). ^{13}C NMR (100 MHz, $CDCl_3$): **δ** (ppm) = 127.39, 134.28

2.4. *"In Water"* α-Arylation of Arylamines

General Procedure

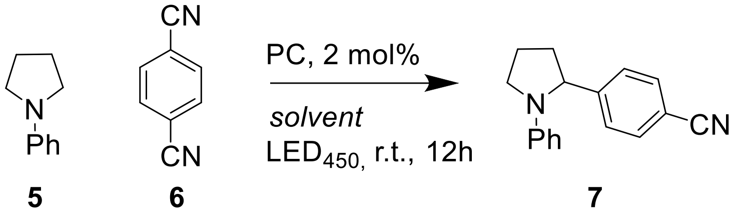

(AA-5). To a 4 mL oven dried vial, equipped of a magnetic stir bar, was added 100 µL of a stock solution of polymeric carrier **P_1-Ir_{40}** in methylene chloride ([polymer] = 20 mg/mL). The polymer solution was then added of 3.7 mg (29 µmol) of 1,4-dicyanobenzene and 203 mg (2.47 mmol) of sodium acetate. The resulting mixture was then left stirring at r.t. and in the dark for two hours and until all the volatiles were removed, the vial was then sealed with a rubber septum. After three consecutive vacuum-evacuation/Argon backfill cycles, 1 mL of extensively degassed deionized water and 12.5 µL (87 µmol) of N-phenyl pyrrolidine were added under Argon positive pressure. The resulting mixture was left stirring at r.t. and under LED illumination (λ_{max} = 450 nm) for 12 hours. After this time, the mixture was extracted three times with 6 mL of ethyl acetate each. The collected organic fractions were dried over anhydrous magnesium sulfate, filtered, and concentrated under reduced pressure. The extracted crude was then either purified *via* silica gel column chromatography to afford the title compound **7** (*n*-hexane/ethyl acetate 10:1 to 2:1) or directly diluted with 6.5 mL of $CDCl_3$ for quantitative NMR analysis. Isolated yield (weight %) = 48 %. Conversion NMR (mol%) = 57 %. ^{1}H NMR (400 MHz, $CDCl_3$): **δ** (ppm) = 7.59 (2H, d., H^{Ar}), 7.34 (2H, d., H^{Ar}), 7.15 (2H, t., H^{Ar}), 6.68 (1H, t., H^{Ar}), 6.44 (2H, d., H^{Ar}), 4.74 (1H, d., NC*H*), 3.73 (1H, m., NCH_2), 3.43 (1H, m., NCH_2), 2.47-2.39 (1H, m., CHCH_2), 2.03-1.88 (3H, m., CHCH_2CH_2). ^{13}C NMR (100 MHz, $CDCl_3$): **δ** (ppm) = 23.29, 29.86, 36.03, 49.38, 62.93, 110.75, 112.58, 116.65, 119.12, 126.90, 129.29, 132.60, 146.84, 150.62, in agreement with literature data.[9, 10, 11]

AA-4 and **AA-10** were carried out according to the general procedure varying the salt amount in the reaction mixture, using 70 mg (58 µmol) or 1.2 g (14.6 mmol) of NaOAc respectively. **AA-9** was carried out following the general procedure, using 1 mL of binary solution water/DMA (DMA 10%) as solvent.

α-Arylation of N-Arylamines in Water. Non-supported catalyst procedure (AA-8). To a 4 mL oven dried vial, equipped of a magnetic stir bar, was added 0.36 mg (0.58 µmol) of **C_2**, 3.7 mg (29 µmol) of 1,4-dicyanobenzene and 203 mg (2.47 mmol) of sodium acetate. The vial was then sealed with a rubber septum. After three consecutive vacuum-evacuation/Argon backfill cycles, 1 mL of

extensively degassed deionized water and 12.5 µL (87 µmol) of N-phenyl pyrrolidine were added under Argon positive pressure. The resulting mixture was left stirring at r.t. and under LED illumination (λ_{max} = 450 nm) for 12 hours. After this time, the mixture was extracted three times with 6 mL of ethyl acetate each. The collected organic fractions were dried over anhydrous magnesium sulfate, filtered, and concentrated under reduced pressure. The extracted crude was then diluted with 6.5 mL of $CDCl_3$ for quantitative NMR analysis. No conversion to the desired product was observed.

*α-Arylation of N-Arylamines in Water. Procedure in organic solvents (**AA-1, AA-2, AA-3**).* An oven-dried 15 mL vial equipped with a rubber septum and a magnetic stir bar was charged with 0.5% mol of photocatalyst (3.08 mg of **C_2** for **AA-1** and **AA-3**, 50 µL of 20 mgmL^{-1} **P_1-Ir_{40}** stock solution in methylene chloride for **AA-2**), 128.1 mg (1 mmol) of 1,4-dicyanobenzene and 164,1 mg (2 mmol) of NaOAc. The Schlenk was then purged with 3 consecutive vacuum evacuation/argon backfill. Then, 4 mL of degassed solvent (DMA for **AA-1**, **AA-2**, MeOH for **AA-3**) was added under positive nitrogen pressure followed by 434 µL (3 mmol) of N-phenylpirrolidine. The reaction mixture was furtherly degassed via three cycles of vacuum evacuation/argon backfill. After degassing, the vial was sealed with parafilm and left under LED illumination (λ_{max} = 450 nm) for 12 hours. After this time, the reaction was then diluted with ethyl acetate and added to a separatory funnel containing 25 mL of a saturated Na_2CO_3 aqueous solution. The layers were separated, and the aqueous layer was extracted with EtOAc. The collected organic fractions were dried over anhydrous magnesium sulfate, filtered, and concentrated under reduced pressure. The extracted crude was then purified *via* silica gel column chromatography to afford the title compound **7** (*n*-hexane/ethyl acetate 10:1 to 2:1).

2.5. *"In water"* β-Hydroxysulfonylation of *α*-Methyl Styrene

General Procedure

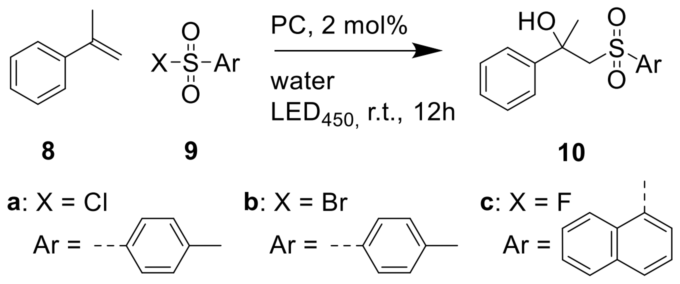

200 μL of a stock solution of polymeric carrier **P1-Ir23** in methylene chloride ([polymer] = 20 mg/mL) and 9 mg (35 μmol) of tosyl chloride were put in an oven dried 4 mL vial, previously equipped with a magnetic stir bar. After complete removal of the volatiles, the vial was sealed with a rubber septum and the resulting polymeric film was degassed via three consecutive vacuum-pump/Argon-backfill cycles. 1 mL of extensively degassed deionized water and 3.4 mg (29 μmol) of α-methyl styrene were then added under Argon positive pressure. The resulting solution was left stirring at r.t. and under LED light (λ_{max} = 450 nm) irradiation for 12 hours. After this time, the crude was extracted three times with 6 mL of ethyl acetate each. The collected organic fractions were dried over anhydrous magnesium sulfate, filtered, and concentrated under reduced pressure. After solvent removal, the crude product was either purified by column chromatography on silica gel (*n*-hexane/ethyl acetate 4:1) to afford the title compound **10a** or added of a known amount of 1,4-dicyanobenzene and diluted with 0.6 mL of $CDCl_3$ for quantitative ^{1}H NMR analysis. Spectral data agree with literature data.[12] Isolated yield (weight %) = 58 %. Conversion NMR (mol%) = 67 %. ^{1}H NMR (400 MHz, $CDCl_3$): **δ** (ppm) = 7.49 (d, 2H), 7.29 – 7.27 (m, 2H), 7.18 (td, 5H), 4.63 (br.s., 1H), 3.71 – 3.57 (m, 2H), 2.39 (s, 3H), 1.71 (s, 3H). ^{13}C NMR (100 MHz, $CDCl_3$): **δ** (ppm) = 21.73, 29.85, 60.54, 62.31, 121.88, 126.38, 128.08, 128.51, 128.84, 129.65, 136.76, 142.43.

HS-1 was carried out according to the general procedure, using 100 μL of **P1-Ir40** stock solution in methylene chloride ([polymer] = 20 mg/mL). **HS-3** was carried out according to the general procedure, using 400 μL of **P1-Ir10** stock solution in methylene chloride ([polymer] = 20 mg/mL). **HS-4** was carried out following the general procedure using 8.2 mg (35 μmol) of paratoluensulfonyl bromide in substitution of the tosyl chloride. **HS-5** was carried out following the general procedure using 7.4 mg (35 μmol) of 2-naphtalenesulfonyl fluoride in substitution of tosyl chloride.

*β-Hydroxysulfonylation of Aromatic Alkenes. Non-supported catalyst procedure (**HS-6**).* To a 4 mL oven dried vial, equipped of a magnetic stir bar, was added 0.36 mg (0.58 µmol) of **C_2**, 9 mg (35 µmol) of tosyl chloride were put in an oven dried 4 mL vial, previously equipped with a magnetic stir bar. After complete removal of the volatiles, the vial was sealed with a rubber septum and the resulting polymeric film was degassed via three consecutive vacuum-pump/Argon-backfill cycles. 1 mL of extensively degassed deionized water and 3.4 mg (29 µmol) of α-methyl styrene were then added under Argon positive pressure. The resulting solution was left stirring at r.t. and under LED light (λ_{max} = 450 nm) irradiation for 12 hours. After this time, the crude was extracted three times with 6 mL of ethyl acetate each. The collected organic fractions were dried over anhydrous magnesium sulfate, filtered, and concentrated under reduced pressure. After solvent removal, the crude product was added of a known amount of 1,4-dicyanobenzene and diluted with 0.6 mL of $CDCl_3$ for quantitative ^{1}HNMR analysis.

HS-7 was carried out following the general procedure and without adding any photocatalyst. No conversion to the desired product was observed.

3. Effect of APS type on Conversion

To investigate the effect of APS type (*i.e.,* Ir(III)-Loading) on conversion (*c*%), we maintained fixed the overall catalyst concentration and observed the influence of the parameter ***d*** –which we call the "*intra-particle photocatalytic unit density"* (see Eq. S7)– on *c*%. Although for classical photocatalysis one would expect no substantial influence of ***d*** on *c%*, we observed that the dependence of *c*% on ***d*** varied with the selected reaction (Figure S1).

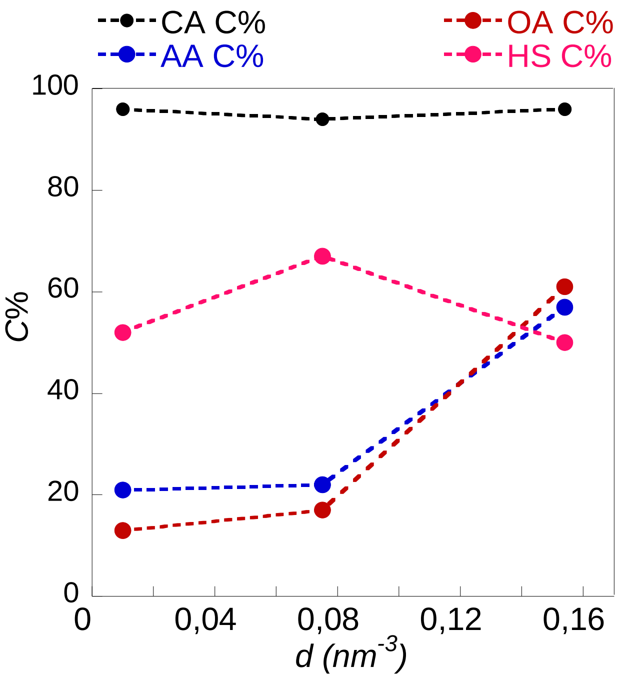

Figure S1. Conversion (*c*%) *vs.* "intra-particle photocatalytic unit density" (***d***) for the four kinds of reactions explored in this work. CA = [2+2] Cycloaddition of vinyl arenes. AA = α-Arylation of arylamines. OA = Oxidation of 9-substituted anthracenes. HS = α-Styrene β-hydroxysulfonylation.

We hypothesized this behavior could be due to the very different nature of the mechanisms involved, which we named as A-type or B-type. In A-type mechanism, two reactants independently interact with the photosensitizer, whereas in B-type mechanism the contact occurs either in parallel or one time only. Hence, APS showing high value of ***d*** would favor A-type mechanism (AA, OA) while B-type mechanism would be almost independent on ***d*** (CA, HS).

4. Kinetics assays of APS

To find the optimal experimental conditions for performing the APS catalytic turnover number estimation *via* initial velocities measurement, we first assessed the course of the "*in water*" [2+2] photocycloaddition of **1a** catalyzed by **APS-Ir$_{40}$**. Six reactions were prepared by adding to a 4 mL oven dried vial, equipped of a magnetic stir bar, 100 µL of a stock solution of **P$_1$-Ir$_{40}$** in methylene chloride ([polymer] = 20 mg mL^{-1}). The polymer solution was then left stirring at r.t. and in the dark for 1 h, ensuring complete removal of the volatiles. Once a polymeric thin film was obtained, 1 mL of deionized water was added and the resulting mixture was then left stirring at r.t. and in the dark until a clear, pale-yellow solution of **APS-Ir$_{40}$** was obtained. After adding 58 µmol of **1a**, the reactions were left open and stirring at r.t. and under LED illumination (λ_{max} = 450 nm), each for the desired amount of time (t_0, t_1, t_2, t_3, t_4 and t_5 for 0 min, 30 min, 1 h, 2 h, 3 h, 6 h, respectively). After these times, the organic products were extracted by three consecutive times with 3 mL of diethyl ether each. The collected organic fractions were dried over anhydrous magnesium sulfate, filtered, and concentrated under reduced pressure. The extracted crudes were then directly diluted with 0.5 mL of $CDCl_3$ for quantitative NMR analysis (see Figures S2 and S3).

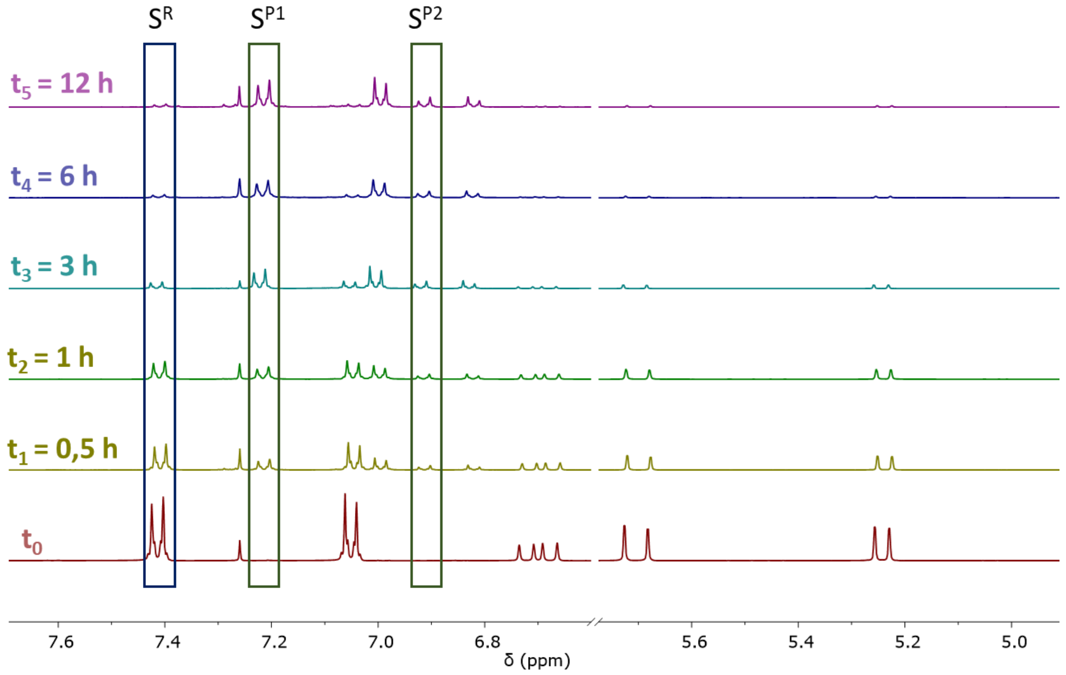

Figure S2. Superimposed ^{1}H NMR spectra used to follow the photoconversion of **1a** in **2a** catalyzed by **APS-Ir$_{40}$** over time. The highlighted portions of the figures show the signals S^{Pn} and S^R of **2a** and **1a**, respectively, used for conversion calculation (C% = 100 × mol **2a** / mol **1a**).

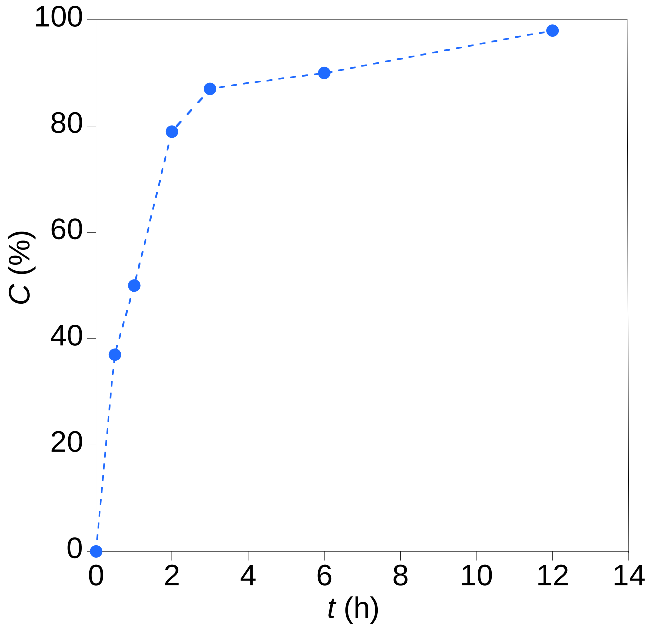

Figure S3. Conversion (%) *vs.* time of the "*in water*" [2+2] photocycloaddition of **1a** catalyzed by **APS-Ir$_{40}$**.

For the kinetics assay, six solutions of **APS-Ir$_{40}$** were first prepared following the above standard procedure. After adding 235 µmol, or 175 µmol, or 120 µmol, or 58 µmol, or 15 µmol, or 8 µmol of vinyl arene **1a**, the reaction mixtures were left open and stirring at r.t. and under LED illumination (λ_{max} = 450 nm). Each sample reaction at fixed substrate concentration was irradiated for each of the desired amount of time (t_0, t_1, t_2, t_3 and t_4 for 0 min, 5 min, 10 min, 20 min and 30 min, respectively). After these times, the organic products were extracted by three consecutive times with 3 mL of diethyl ether each. The collected organic fractions were dried over anhydrous magnesium sulfate, filtered, and concentrated under reduced pressure. The extracted crudes were then diluted with 0.5 mL of $CDCl_3$ containing 1,4-diaminobenzene 18.4 × 10^{-3} M for quantitative NMR analysis. The amount of **2a** at the time *t* in the reaction ([**2a**]$_t$) was determined according to the Equation S8. The reactions corresponding to lower substrate concentrations, namely 15 mM and 8 mM, were repeated in duplicate and the respective extracted crudes were summed for quantitative calculation *via* NMR.

$$[\mathbf{2a}]_t\ (\mathrm{M}) = \frac{n_{2a}^{i}\ (\mathrm{mol})}{V^r\ (\mathrm{L})} = \frac{\Sigma^P}{4} \times \frac{4}{I^{SI}}\ 9.22 \times 10^{-6} (\mathrm{mol}) \frac{1}{10^{-3}\ (\mathrm{L})} \qquad \text{Eq.S8}$$

where n^i_{2a} is the calculated number of moles of **2a** in the NMR tube, Σ^P is the sum of the integrals of the products (*trans*-**2a** and *cis*-**2a**) at 7.23 ppm and 6.93 ppm, respectively, V^r is the volume of the reaction mixture and I^{SI} is the integrated signal of the protons of the internal standard at 6.57 ppm.

After deriving the initial velocities v_0^i of the catalyzed reaction at each *i*-concentration of **2a** by linear fitting of the experimental points (see Figure S4), we fit the v_0^i values to a Michaelis-Menten curve (see Figure S5). [13]

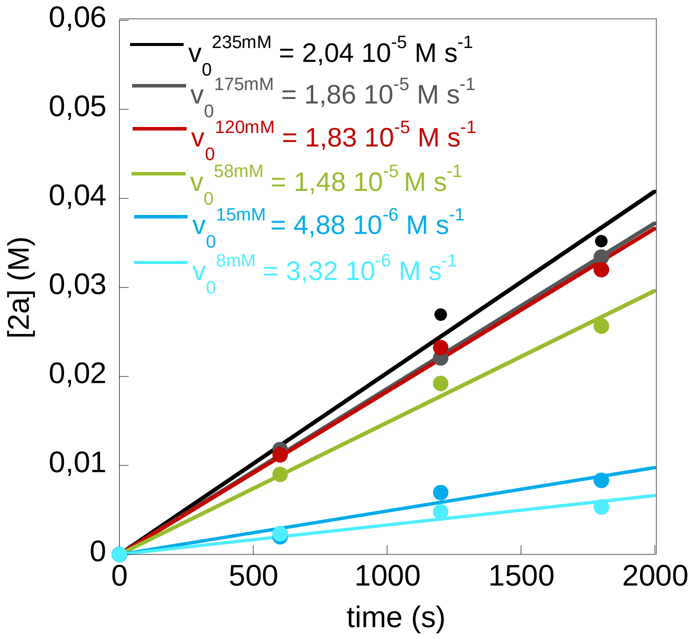

Figure S4. Initial velocities (^{1}H NMR) of the "*in water*" [2+2] photocycloaddition of **1a** catalyzed by **APS-Ir₄₀** in the substrate range between 0.008 M and 0.235 M.

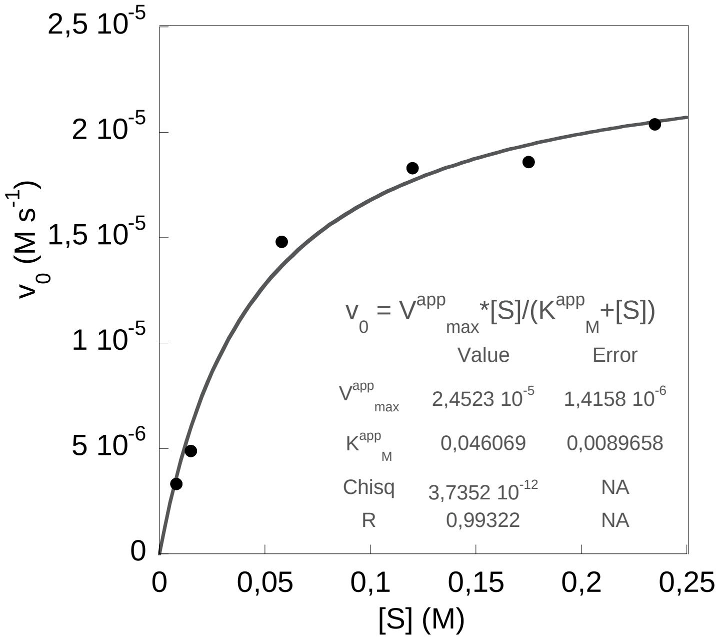

Figure S5. Michaelis-Menten plot of the "*in water*" [2+2] photocycloaddition of **1a** catalyzed by **APS-Ir$_{40}$**.

An apparent catalytic constant $k_{\mathrm{cat,app}}$ of 2.6 s^{-1} was obtained from Equation S9:

$$k_{\mathrm{cat,app}}(\mathrm{s}^{-1}) = \frac{\mathrm{V}_{\mathrm{max}}}{N_{\mathrm{T}}} \qquad \text{Eq.S9}$$

where V_{max} is the maximum measured velocity of conversion of **1a** into the title product **2a** and N_{T} is the nanoparticle concentration employed for the catalytic reaction, calculated taking the M_{w} (SEC) as molecular weight. The value of the apparent Michaelis-Menten constant obtained was $K_{\mathrm{M,app}} = 4.6 \times 10^{-2}$ M.

5. Recyclability of APS

After a 1st "*in water*" [2+2] photocycloaddition reaction of **1a** with **APS-Ir$_{40}$** as catalyst, the organic products were extracted by three consecutive times with 3 mL of diethyl ether each. The collected organic fractions were dried over anhydrous magnesium sulfate, filtered, and concentrated under reduced pressure. The extracted crudes were then directly diluted with 0.5 mL of $CDCl_3$ for quantitative NMR analysis. The aqueous phase was then collected and centrifuged at 4°C, the pale-yellow sediment was re-dispersed in 1 mL of deionized water by means of continuous stirring at r.t. and in the dark for 1 h. For the 2nd catalytic cycle, the resulting clear, faded-yellow solution was subsequently transferred to a 4 mL reaction vessel, charged with 58 μmol of vinyl arene **1a** and left open and stirring at r.t. and under LED illumination (λ_{max} = 450 nm) for 12 hours. After this time, the organic products were extracted by three consecutive times with 3 mL of diethyl ether each. The collected organic fractions were dried over anhydrous magnesium sulfate, filtered, and concentrated under reduced pressure. The extracted crudes were then directly diluted with 0.5 mL of $CDCl_3$ for quantitative NMR analysis. Next 3rd and 4th catalytic cycles were carried out following the same procedure as described for the 2nd one. Figure S6 illustrates the results of the recyclability of **APS-Ir$_{40}$** in 4 consecutive cycles.

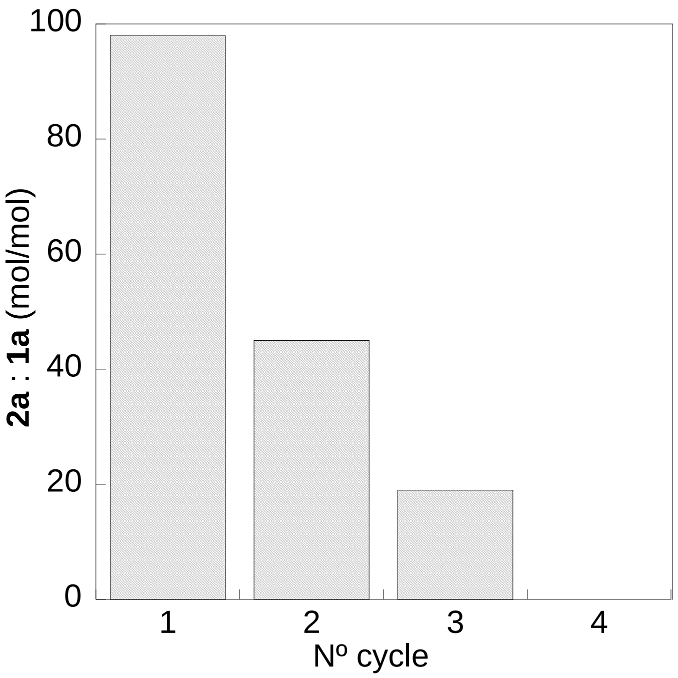

Figure S6. Results of the recyclability experiment of **APS-Ir$_{40}$** as catalyst of the "*in water*" [2+2] photocycloaddition of **1a**.

6. SEC Chromatograms

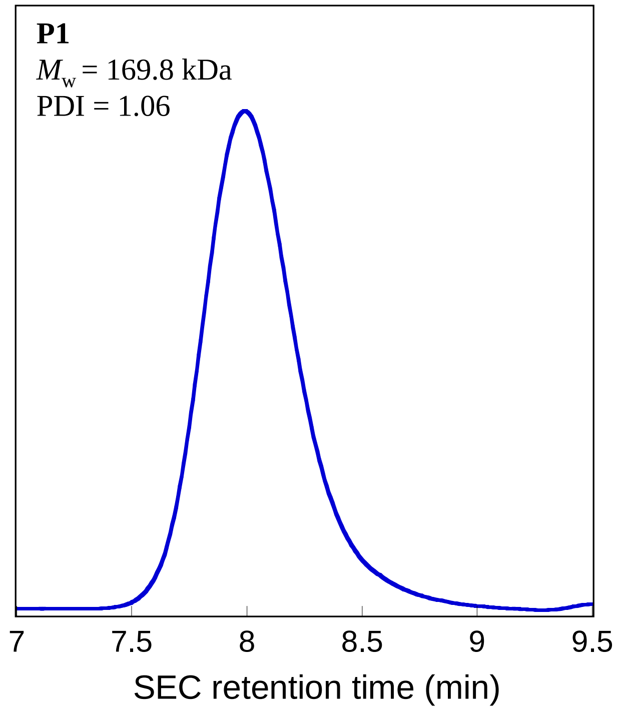

Figure S7. SEC chromatogram of copolymer $\mathbf{P_1}$ (DRI detector, THF, 1 mL min^{-1}).

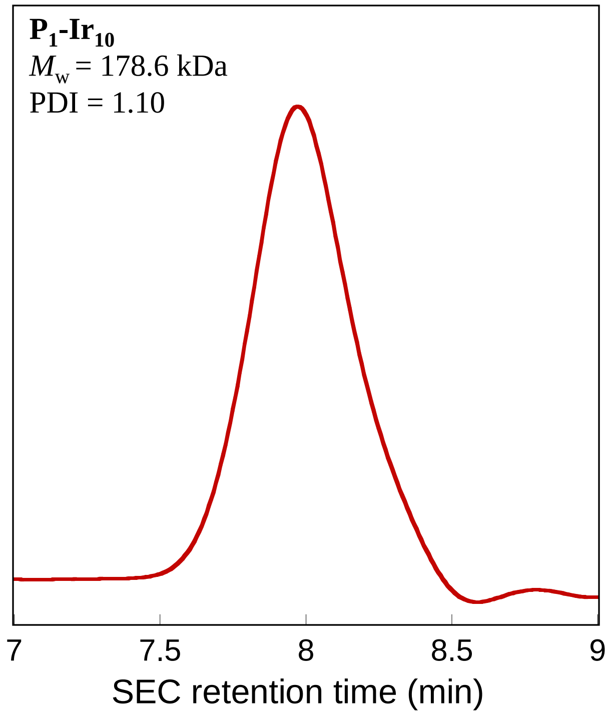

Figure S8. SEC chromatogram of copolymer $\mathbf{P_1}$-$\mathbf{Ir_{10}}$ (DRI detector, THF, 1 mL min^{-1}).

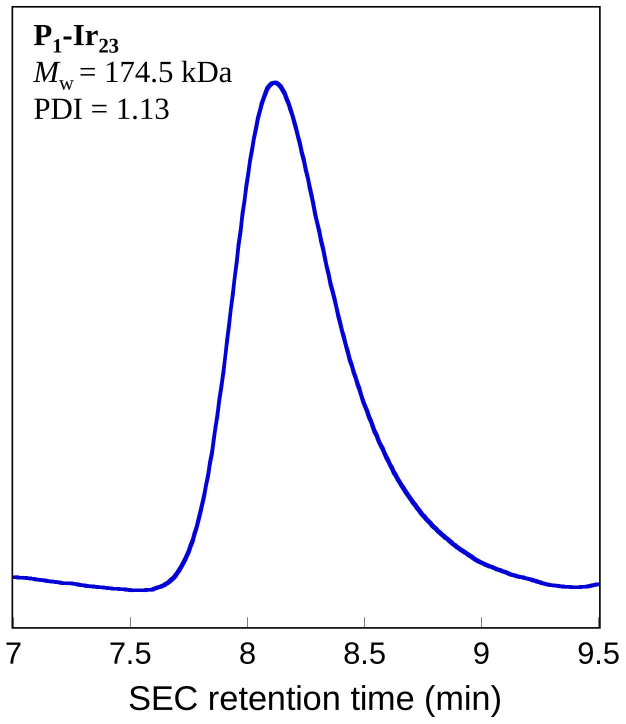

Figure S9. SEC chromatogram of copolymer **P1-Ir23** (DRI detector, THF, 1 mL min^{-1}).

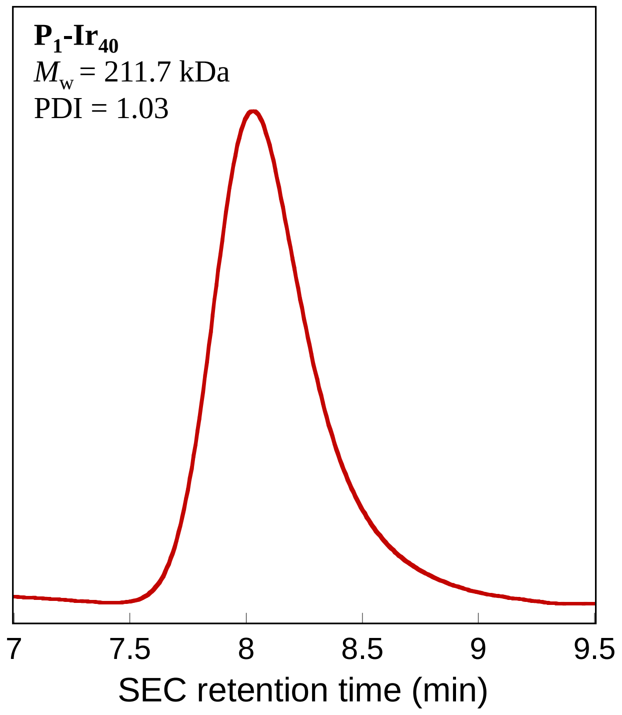

Figure S10. SEC chromatogram of copolymer **P1-Ir40** (DRI detector, THF, 1 mL min^{-1}).

7. DLS Data

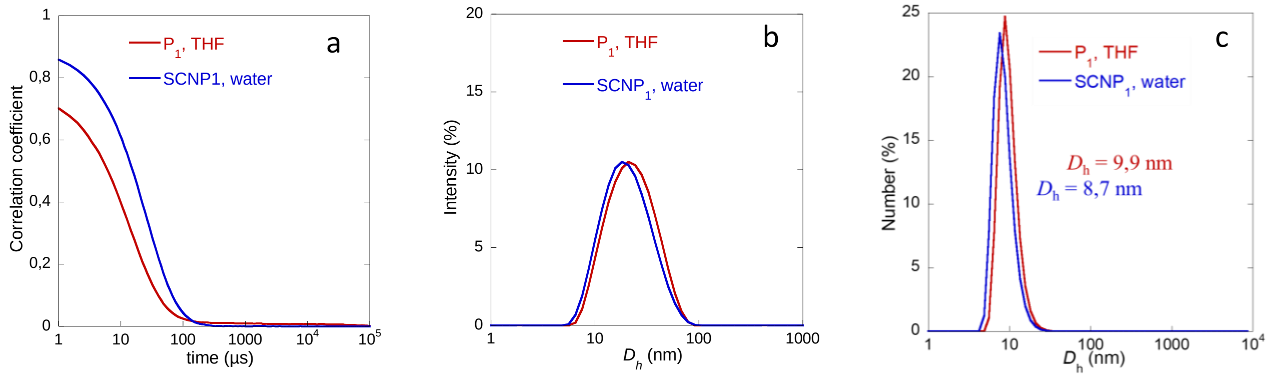

Figure S11. DLS size distributions of copolymer **P1** in THF (red line) and **SCNP-P1** water (blue line) at a concentration of 4 mg mL^{-1}: a) Correlation function. b) Intensity plot. c) Number plot.

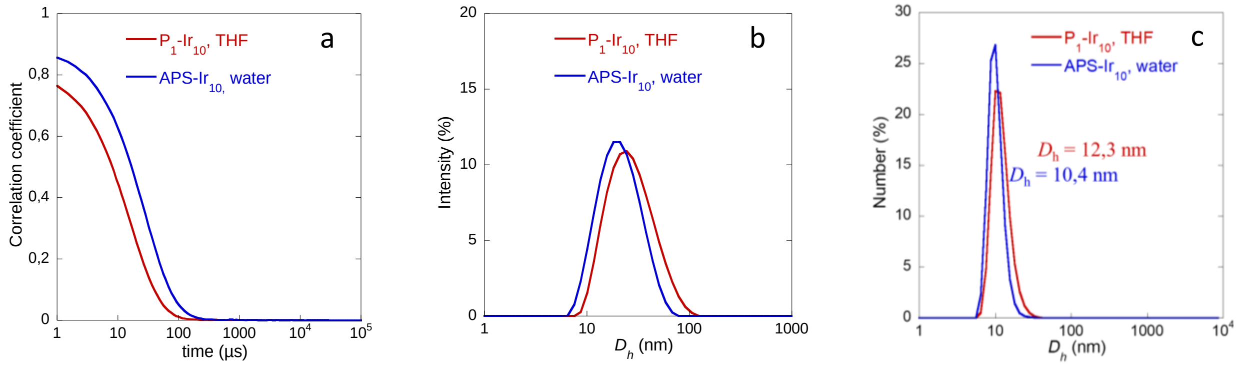

Figure S12. DLS size distributions of **P1-Ir10** in THF (red line) and **APS-Ir10** in water (blue line) at a concentration of 4 mg mL^{-1}: a) Correlation function. b) Intensity plot. c) Number plot.

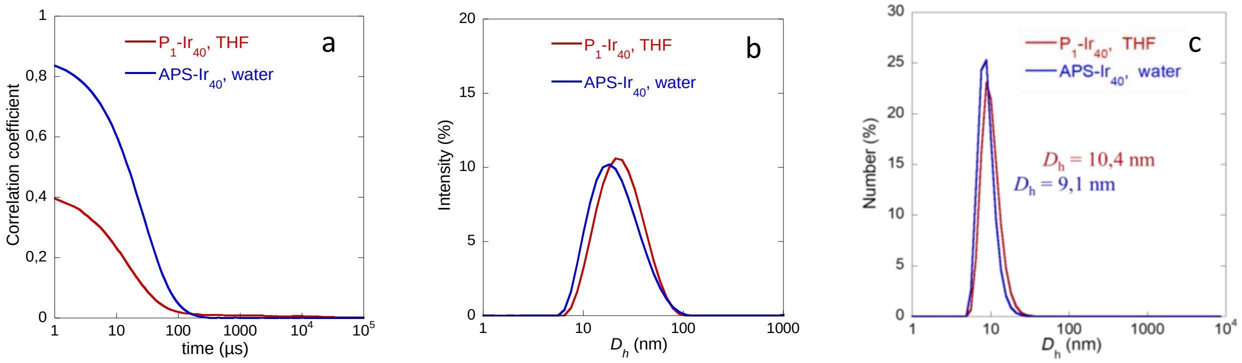

Figure S13. DLS size distributions of **P1-Ir40** in THF (red line) and **APS-Ir40** in water (blue line) at a concentration of 4 mg mL^{-1}: a) Correlation function. b) Intensity plot. c) Number plot.

8. UV-Vis Spectra

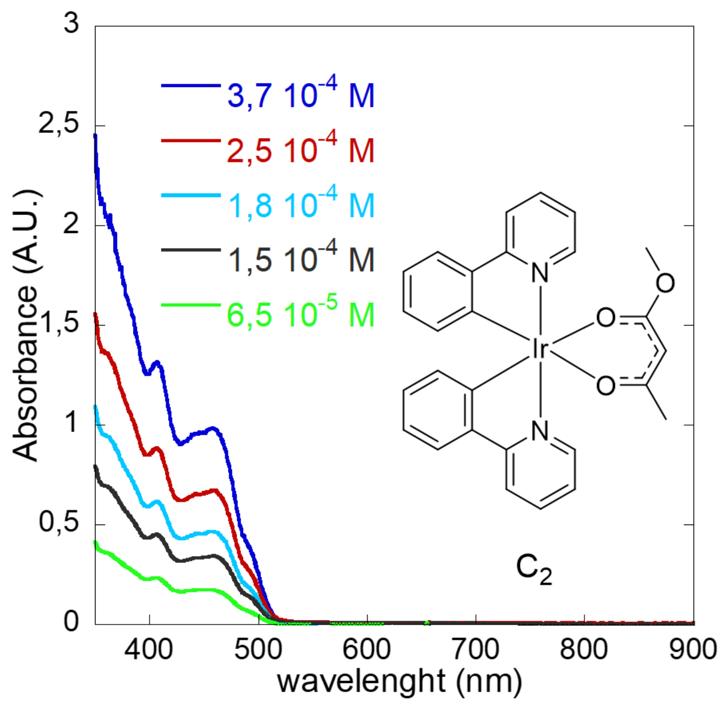

Figure S14. Superimposed absorbance spectra of five solutions of **C2** in chloroform at different concentration used for molar extinction coefficient (ε) calculation.

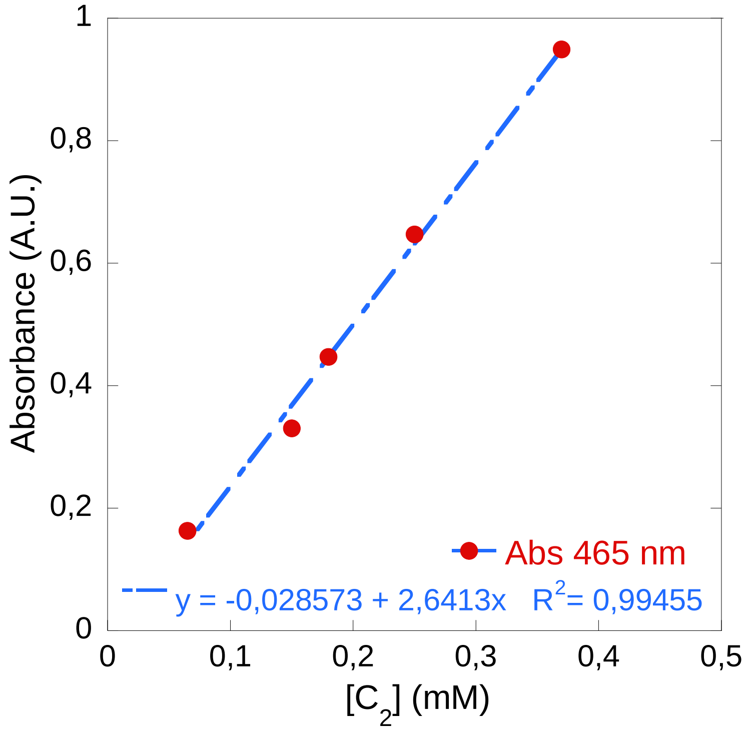

Figure S15. Calibration curve for the determination of the molar extinction coefficient ε of **C2** in chloroform at the wavelength λ_{max} = 465 nm (ε = 2641 cm M^{-1}).

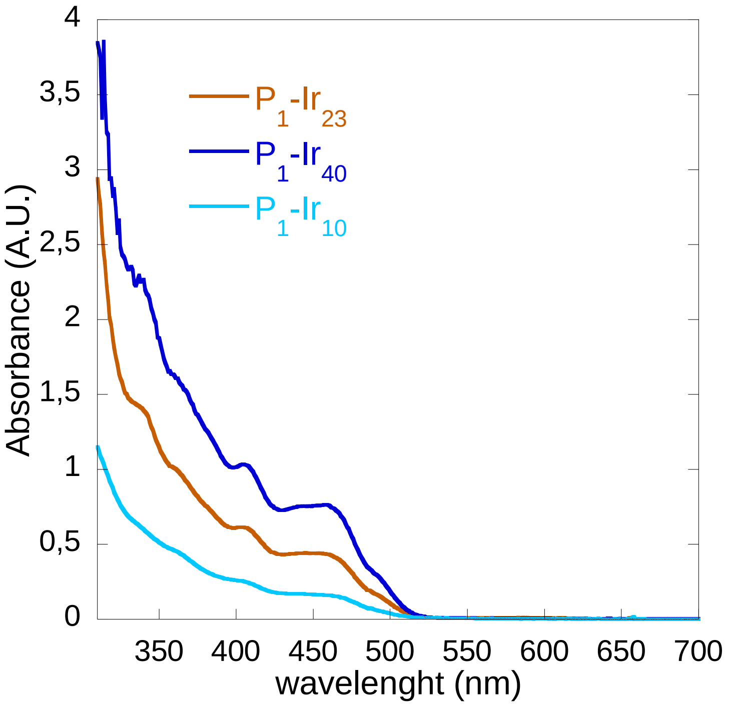

Figure S16. Superimposed absorbance spectra of **P_1-Ir_x** in chloroform all at a concentration of 1 mg mL^{-1}.

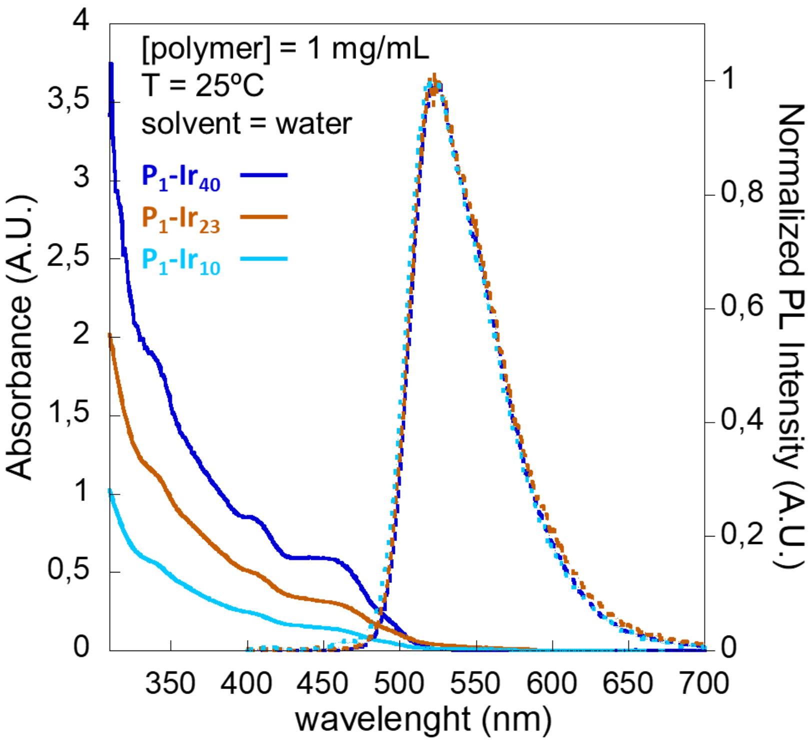

Figure S17. Superimposed absorbance spectra of **P_1-Ir_x** in water all at a concentration of 1 mg mL^{-1} and respective normalized emission spectra, recorded with a λ_{exc} = 365 nm.

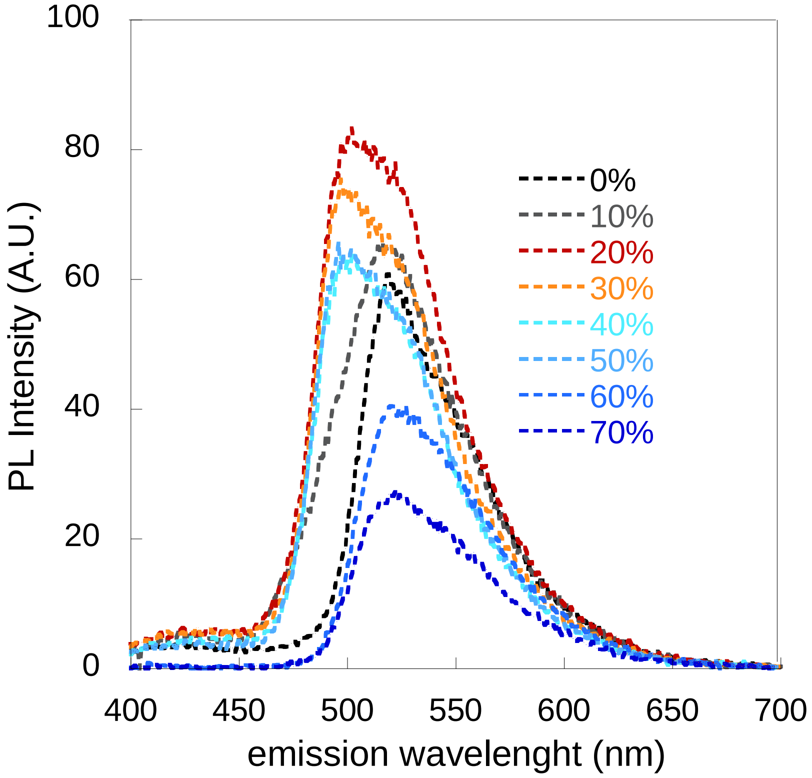

Figure S18. Superimposed emission spectra of seven **C2** ([**C2**] = 3 μM) degassed solutions in THF at increasing water amount added (v/v%).

9. NMR Spectra

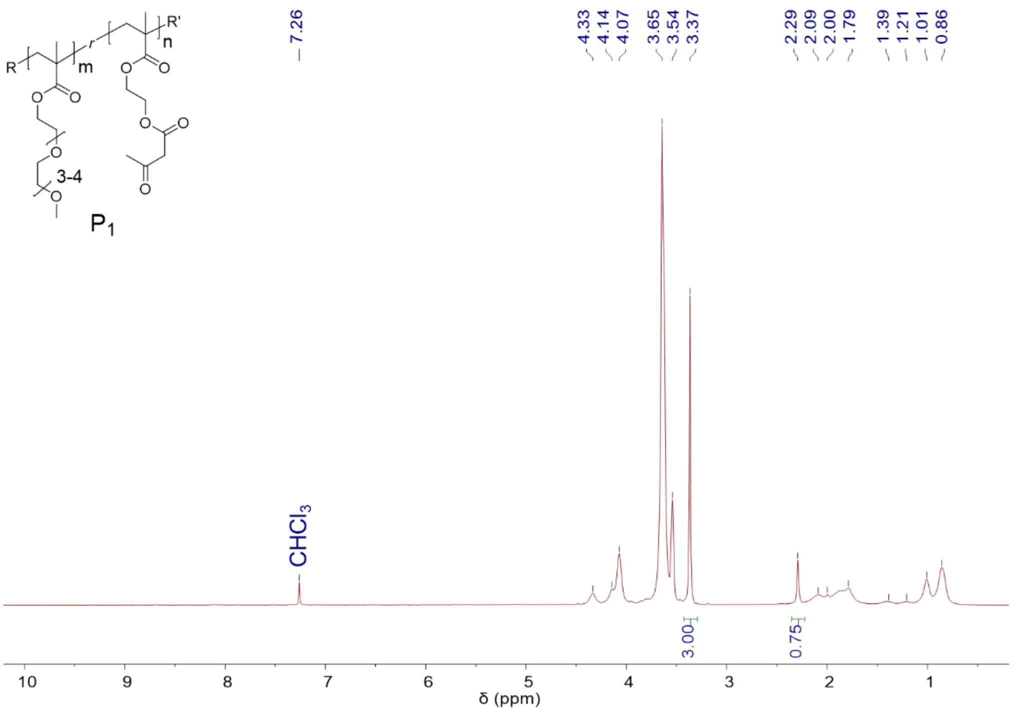

Figure S19. ^{1}H NMR spectrum of **P_1**.

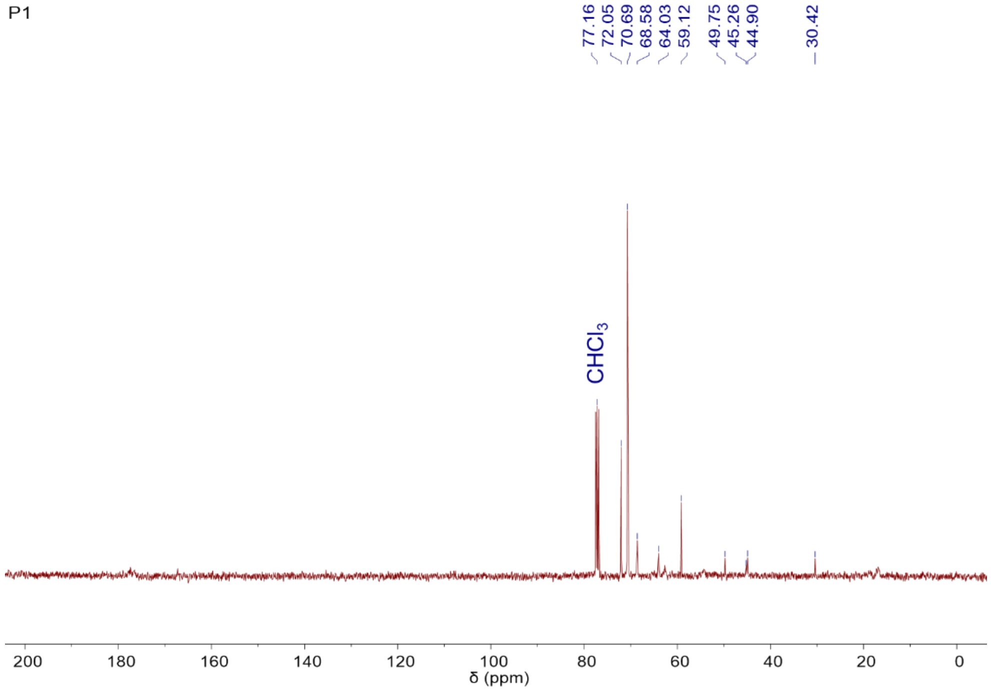

Figure S20. ^{13}C NMR spectrum of **P_1**.

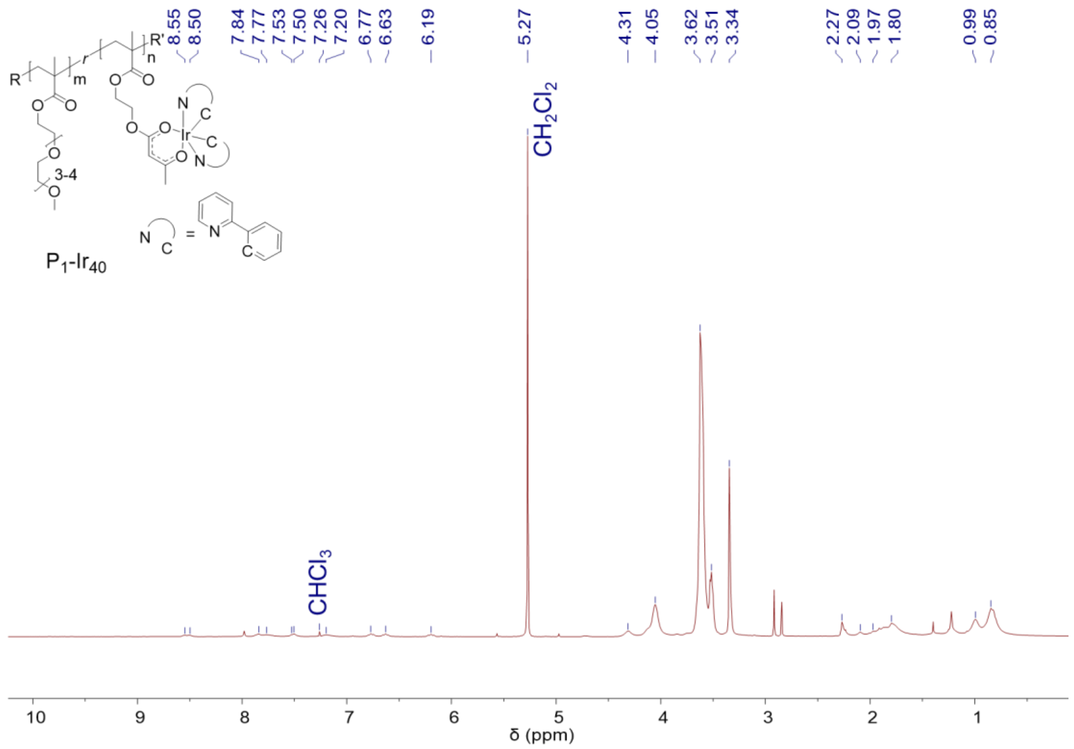

Figure S21. ^{1}H NMR spectrum of **P$_1$-Ir$_{10}$**.

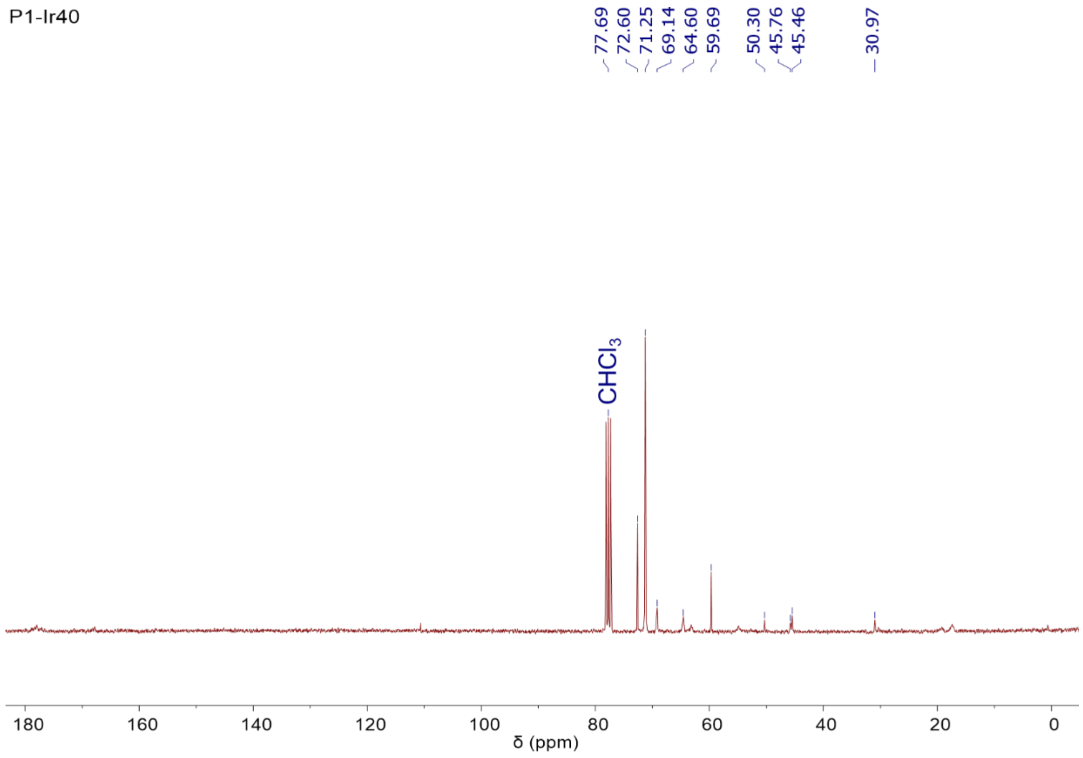

Figure S22. ^{13}C NMR spectrum of **P$_1$-Ir$_{10}$**.

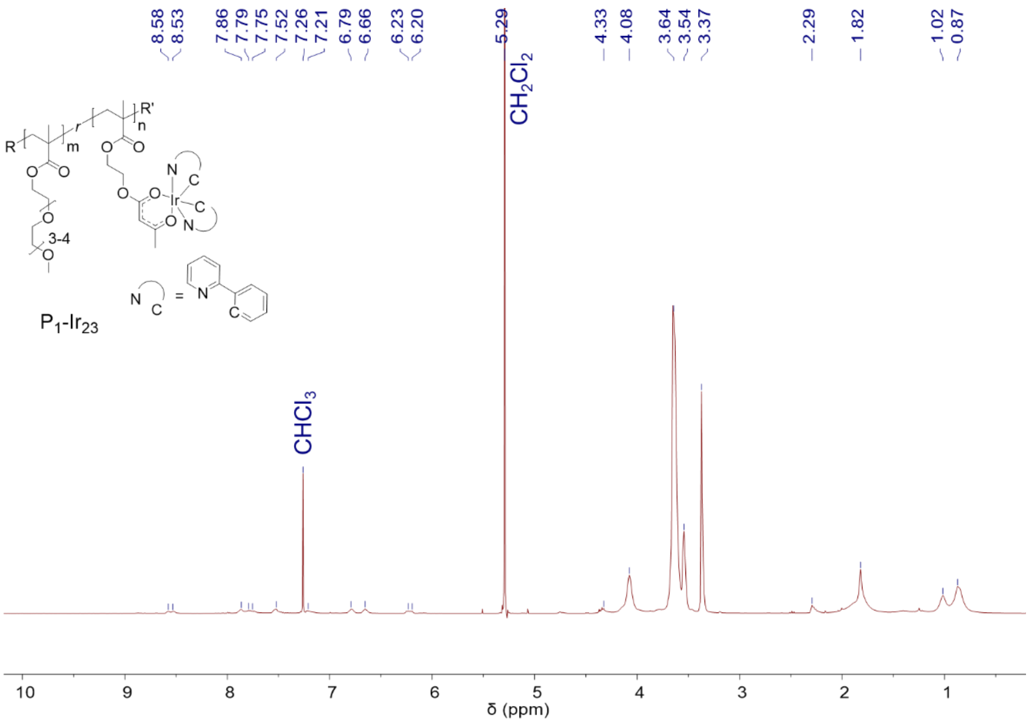

Figure S23. ^{1}H NMR spectrum of **P1-Ir23**.

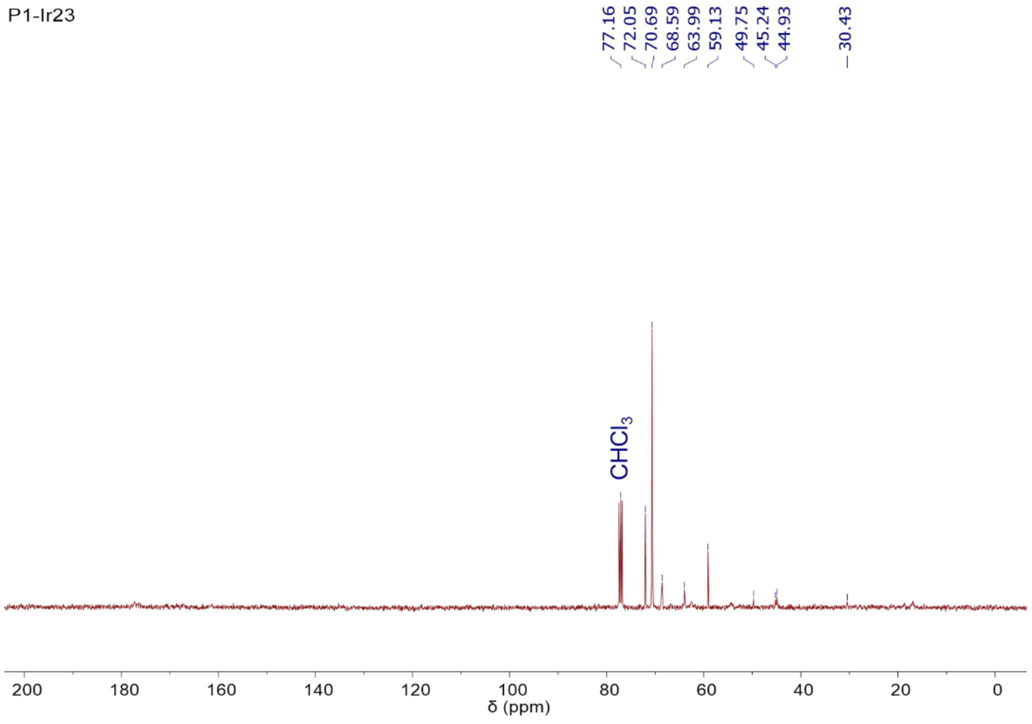

Figure S24. ^{13}C NMR spectrum of **P1-Ir23**.

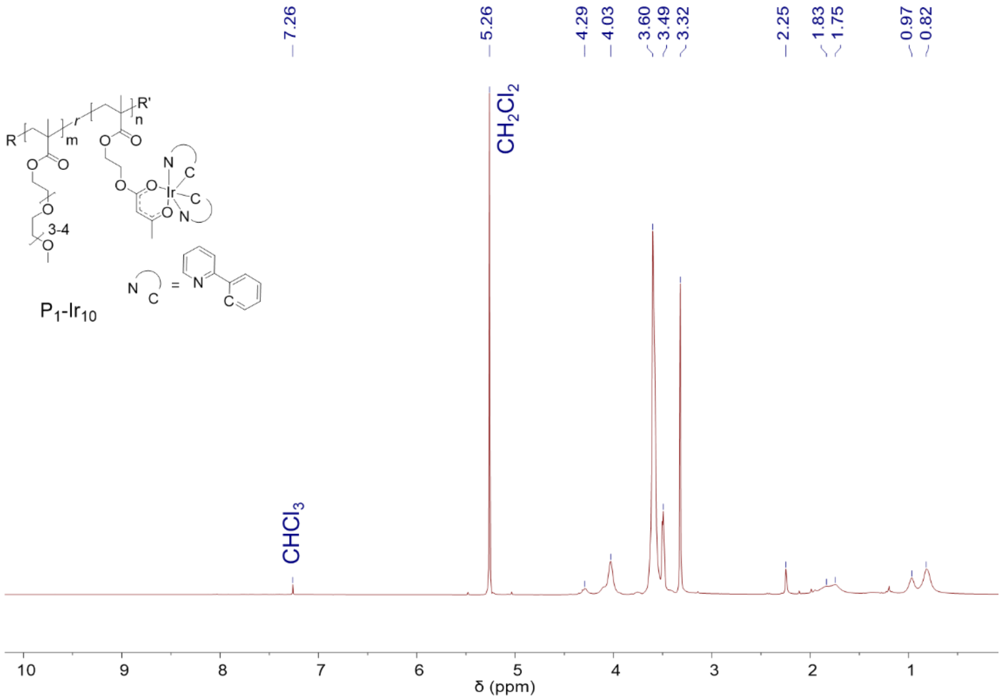

Figure S25. ^{1}H NMR spectrum of **P1-Ir40**.

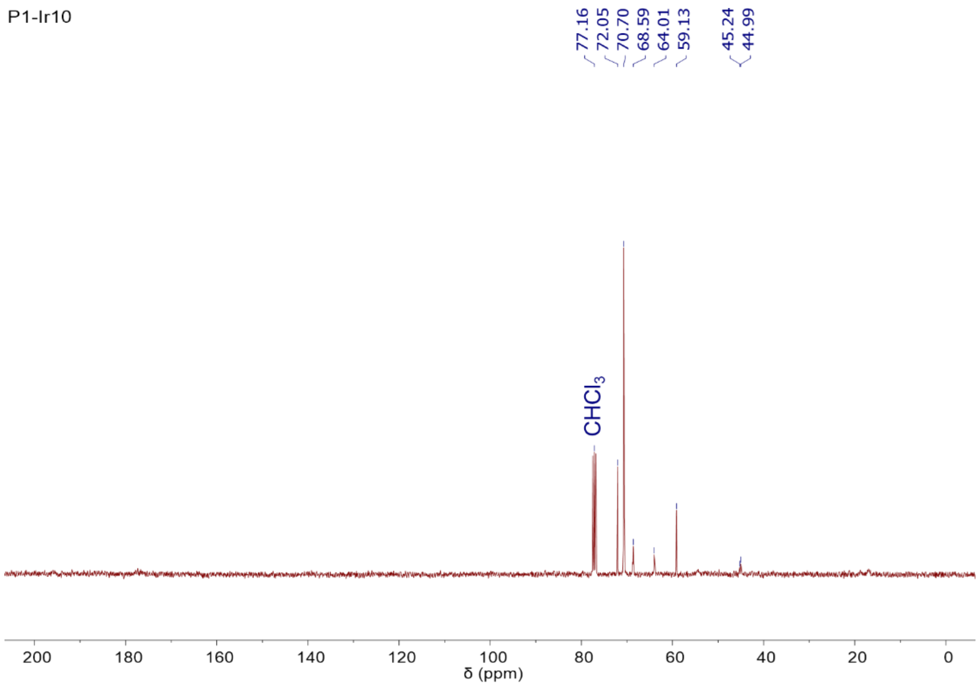

Figure S26. ^{13}C NMR spectrum of **P1-Ir40**.

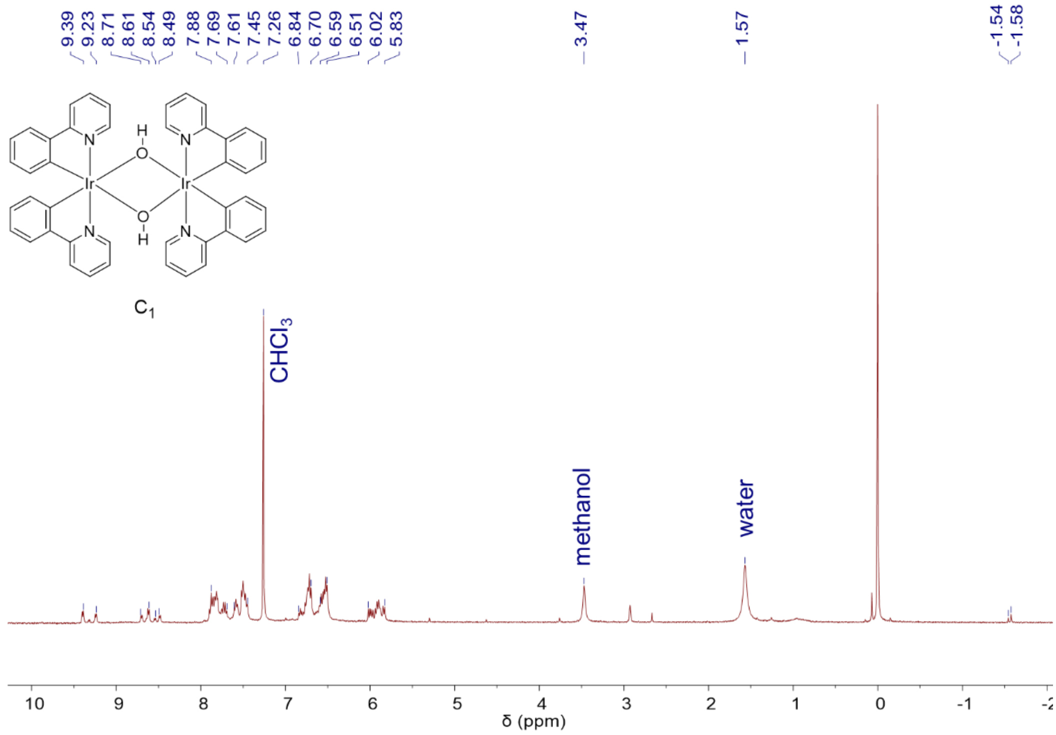

Figure S27. ^{1}H NMR spectrum of **C1**.

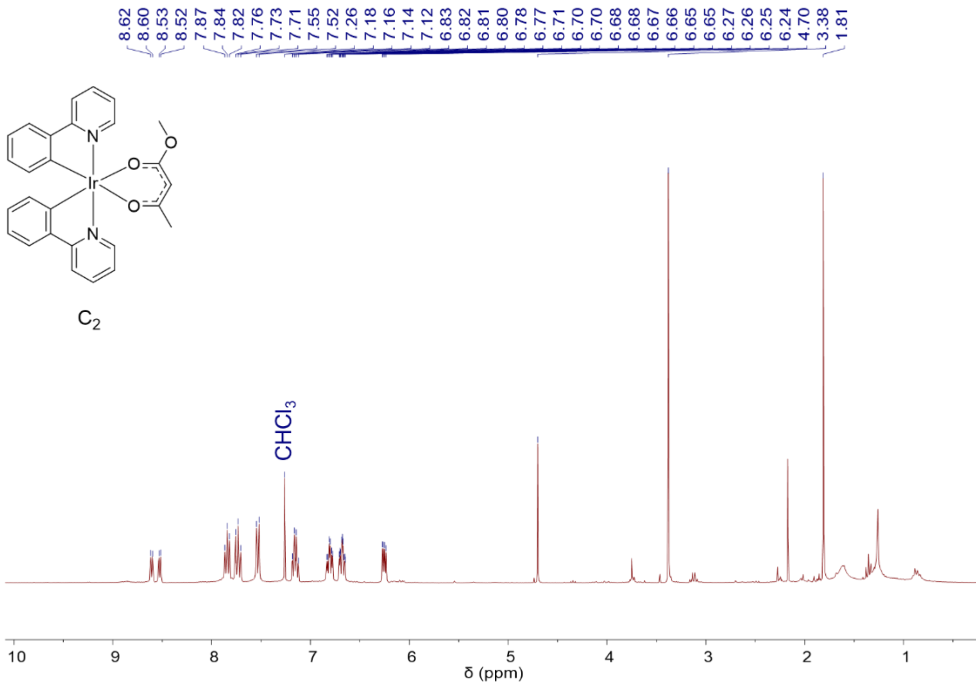

Figure S28. ^{1}H NMR spectrum of **C2**.

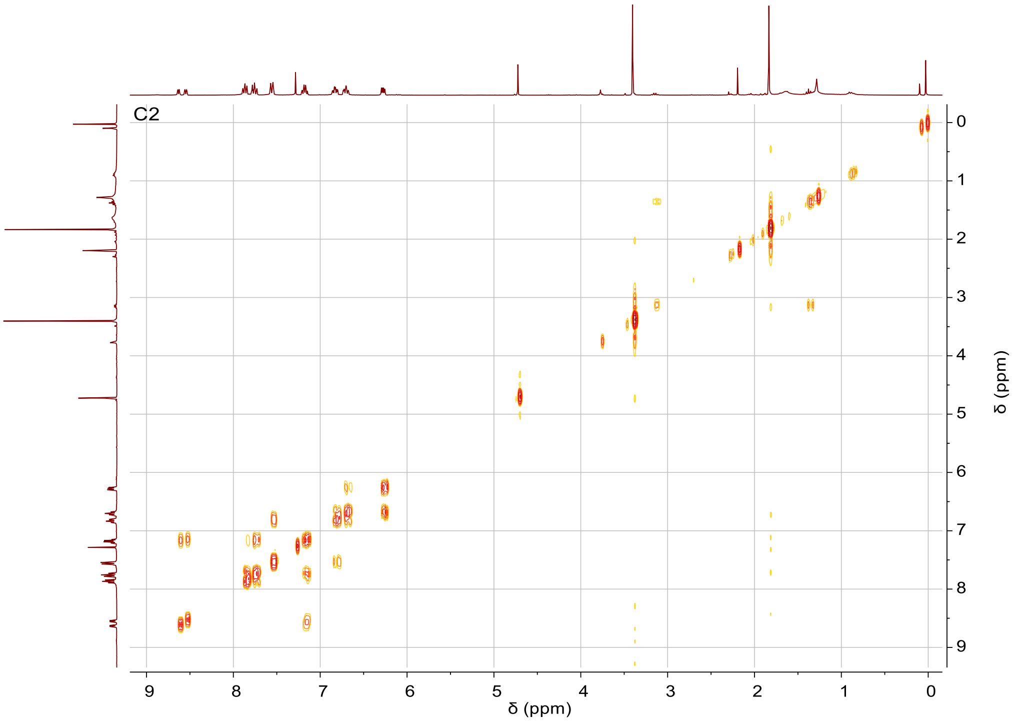

Figure S29. ^{1}H COSY spectrum of **C2**.

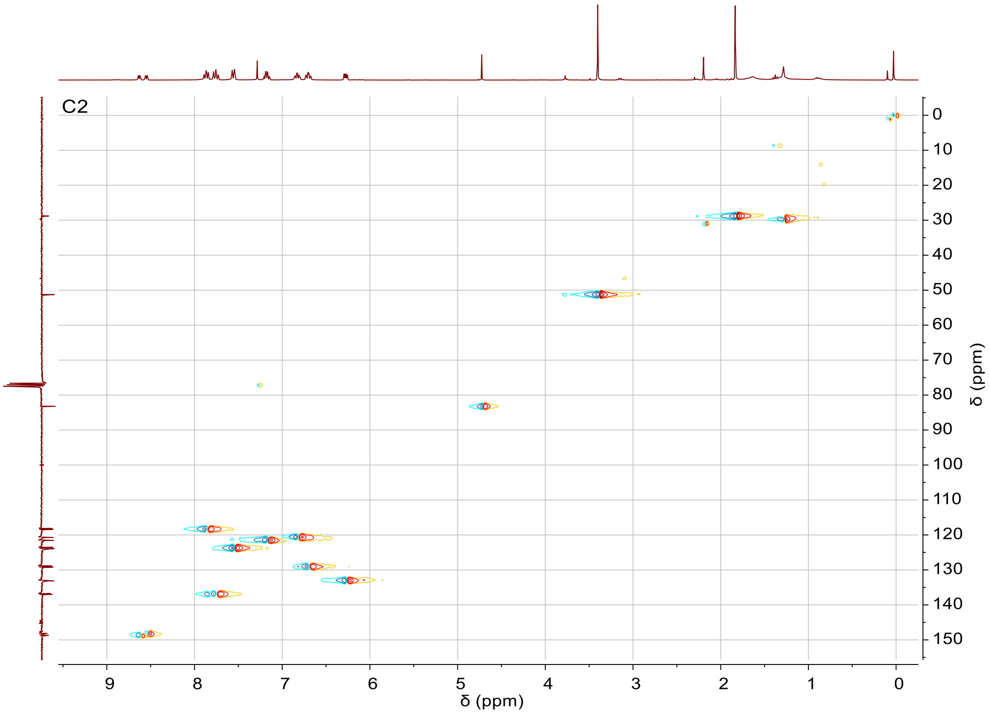

Figure S30. ^{1}H-^{13}C HSQC spectrum of **C2**.

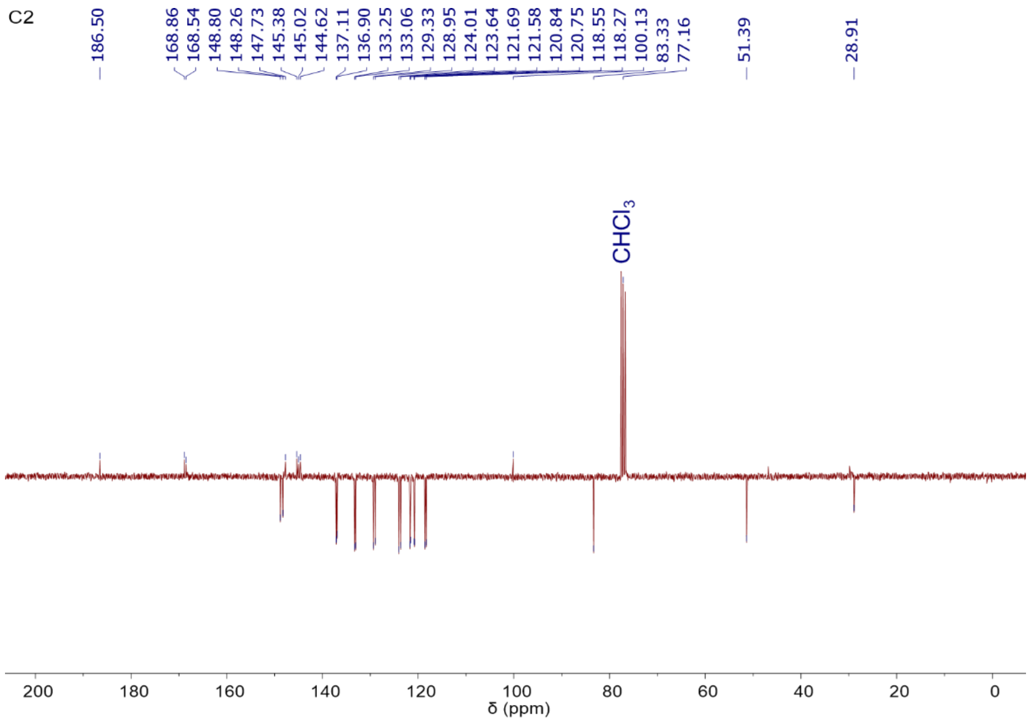

Figure S31. ^{13}C DEPT spectrum of **C2**.

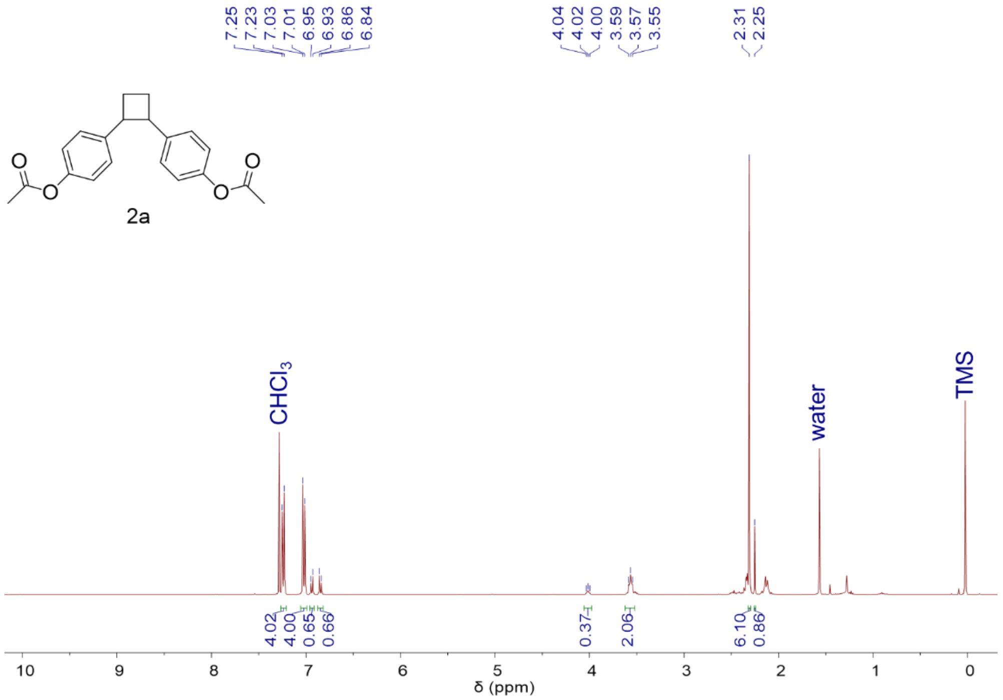

Figure S32. ^{1}H NMR spectrum of **2a.**

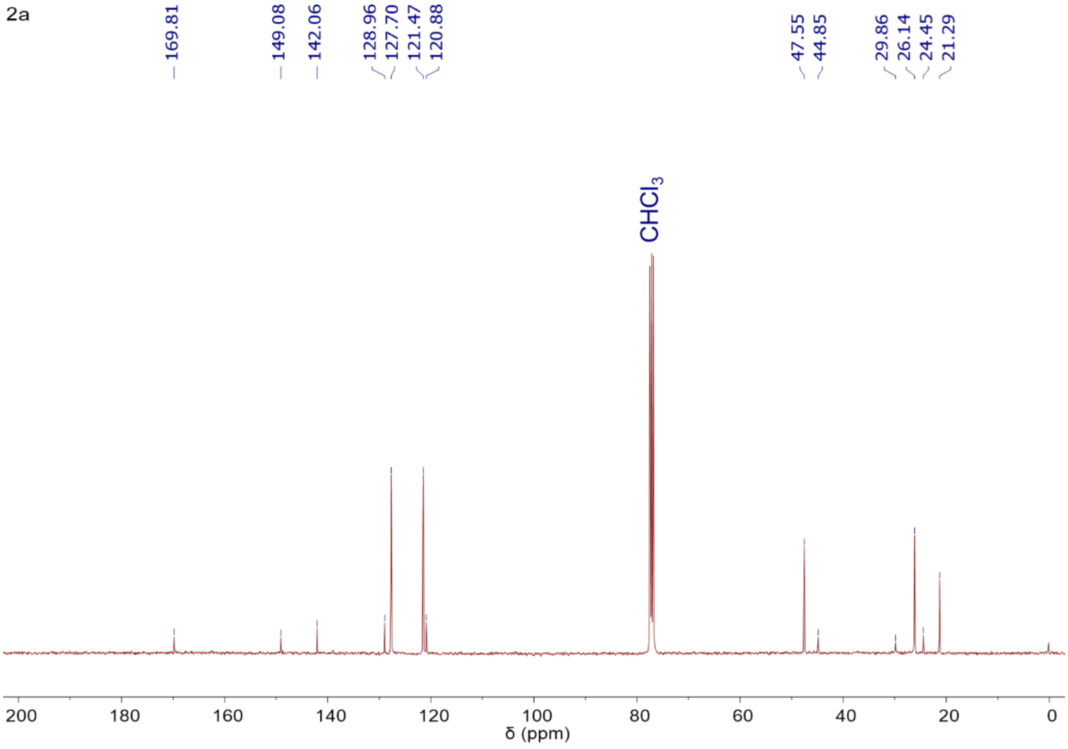

Figure S33. ^{13}C NMR spectrum of **2a.**

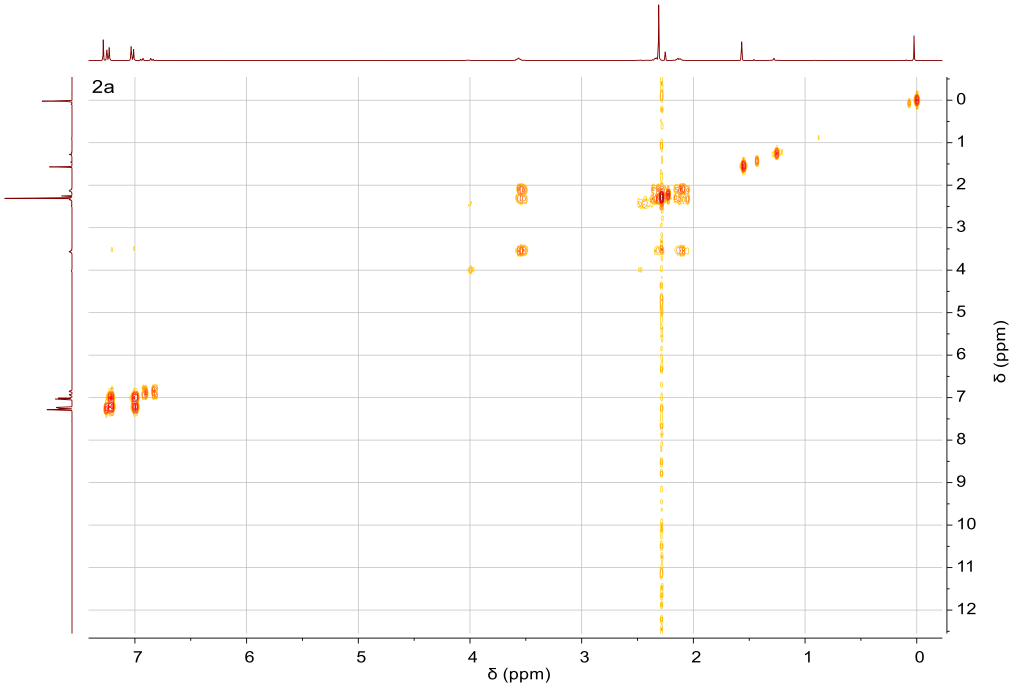

Figure S34. ¹H COSY spectrum of **2a**.

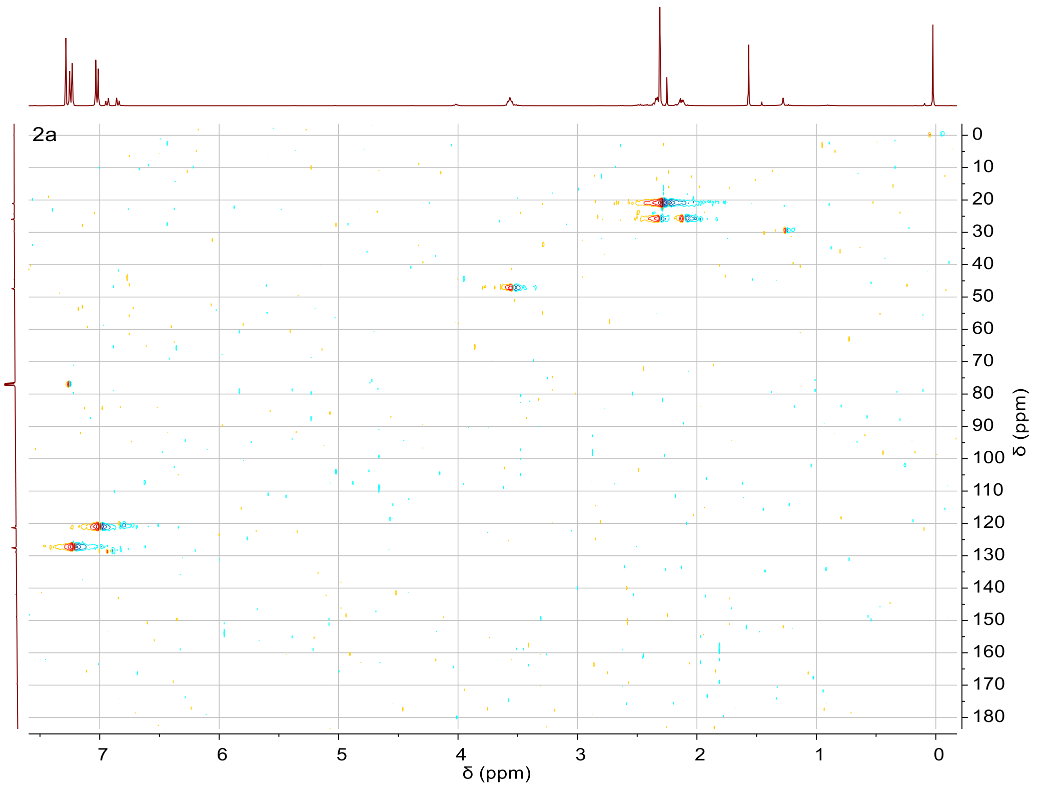

Figure S35. ¹H-¹³C HSQC spectrum of **2a.**

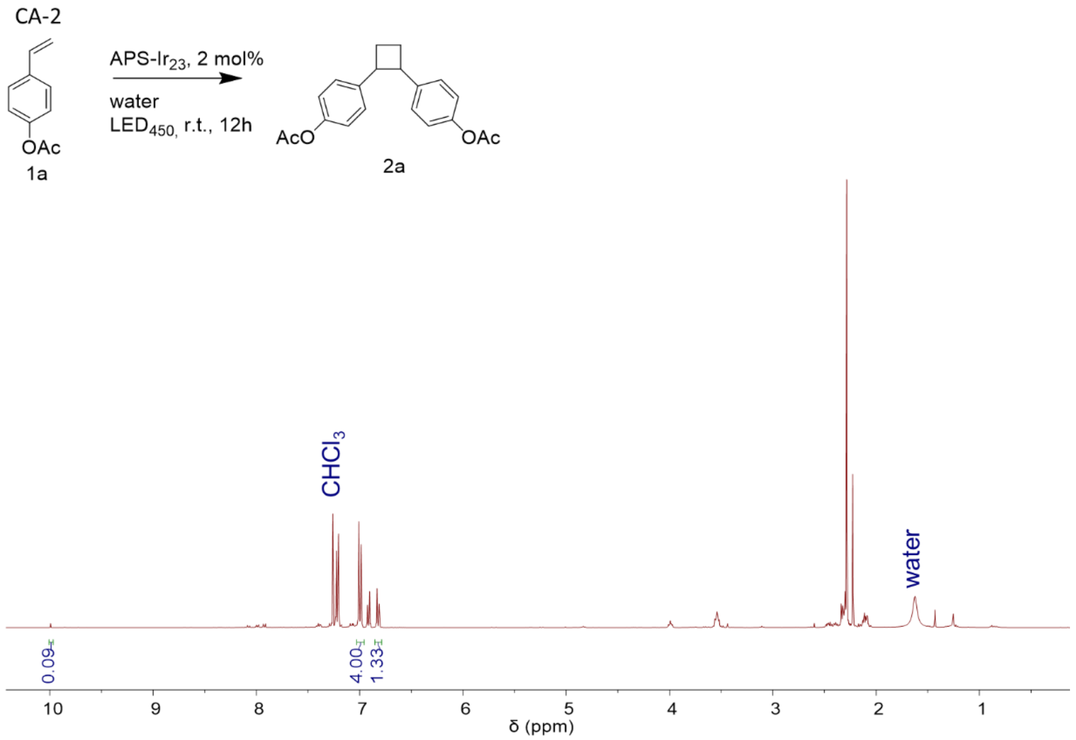

Figure S36. ^{1}H NMR spectrum of the extracted crude from reaction **CA-2**.

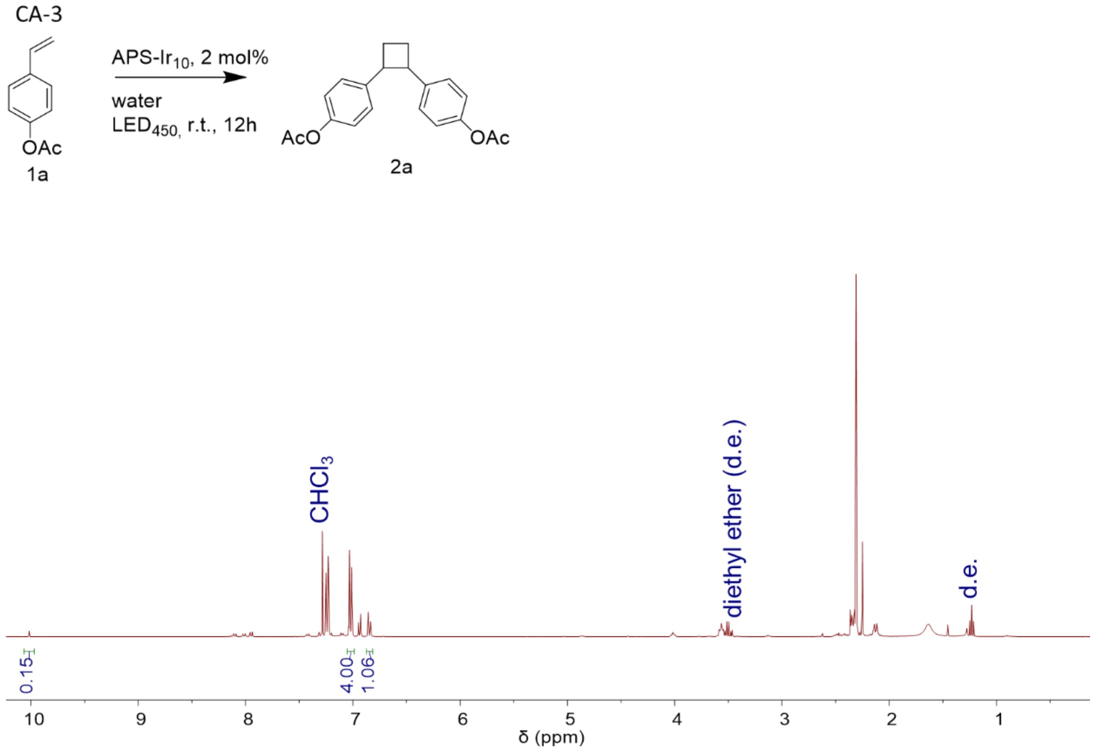

Figure S37. ^{1}H NMR spectrum of the extracted crude from reaction **CA-3**.

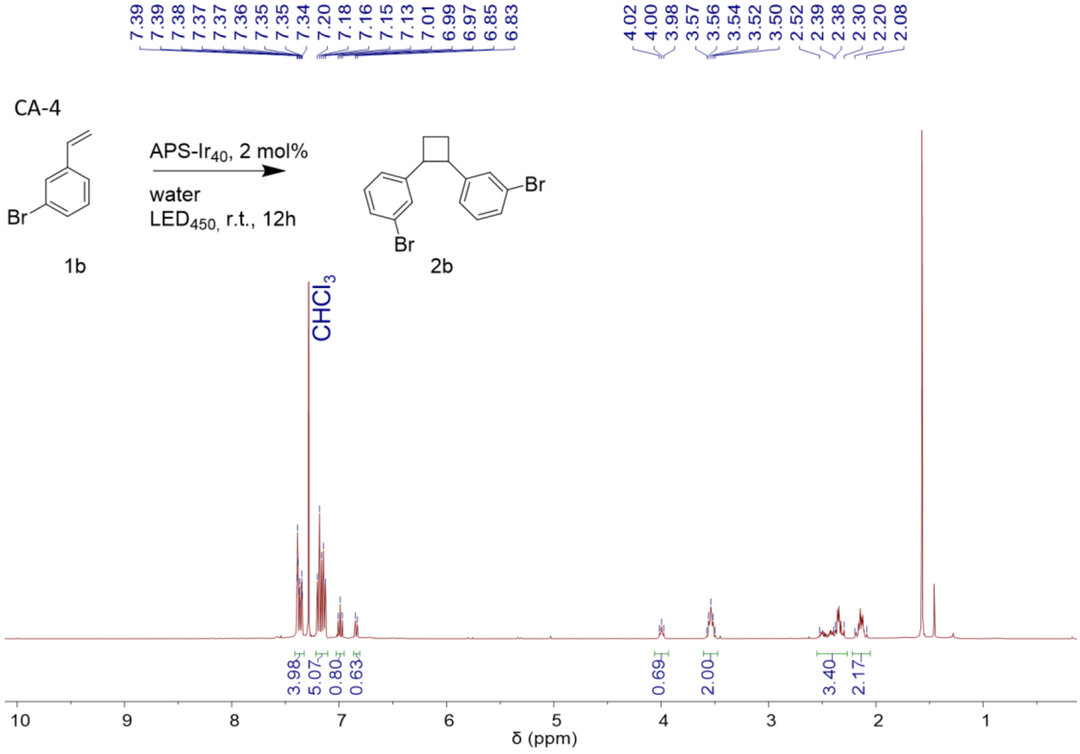

Figure S38. ^{1}H NMR spectrum of the extracted crude from reaction **CA-4**.

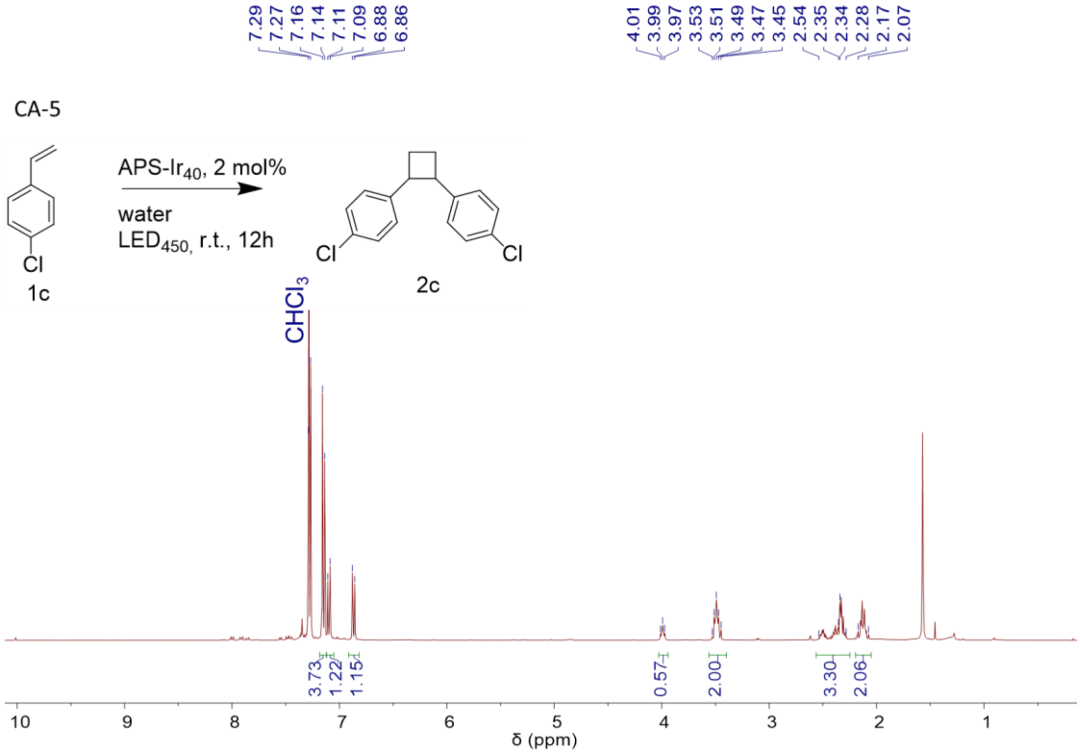

Figure S39. ^{1}H NMR spectrum of the extracted crude from reaction **CA-5**.

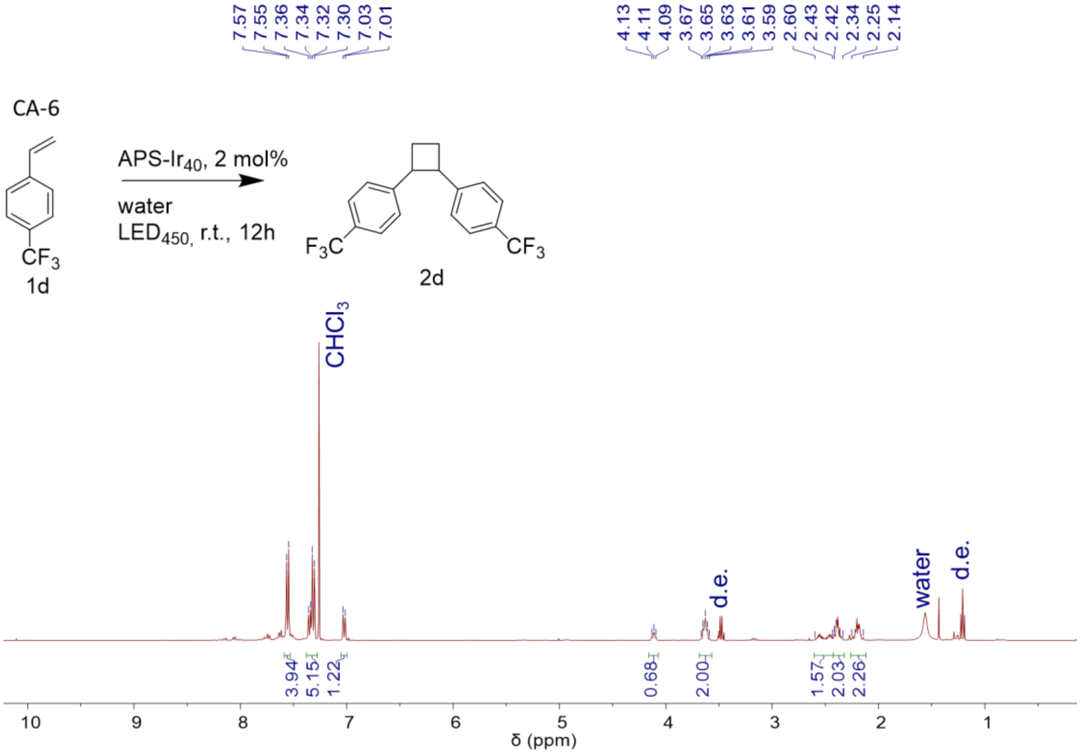

Figure S40. ^{1}H NMR spectrum of the extracted crude from reaction **CA-6**.

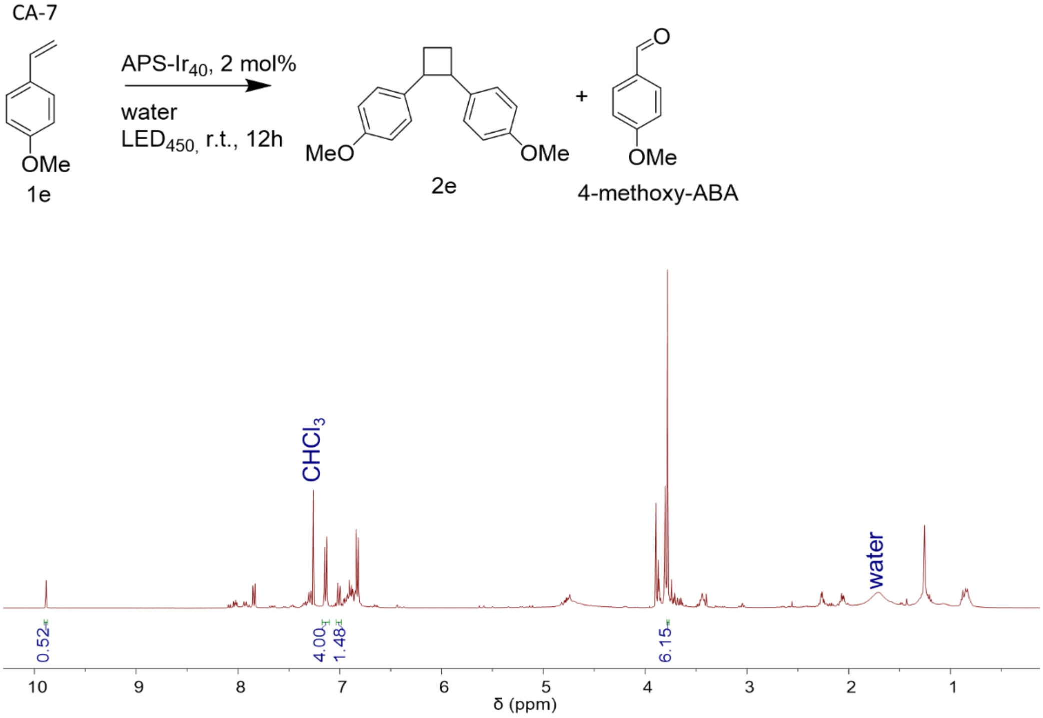

Figure S41. ^{1}H NMR spectrum of the extracted crude from reaction **CA-7.**

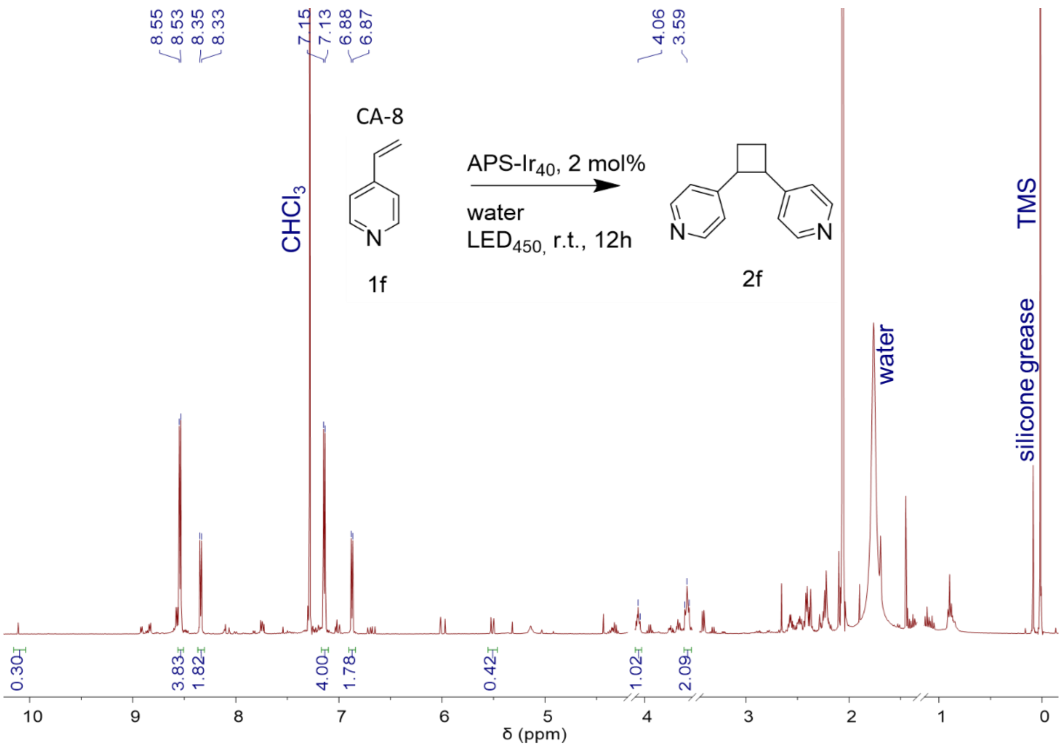

Figure S42. ^{1}H NMR spectrum of the extracted crude from reaction **CA-8**.

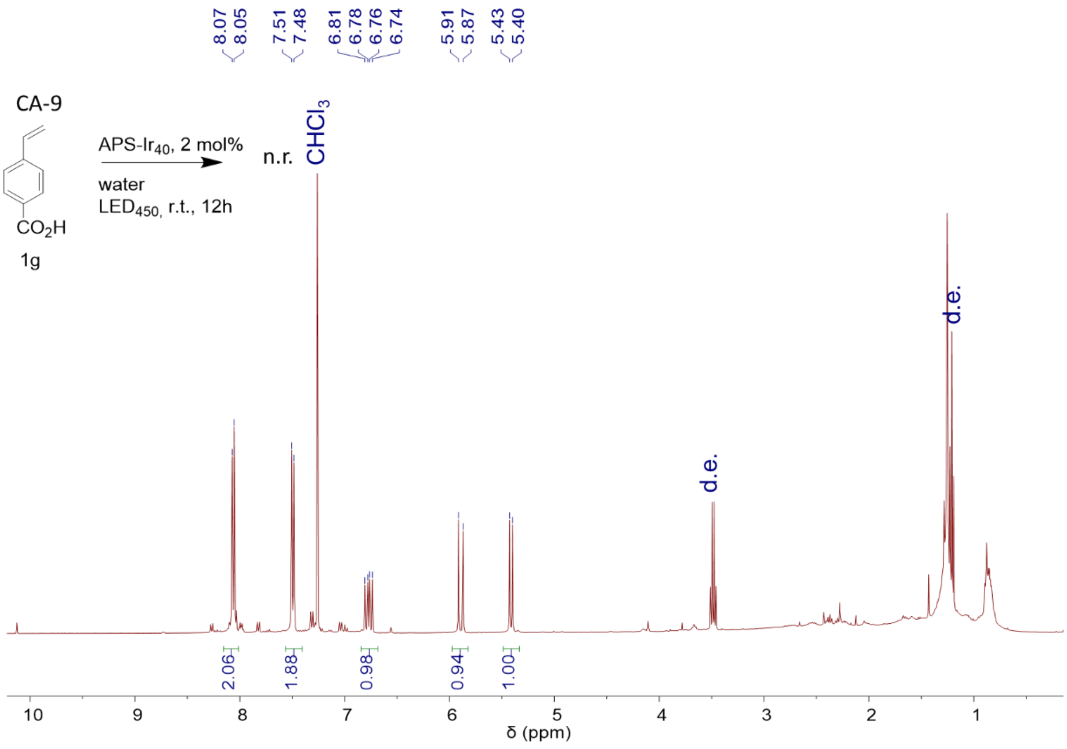

Figure S43. ^{1}H NMR spectrum of the extracted crude from reaction **CA-9**.

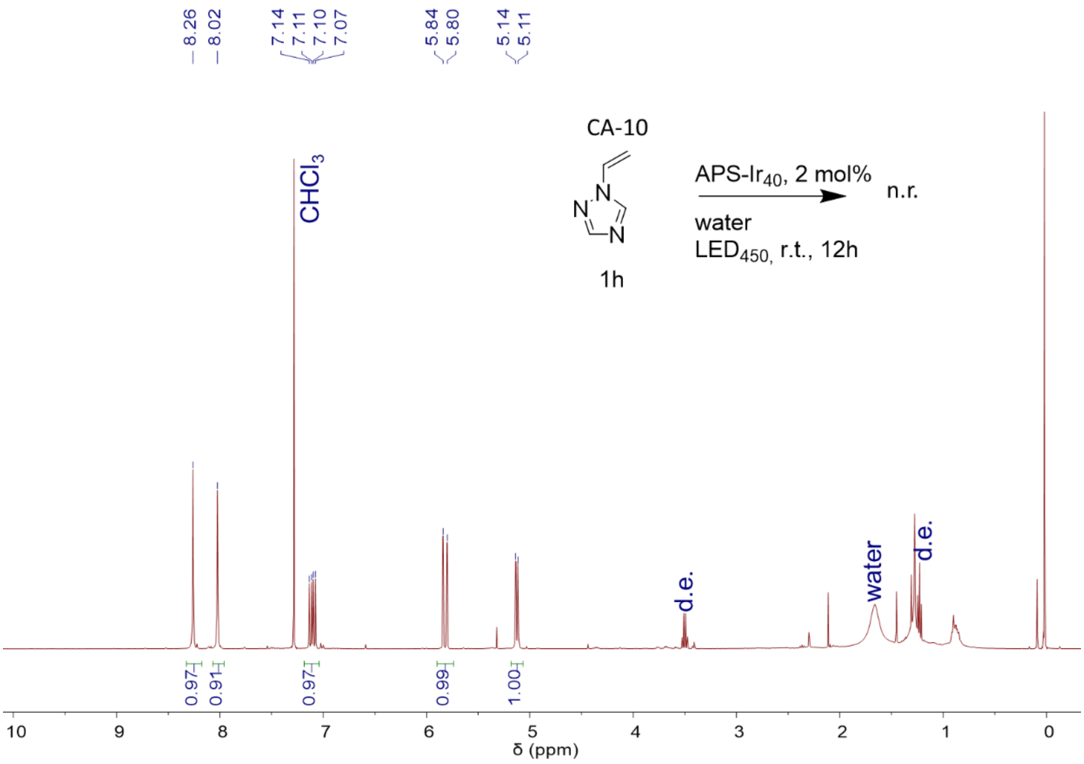

Figure S44. ^{1}H NMR spectrum of the extracted crude from reaction **CA-10**.

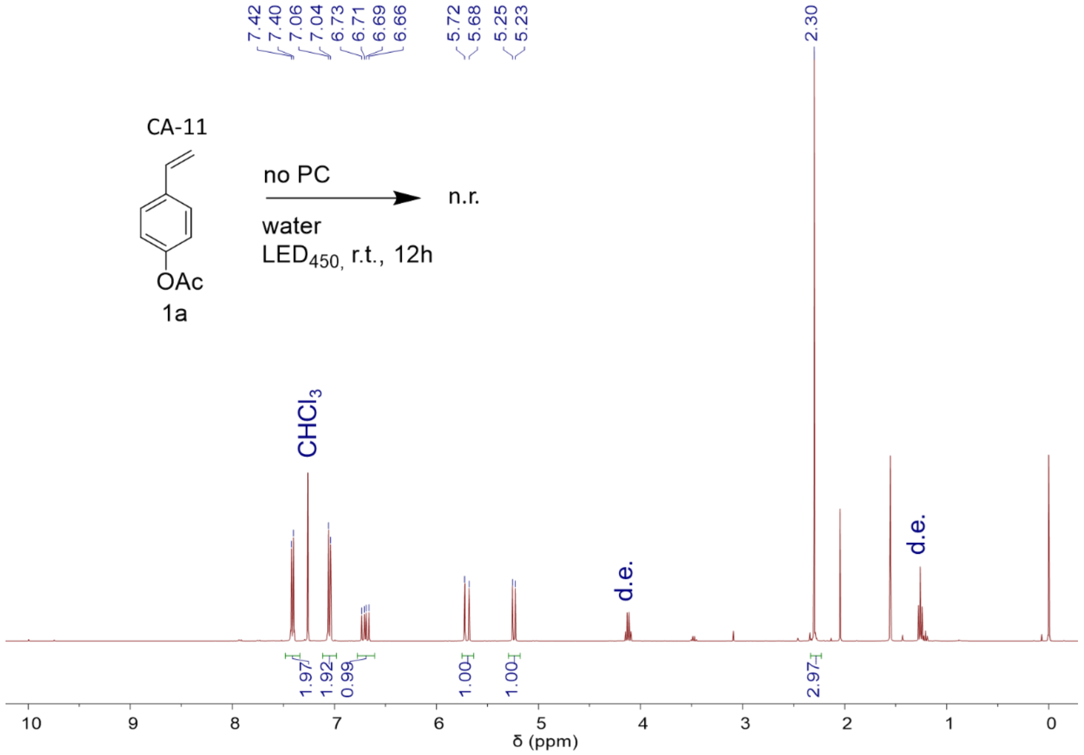

Figure S45. ^{1}H NMR spectrum of the extracted crude from reaction **CA-11**.

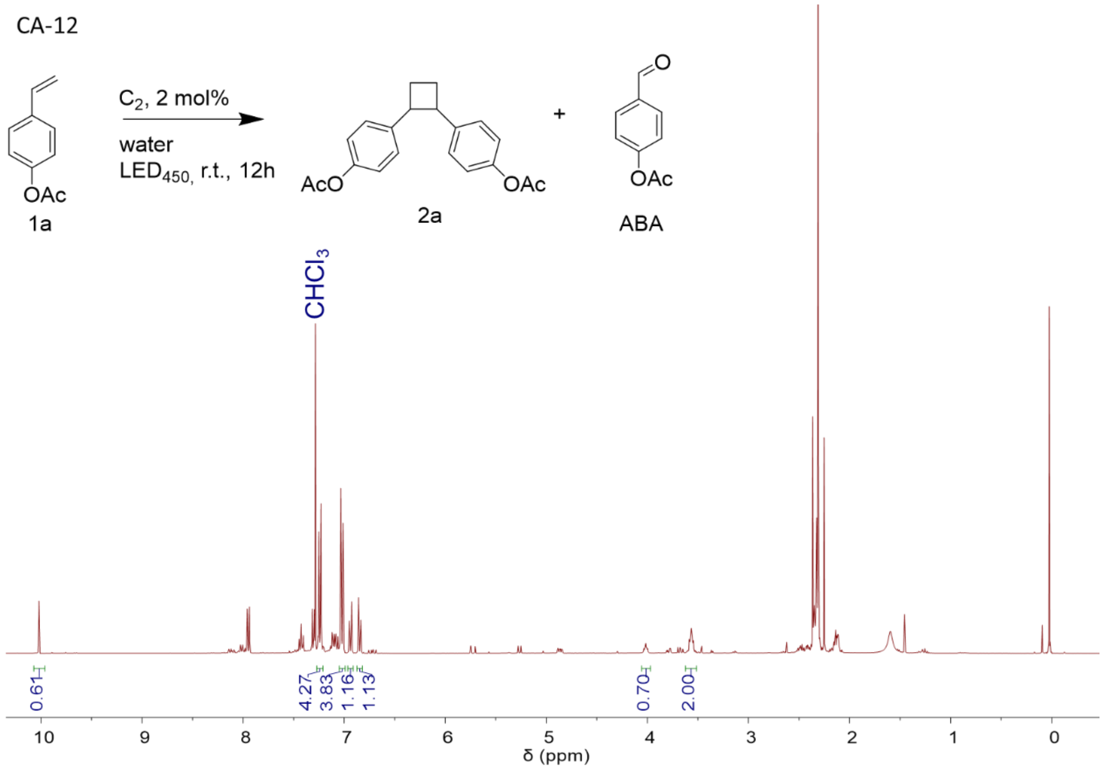

Figure S46. ^{1}H NMR spectrum of the extracted crude from reaction **CA-12**.

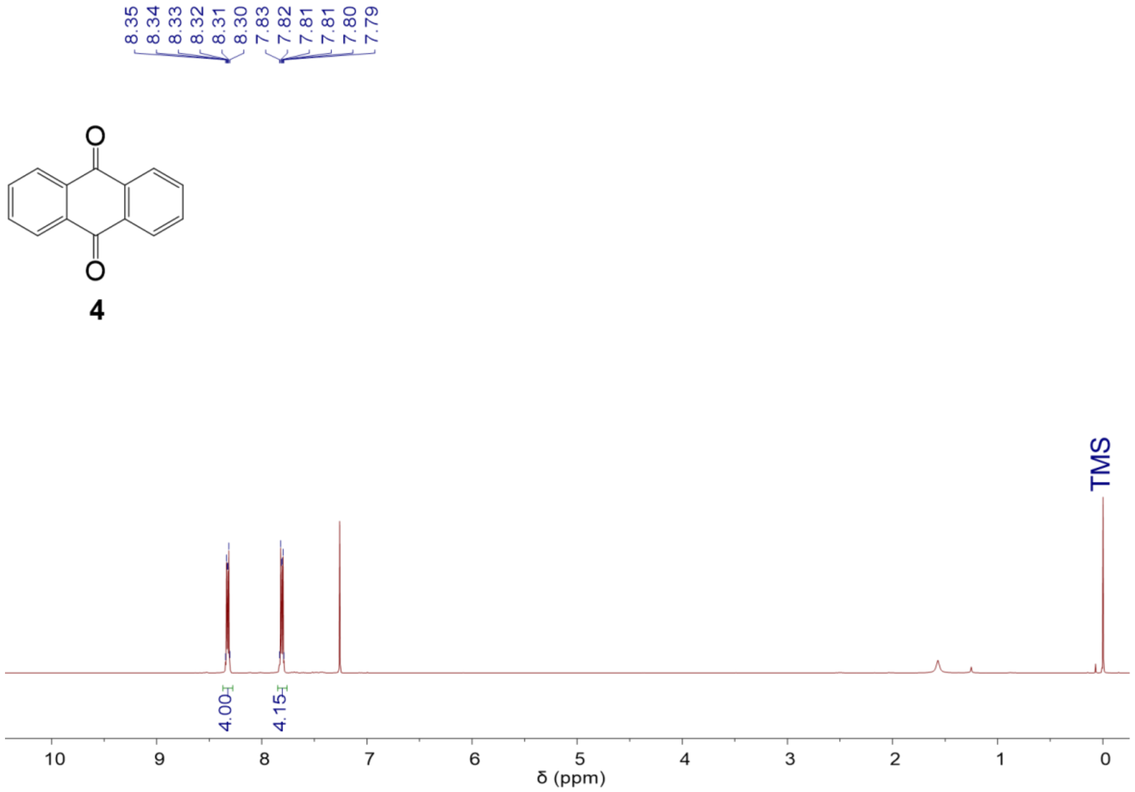

Figure S47. ^{1}H NMR spectrum of **4**.

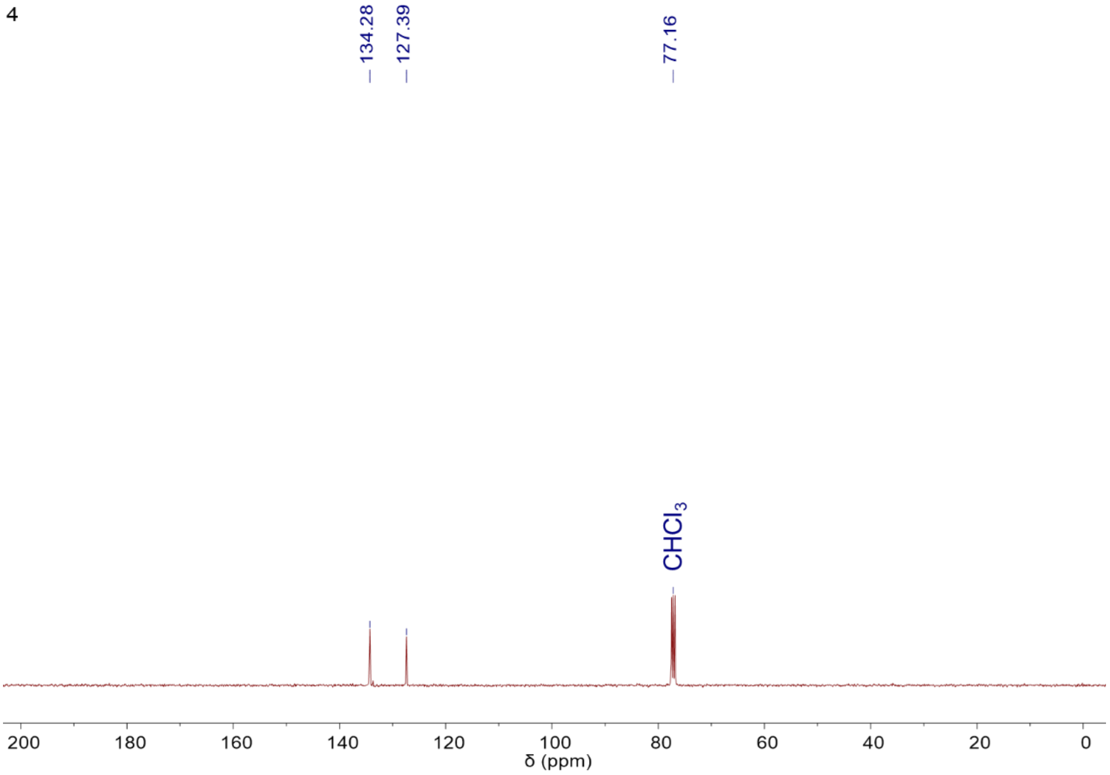

Figure S48. ^{13}C NMR spectrum of **4**.

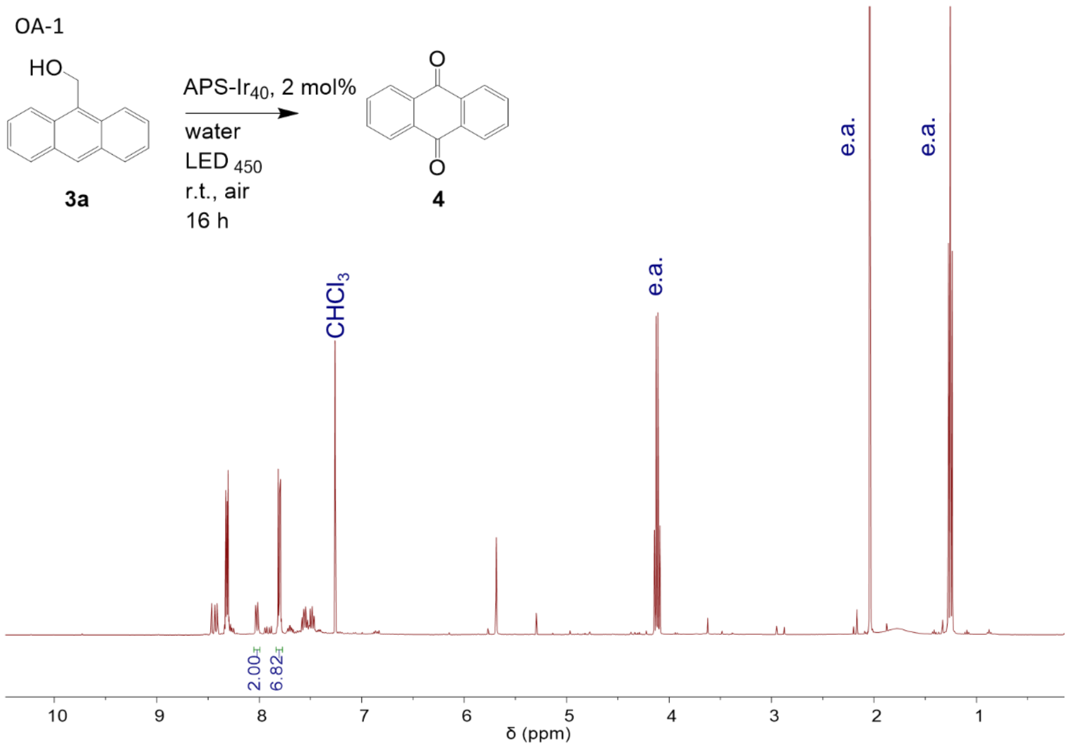

Figure S49. ^{1}H NMR spectrum of the extracted crude from reaction **OA-1** (e.a. = ethyl acetate).

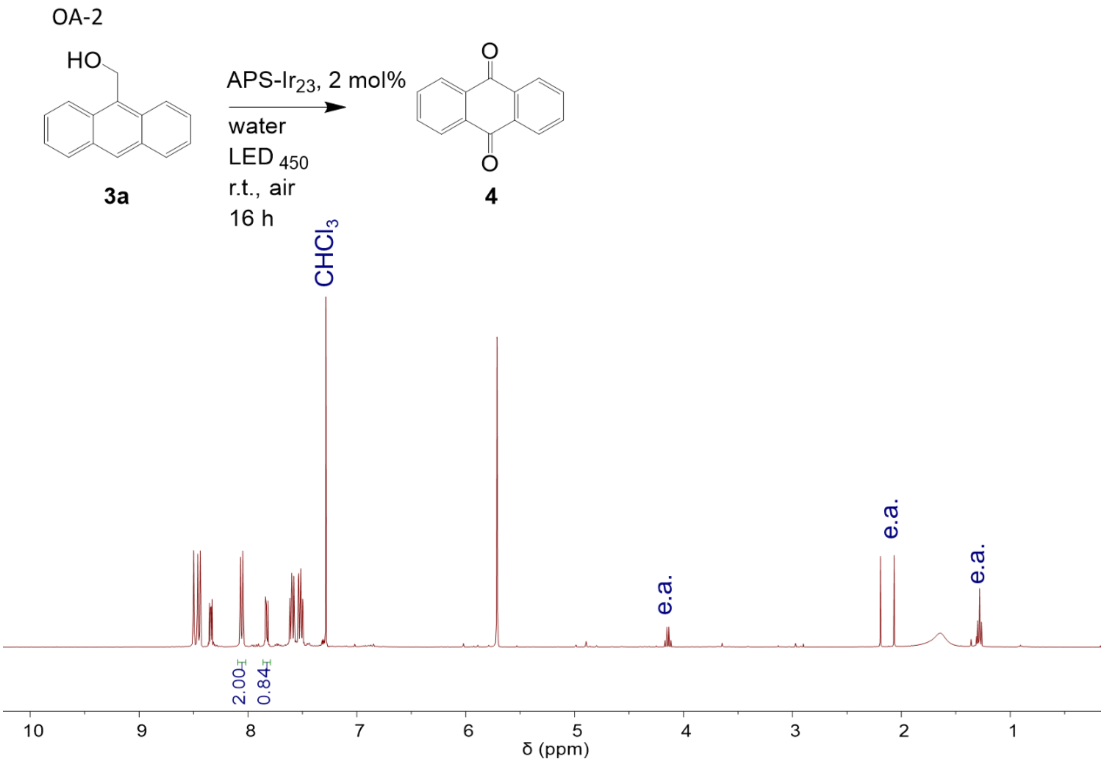

Figure S50. ^{1}H NMR spectrum of the extracted crude from reaction **OA-2** (e.a. = ethyl acetate).

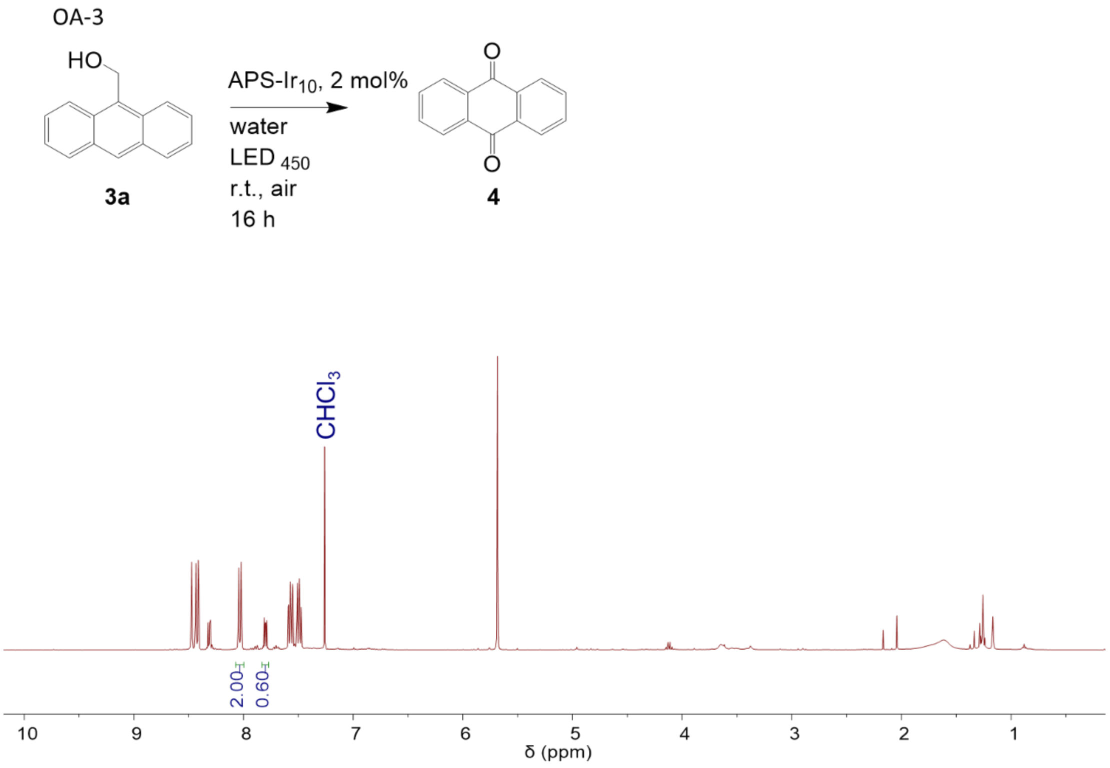

Figure S51. ^{1}H NMR spectrum of the extracted crude from reaction **OA-3.**

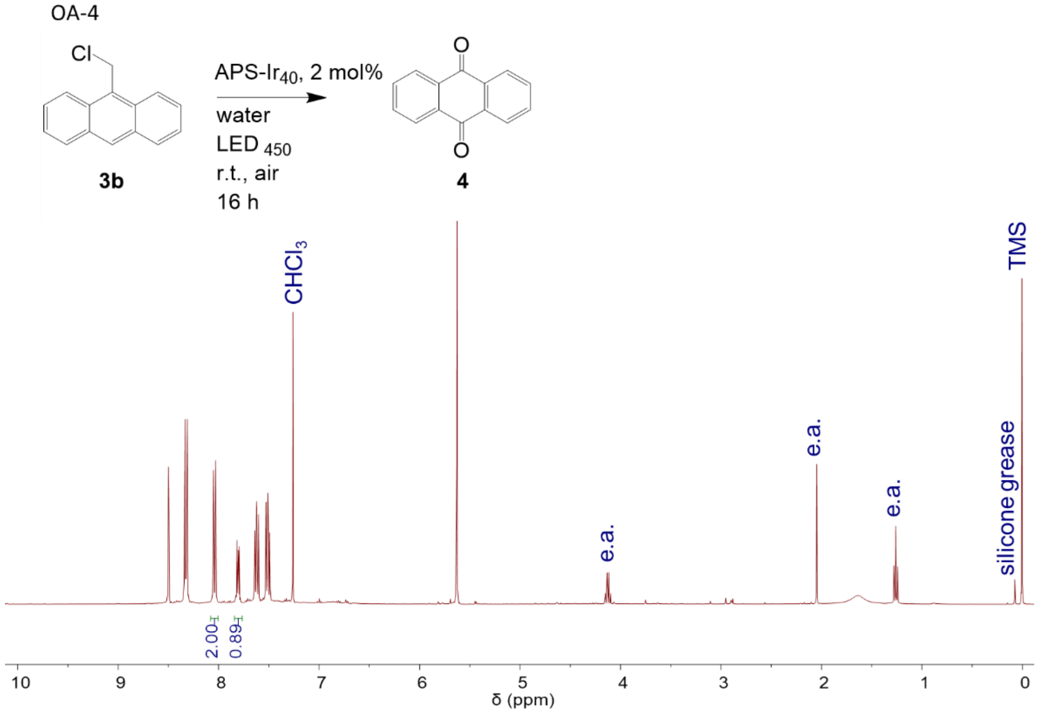

Figure S52. ^{1}H NMR spectrum of the extracted crude from reaction **OA-4** (e.a. = ethyl acetate).

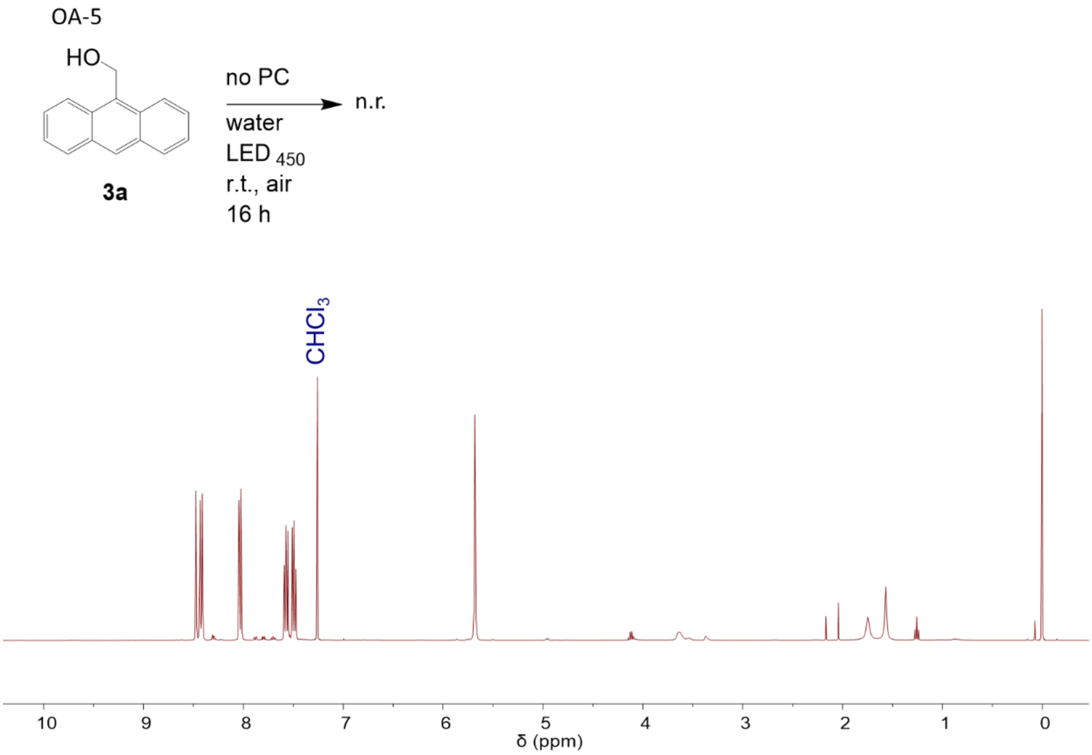

Figure S53. ^{1}H NMR spectrum of the extracted crude from reaction **OA-5.**

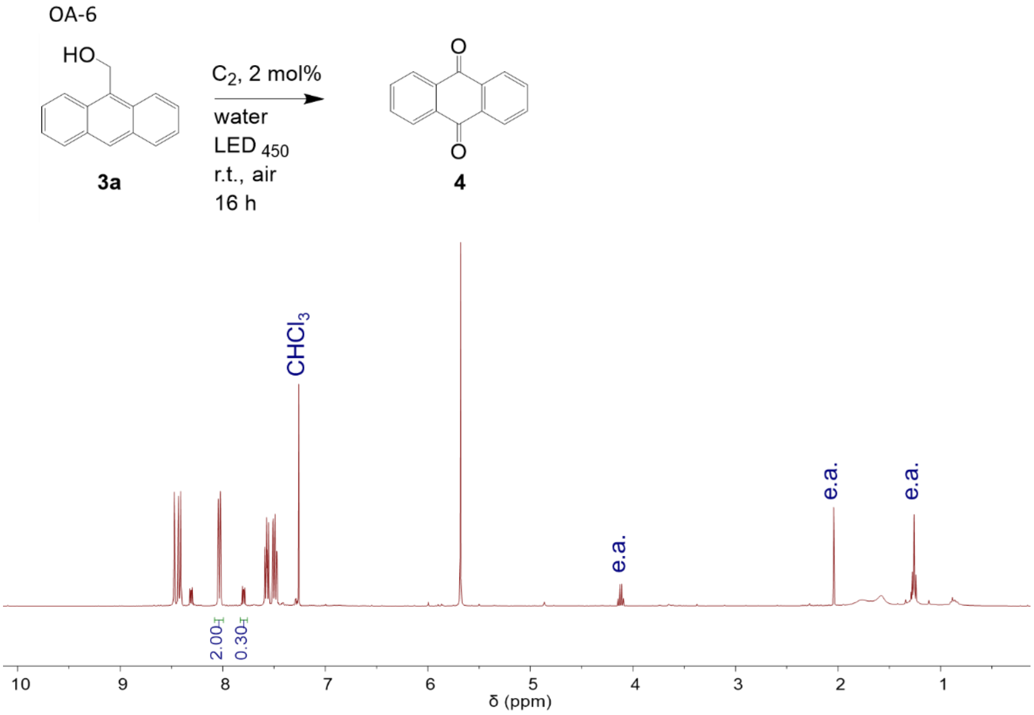

Figure S54. ^{1}H NMR spectrum of the extracted crude from reaction **OA-6** (e.a. = ethyl acetate).

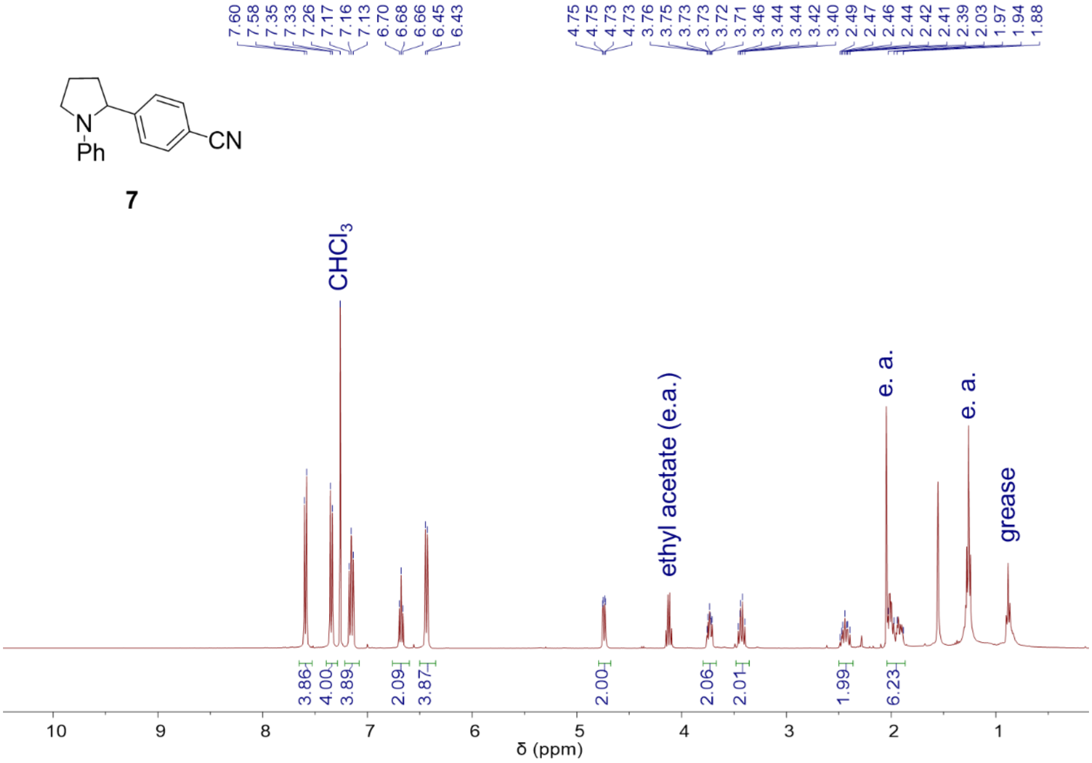

Figure S55. ^{1}H NMR spectrum of **7.**

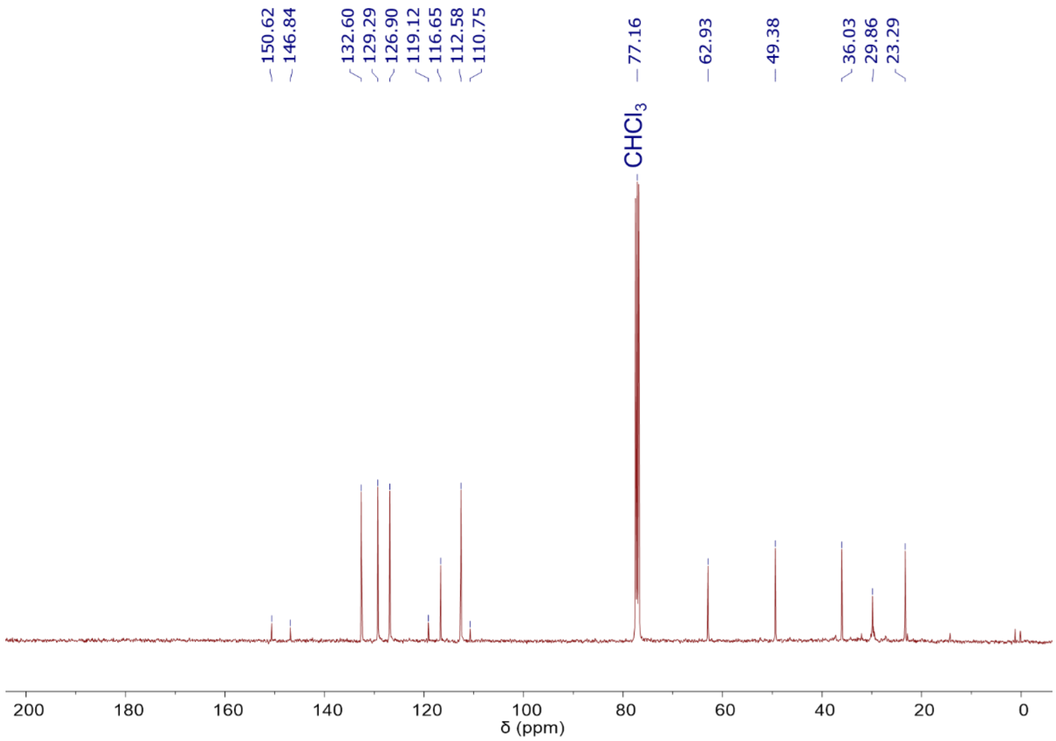

Figure S56. ^{13}C NMR spectrum of **7.**

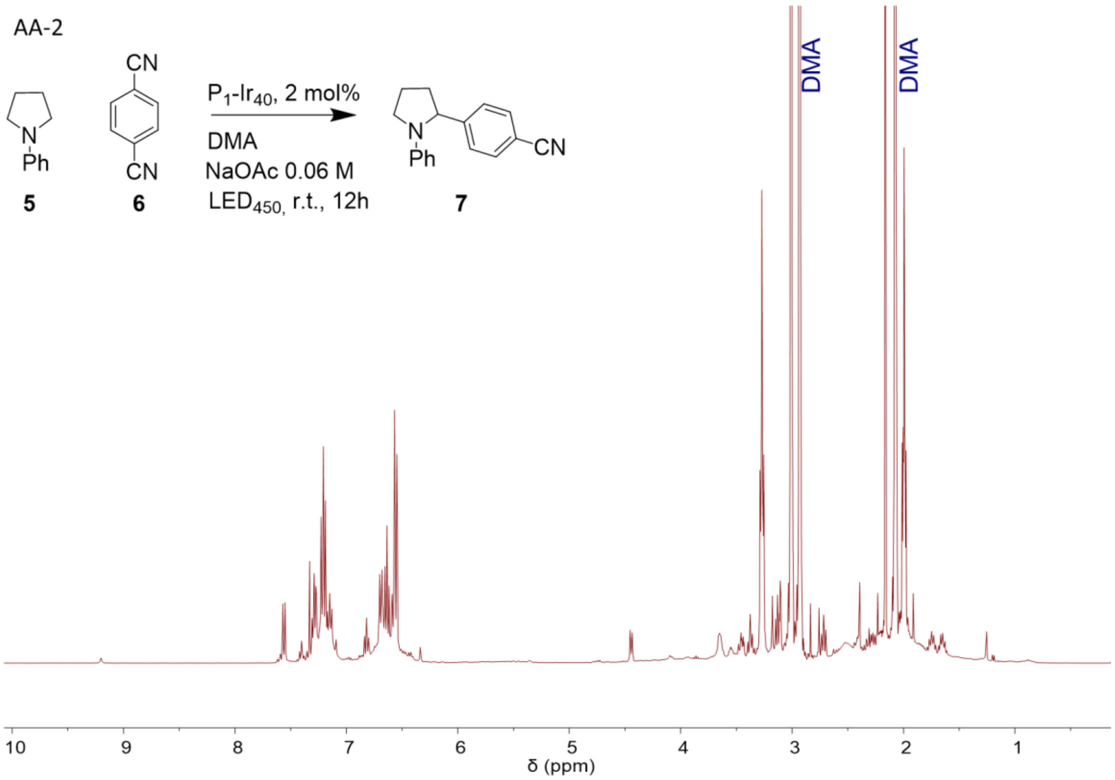

Figure S57. ^{1}H NMR spectrum of the extracted crude from reaction **AA-2** (DMA = dimethylacetamide).

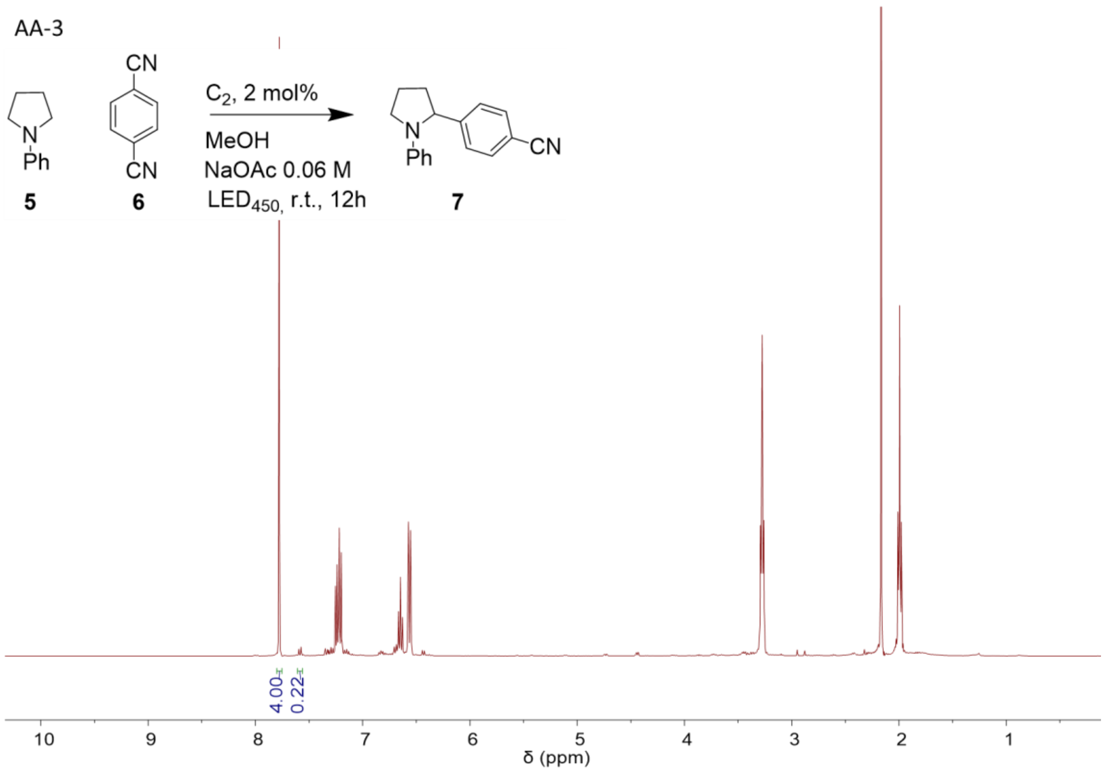

Figure S58. ^{1}H NMR spectrum of the extracted crude from reaction **AA-3.**

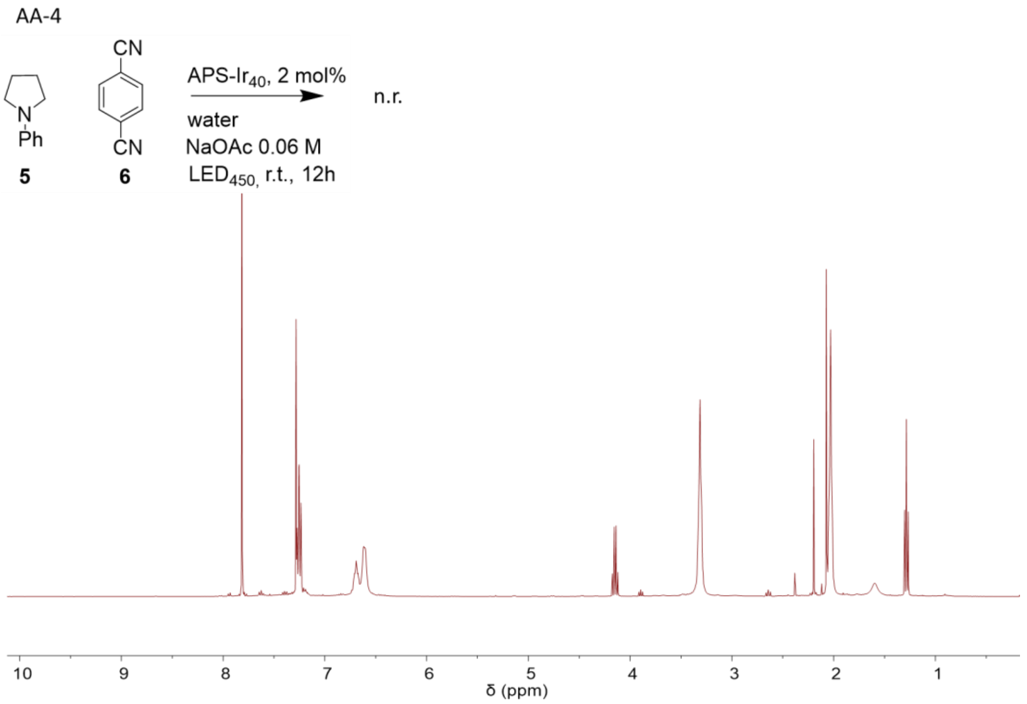

Figure S59. ^{1}H NMR spectrum of the extracted crude from reaction **AA-4.**

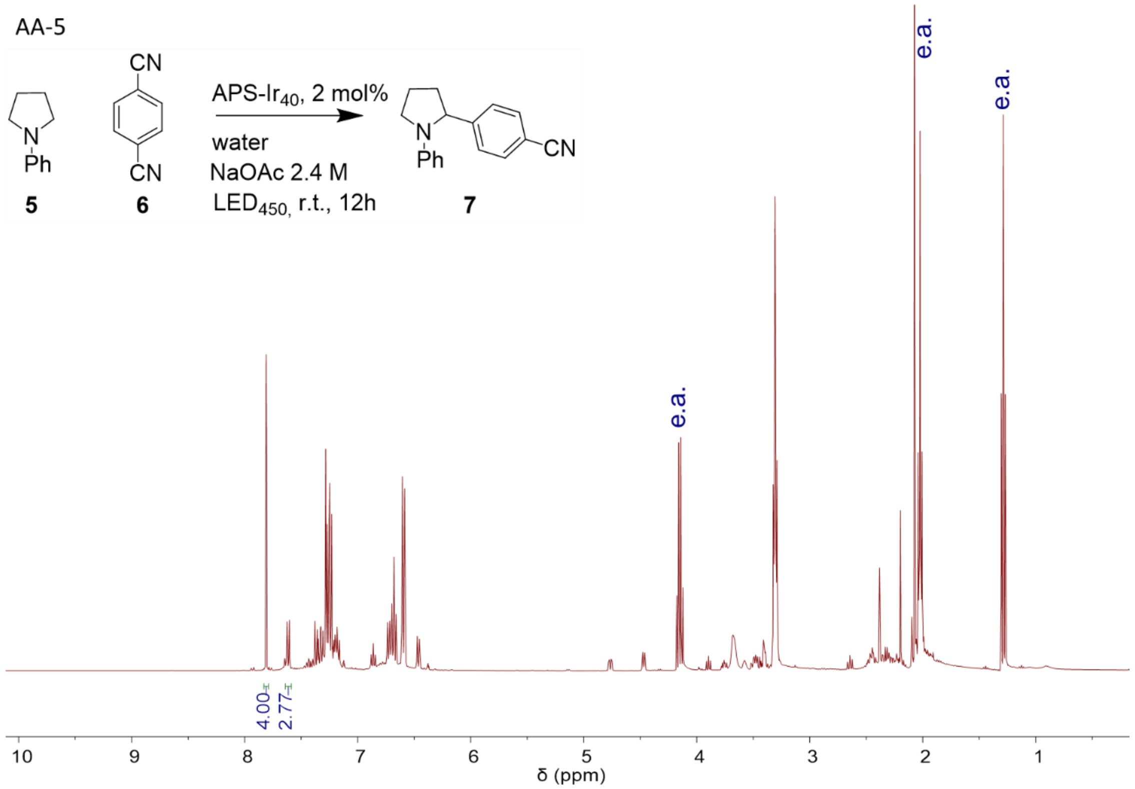

Figure S60. ^{1}H NMR spectrum of the extracted crude from reaction **AA-5** (e.a. = ethyl acetate).

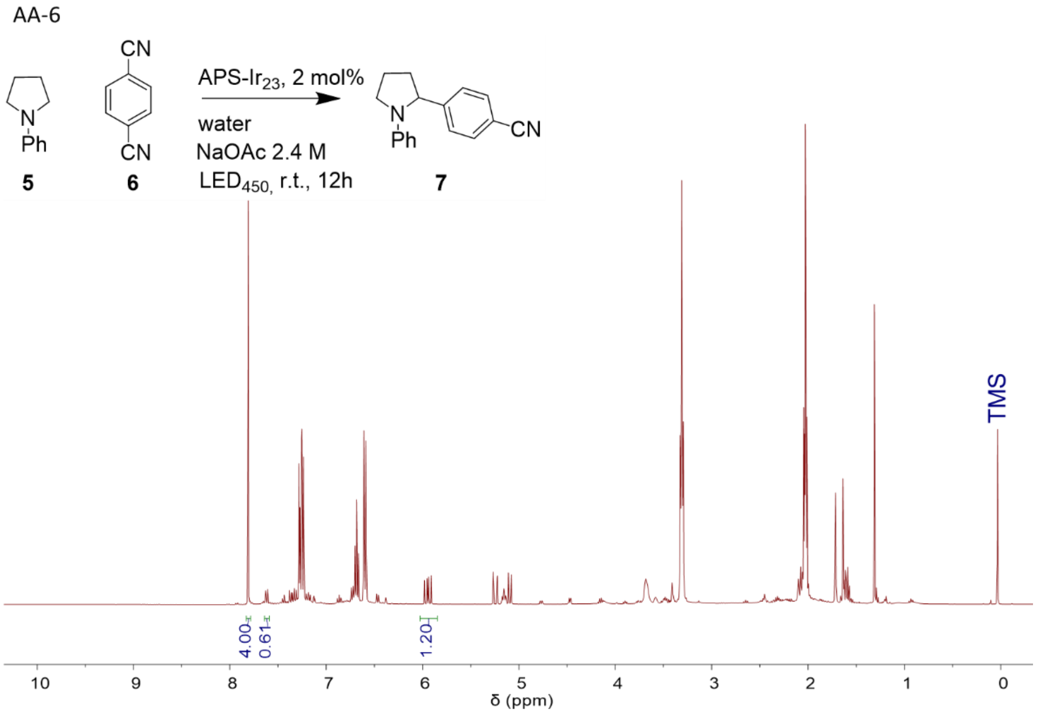

Figure S61. ^{1}H NMR spectrum of the extracted crude from reaction **AA-6.**

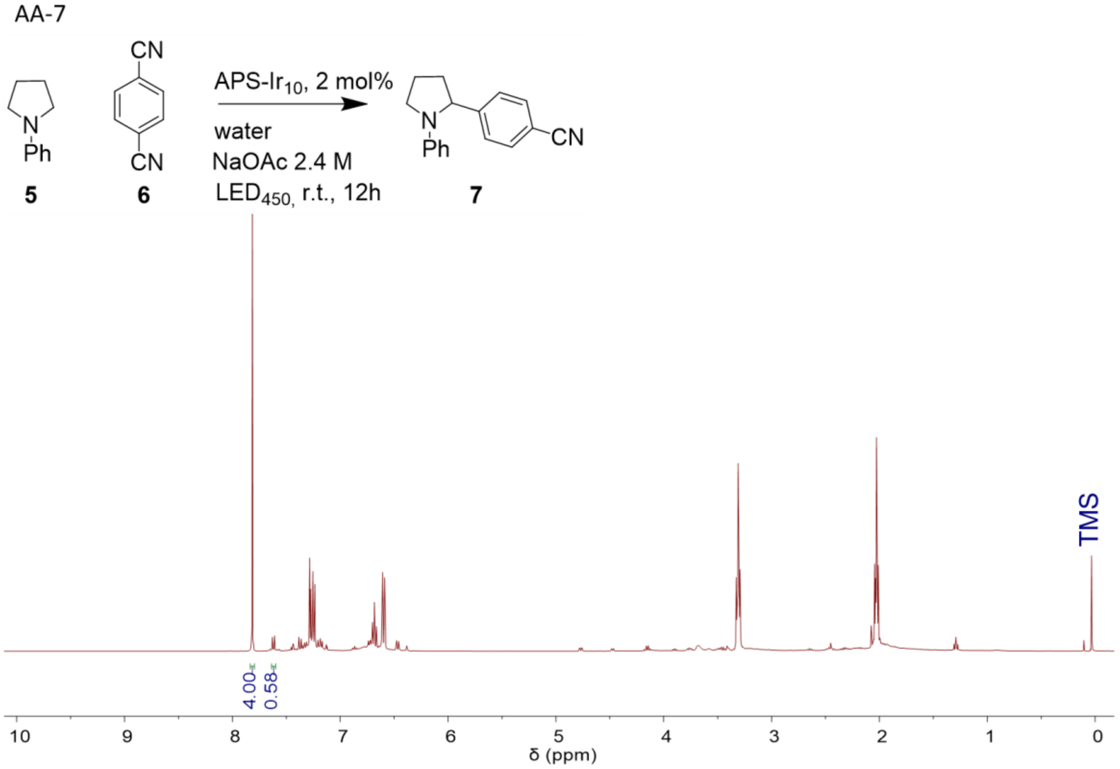

Figure S62. ^{1}H NMR spectrum of the extracted crude from reaction **AA-7.**

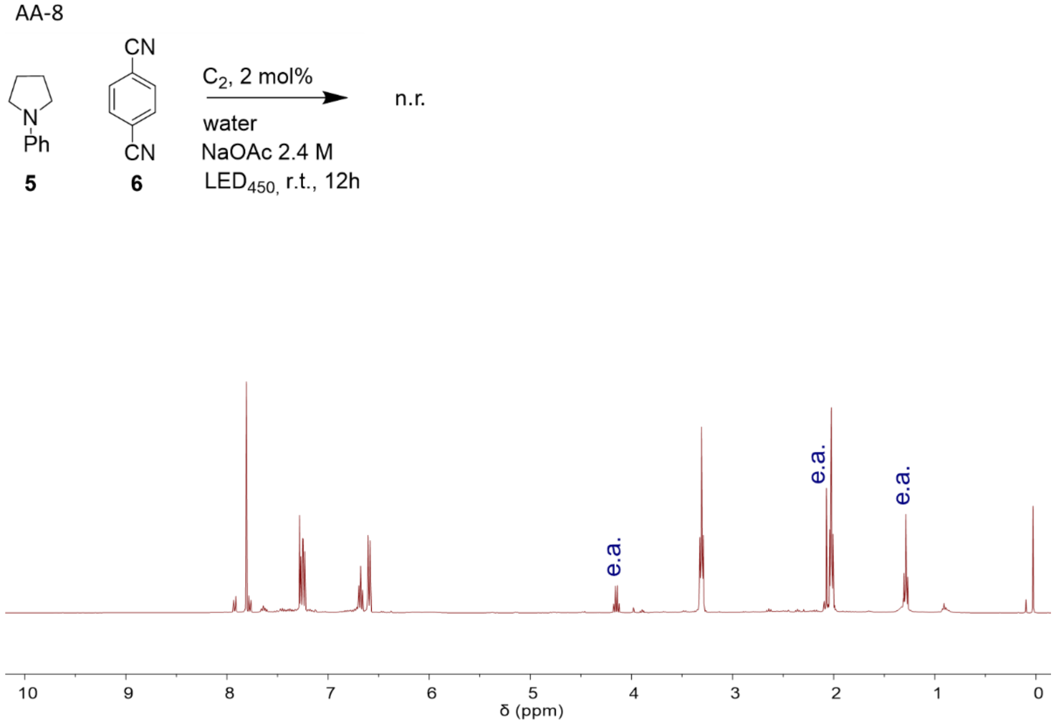

Figure S63. ^{1}H NMR spectrum of the extracted crude from reaction **AA-8** (e.a. = ethyl acetate).

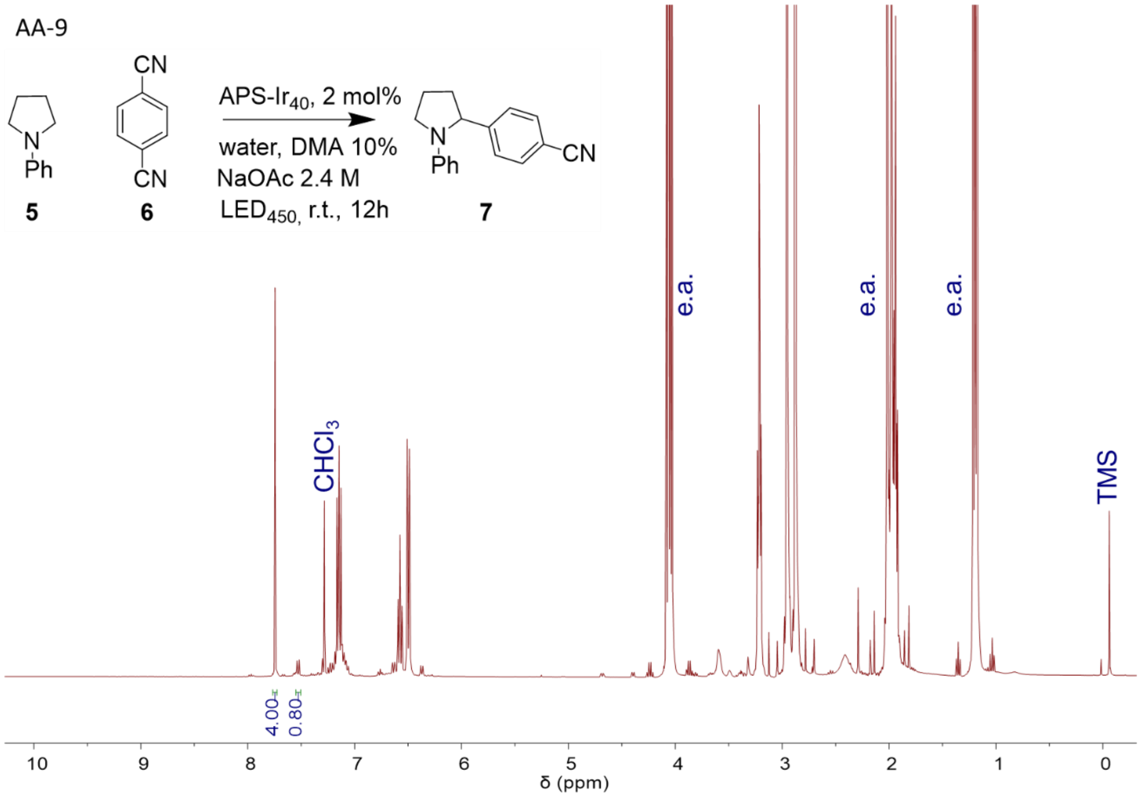

Figure S64. ^{1}H NMR spectrum of the extracted crude from reaction **AA-9** (e.a. = ethyl acetate).

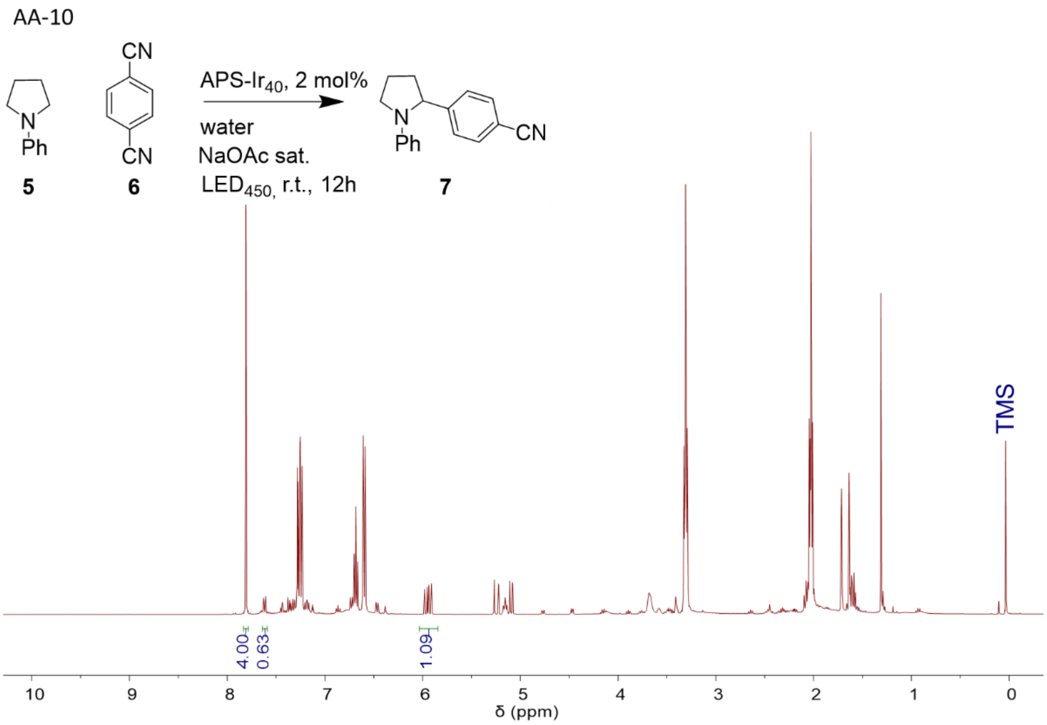

Figure S65. ^{1}H NMR spectrum of the extracted crude from reaction **AA-10.**

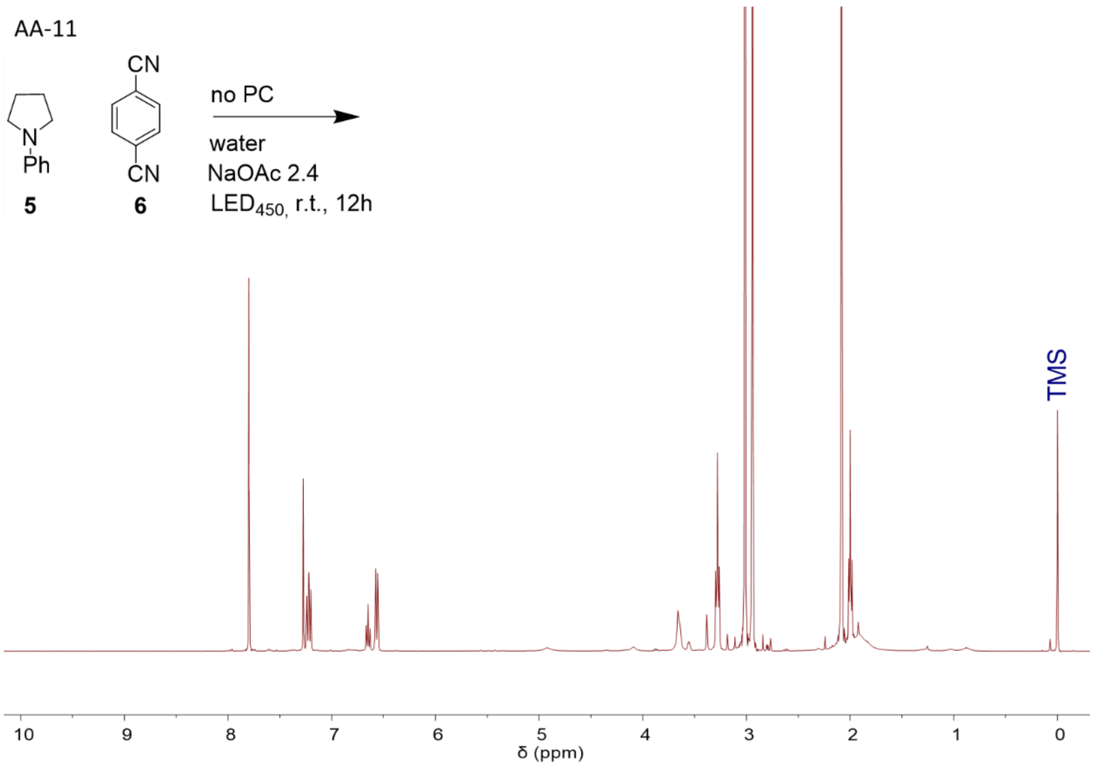

Figure S66. ^{1}H NMR spectrum of the extracted crude from reaction **AA-11.**

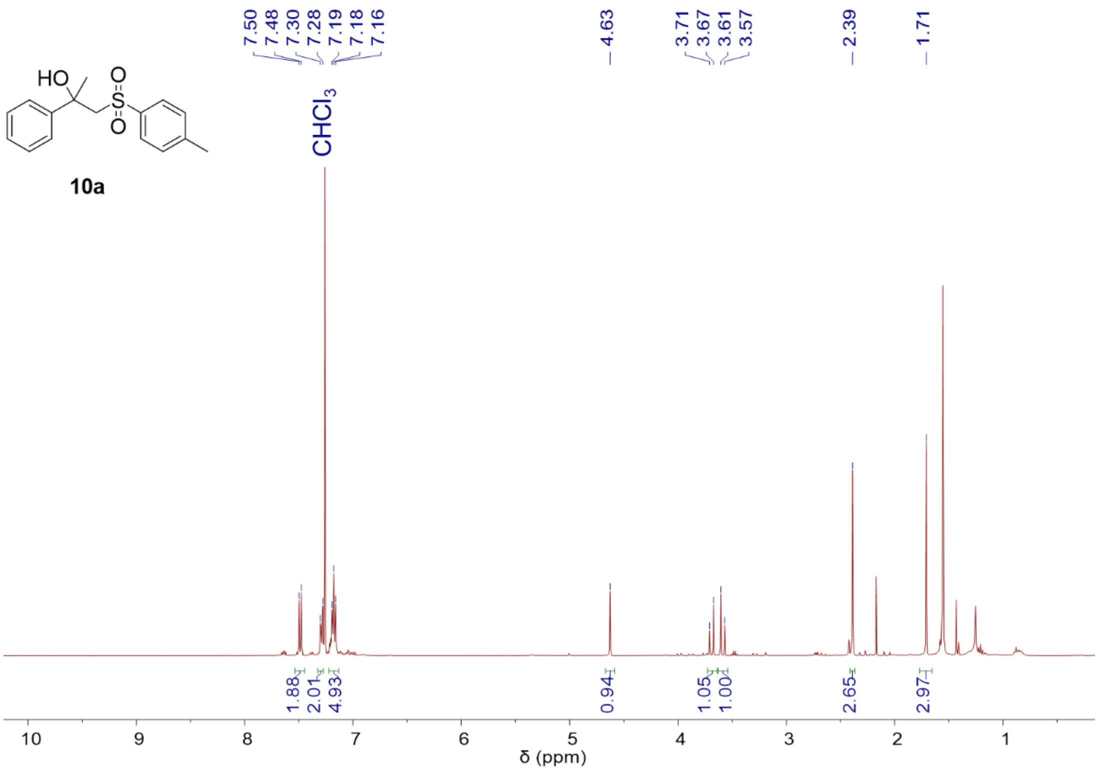

Figure S67. ^{1}H NMR spectrum of **10a**.

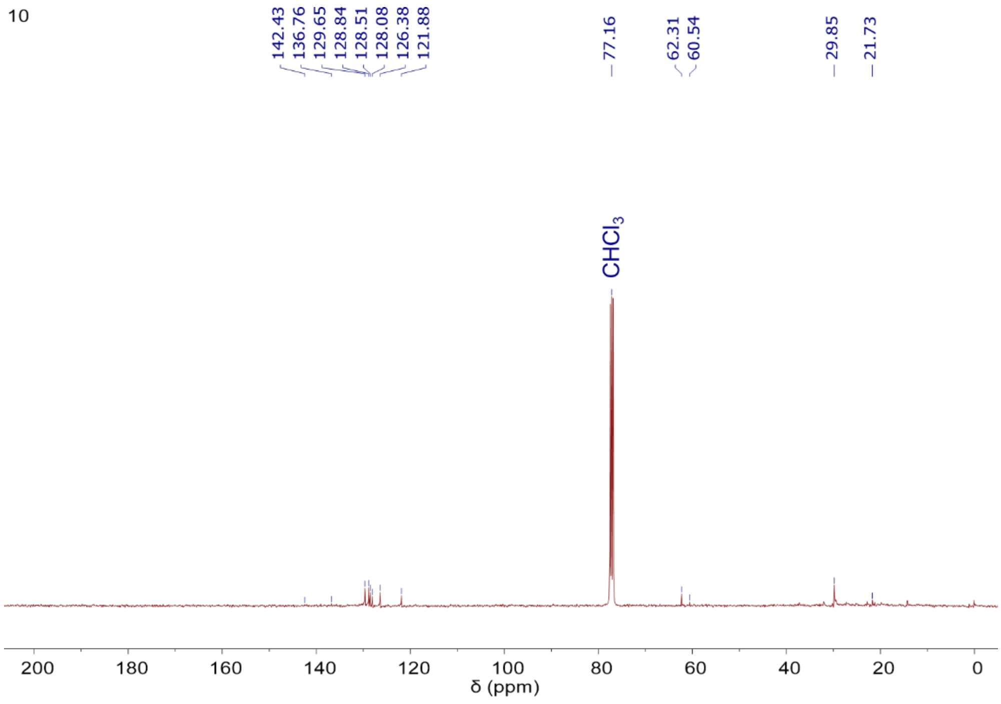

Figure S68. ^{13}C NMR spectrum of **10a**.

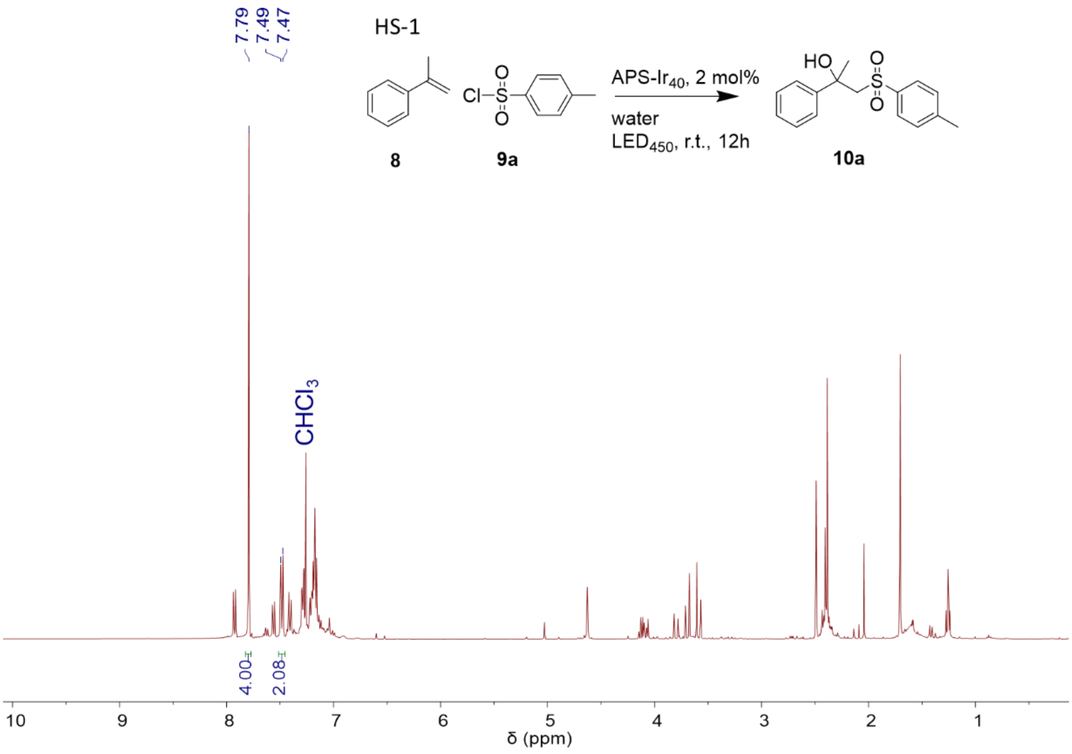

Figure S69. ^{1}H NMR spectrum of the extracted crude from reaction **HS-1.**

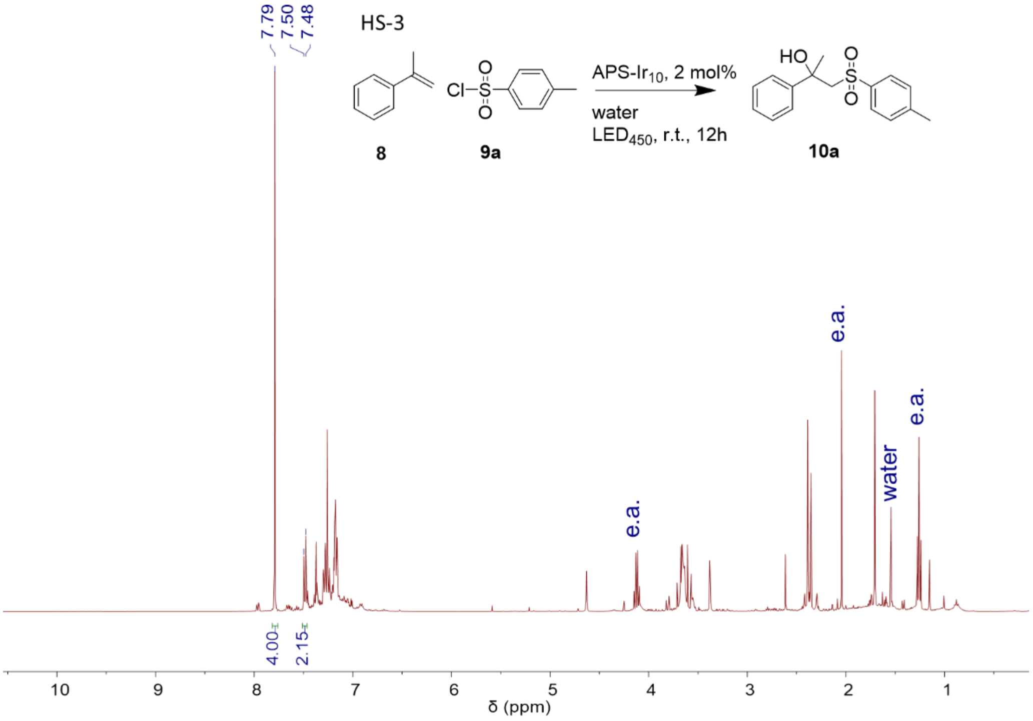

Figure S70. ^{1}H NMR spectrum of the extracted crude from reaction **HS-3** (e.a. = ethyl acetate).

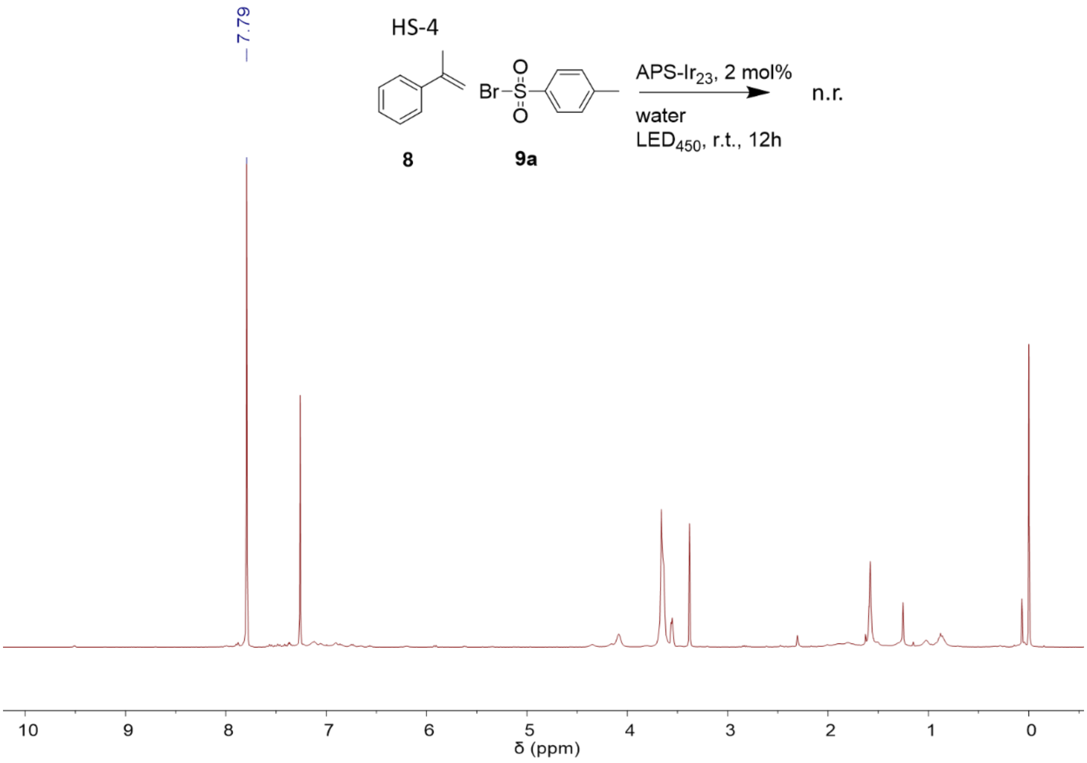

Figure S71. ^{1}H NMR spectrum of the extracted crude from reaction **HS-4.**

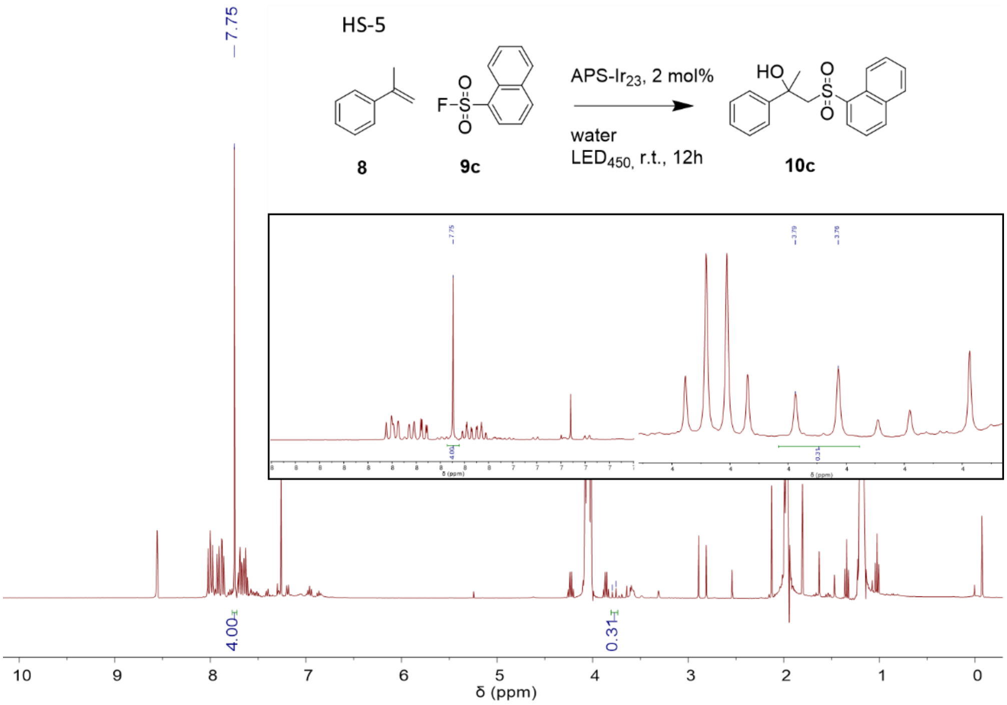

Figure S72. ^{1}H NMR spectrum of the extracted crude from reaction **HS-5.**

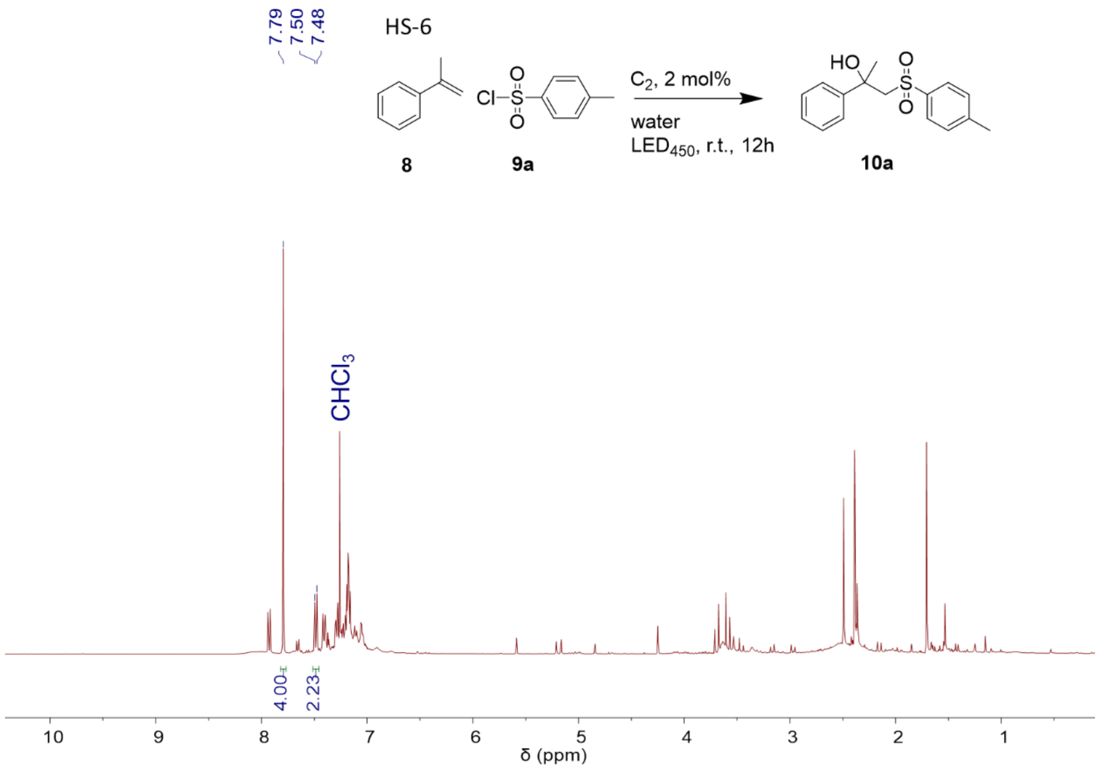

Figure S73. ^{1}H NMR spectrum of the extracted crude from reaction **HS-6.**

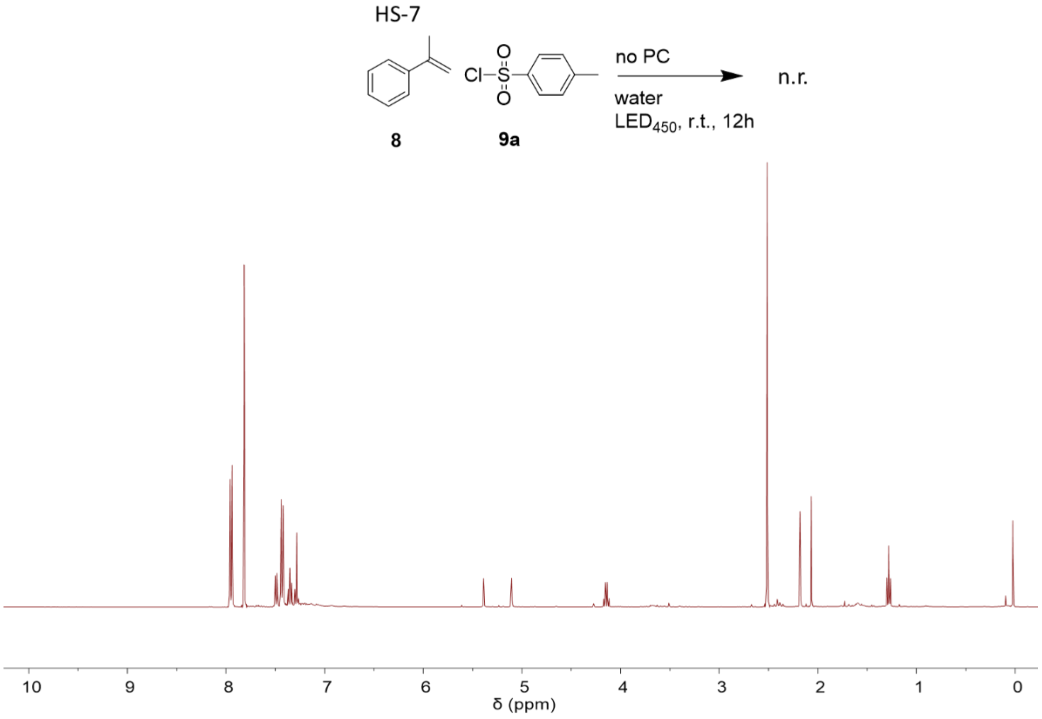

Figure S74. ^{1}H NMR spectrum of the extracted crude from reaction **HS-7.**

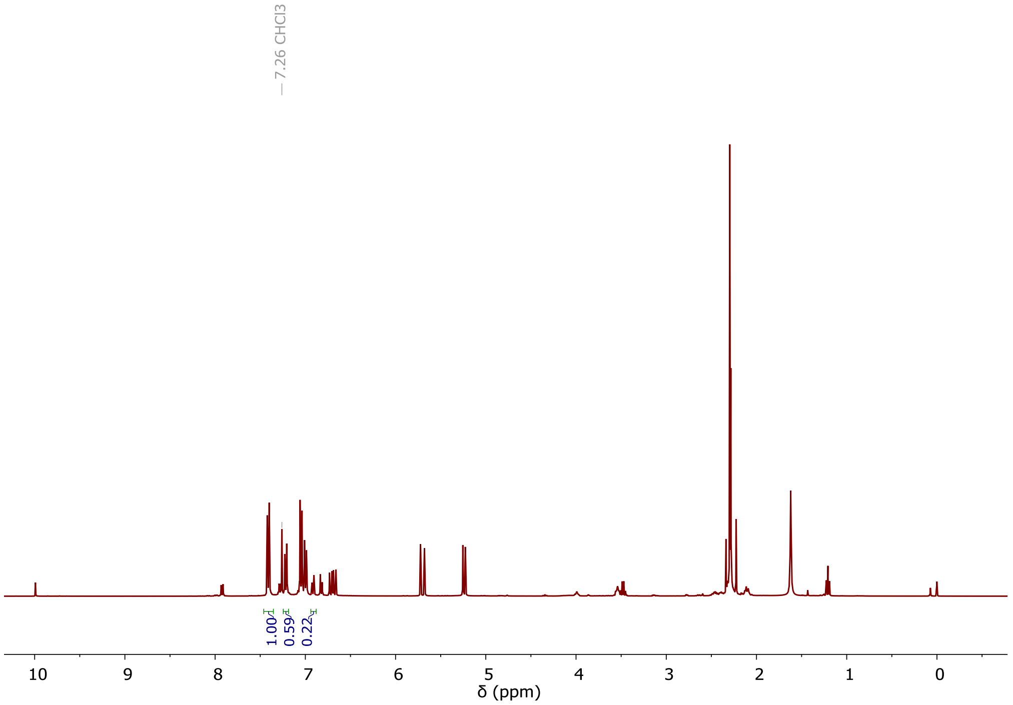

Figure S75. ^{1}H NMR spectrum of the extracted crude of the second catalytic cycle performed by recycled **APS-Ir40** for the "*in water*" [2+2] photocycloaddition of **1a** under title conditions.

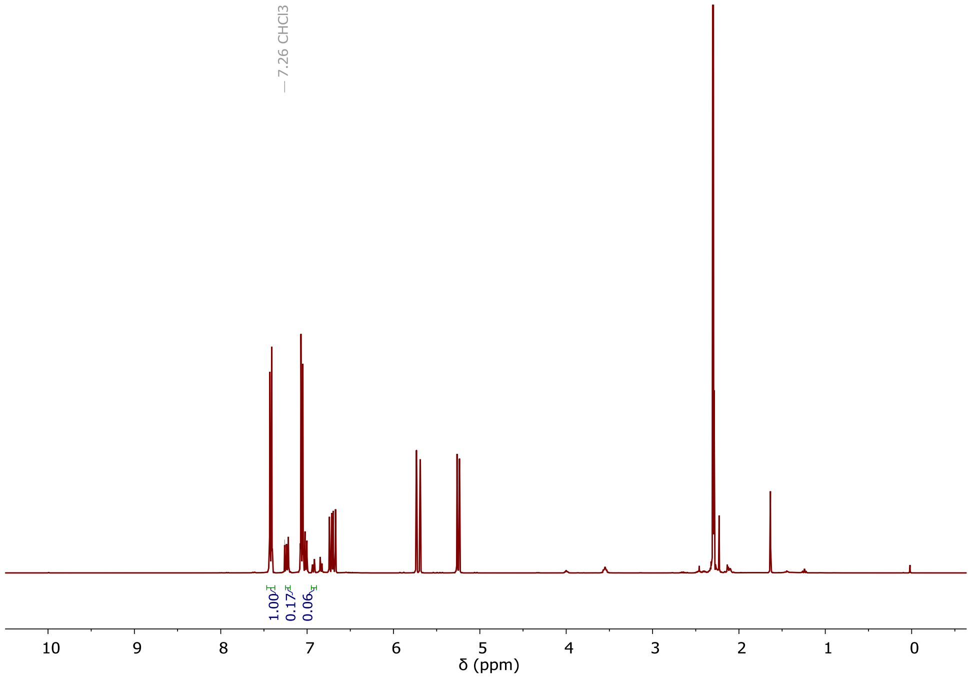

Figure S76. ^{1}H NMR spectrum of the extracted crude of the third catalytic cycle performed by recycled **APS-Ir40** for the "*in water*" [2+2] photocycloaddition of **1a** under title conditions.

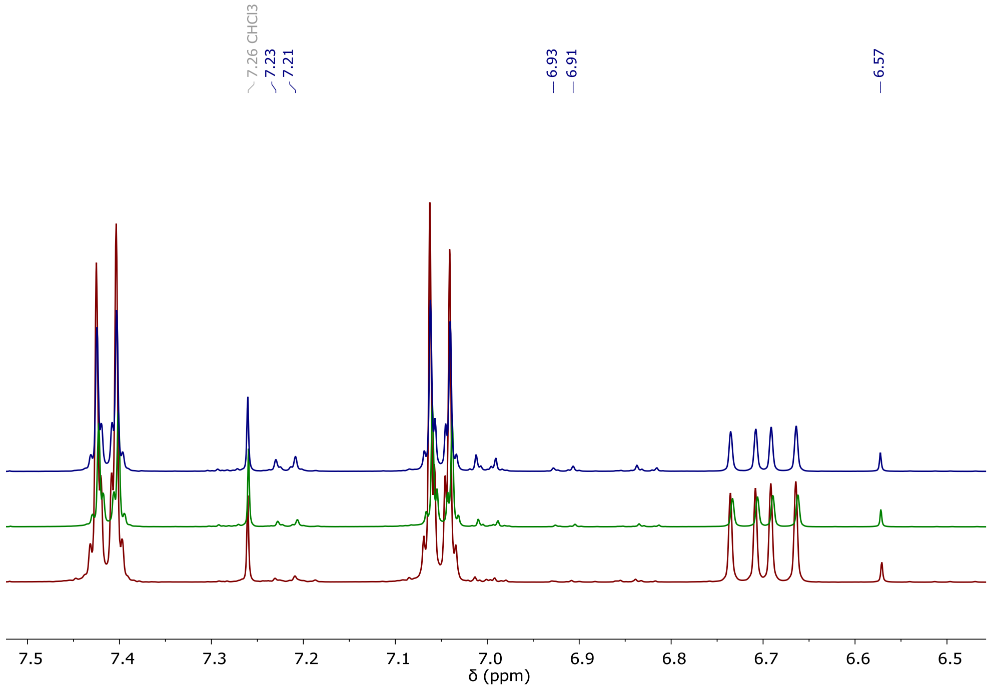

Figure S77. ^{1}H NMR superimposed spectra of the extracted crudes of the **APS-Ir40** -catalyzed "*in water*" [2+2] photocycloaddition of **1a** at [**1a**] = 235 mM. Bottom line, reaction quenched at 600 seconds, middle line, at 1200 seconds and upper line at 1800 seconds.

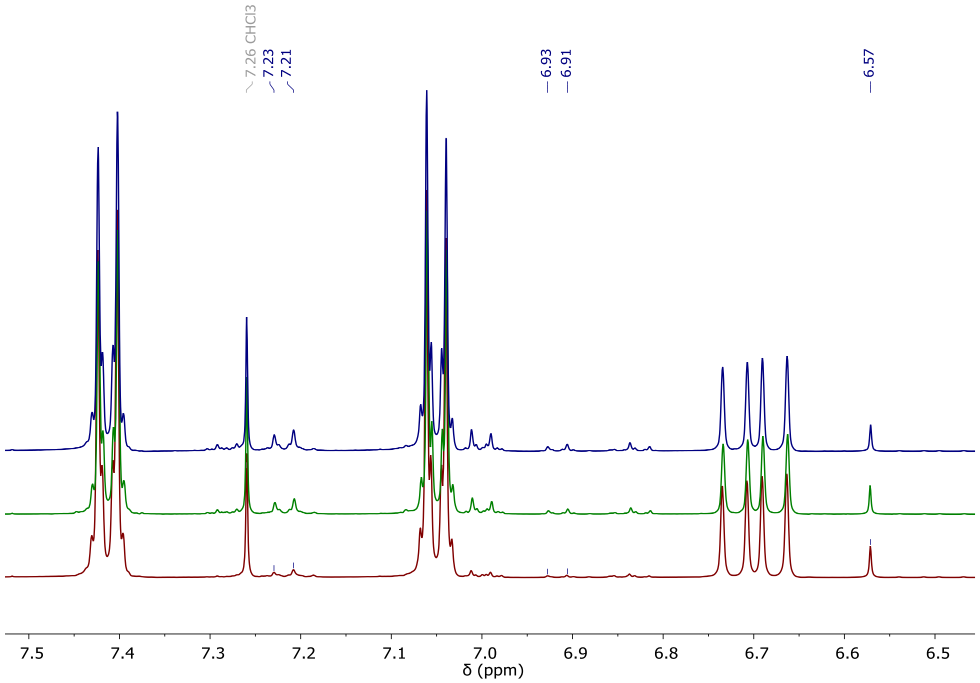

Figure S78. ^{1}H NMR superimposed spectra of the extracted crudes of the **APS-Ir40** -catalyzed "*in water*" [2+2] photocycloaddition of **1a** at [**1a**] = 175 mM. Bottom line, reaction quenched at 600 seconds, middle line, at 1200 seconds and upper line at 1800 seconds.

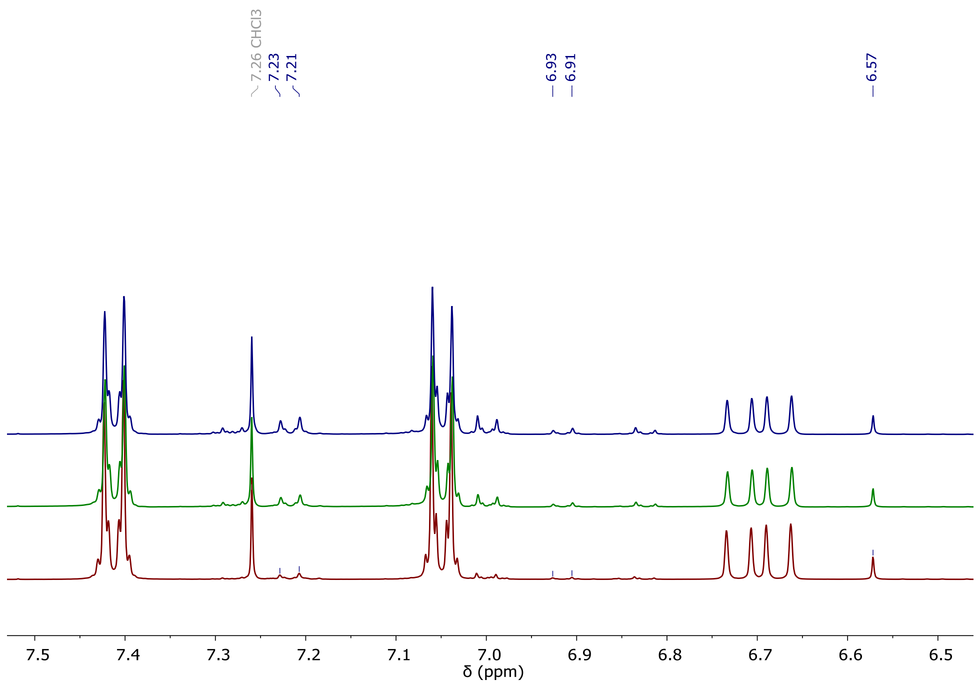

Figure S79. ^{1}H NMR superimposed spectra of the extracted crudes of the **APS-Ir40** -catalyzed "*in water*" [2+2] photocycloaddition of **1a** at [**1a**] = 120 mM. Bottom line, reaction quenched at 600 seconds, middle line, at 1200 seconds and upper line at 1800 seconds.

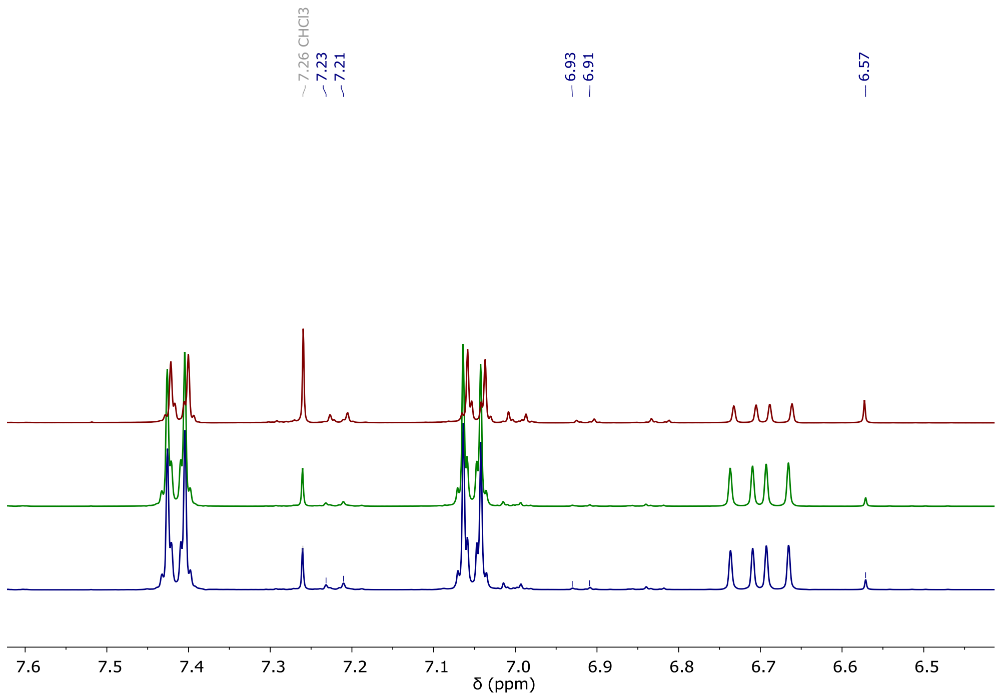

Figure S80. ^{1}H NMR superimposed spectra of the extracted crudes of the **APS-Ir40** -catalyzed "*in water*" [2+2] photocycloaddition of **1a** at [**1a**] = 58 mM. Bottom line, reaction quenched at 600 seconds, middle line, at 1200 seconds and upper line at 1800 seconds.

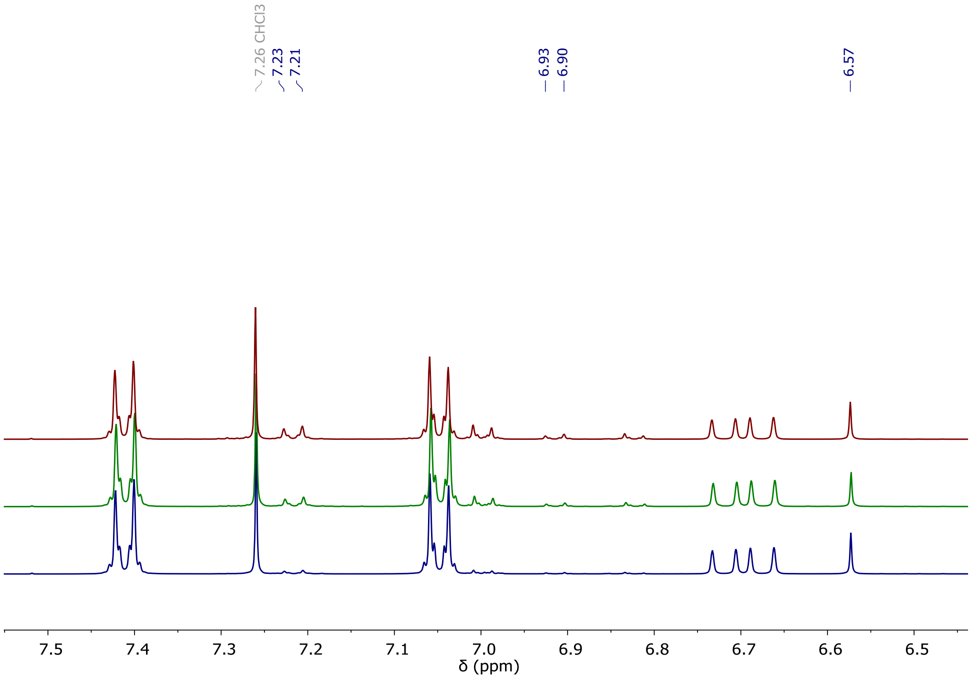

Figure S81. ^{1}H NMR superimposed spectra of the extracted crudes of the **APS-Ir40** -catalyzed "*in water*" [2+2] photocycloaddition of **1a** at [**1a**] = 15 mM. Bottom line, reaction quenched at 600 seconds, middle line, at 1200 seconds and upper line at 1800 seconds.

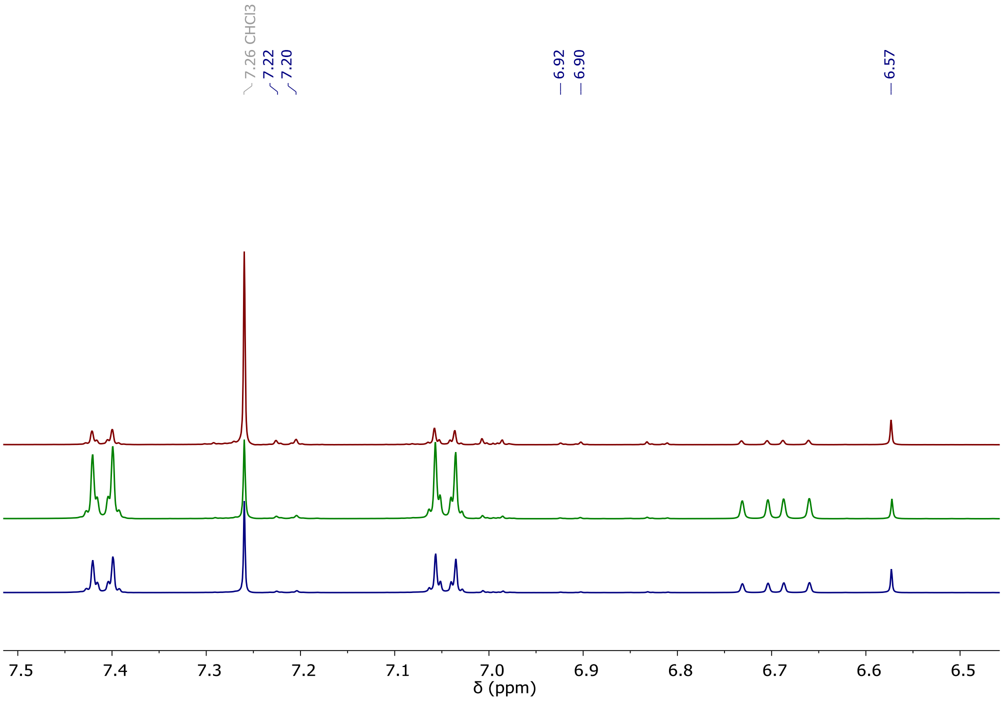

Figure S82. ^{1}H NMR superimposed spectra of the extracted crudes of the **APS-Ir40** -catalyzed "*in water*" [2+2] photocycloaddition of **1a** at [**1a**] = 8 mM. Bottom line, reaction quenched at 600 seconds, middle line, at 1200 seconds and upper line at 1800 seconds.

10. Supplementary references

1. Ulbricht, C.; Becer, C. R.; Winter, A.; Schubert, U. S. RAFT polymerization meets coordination chemistry: synthesis of a polymer-based iridium(III) emitter. *Macromol. Rapid Commun.* **2010**, *31*, 827-833.

2. Beyer, B.; Ulbricht, C.; Winter, A.; Hager, M. D.; Hoogenboom, R.; Herzer, N.; Baumann, S. O.; Kickelbick, G.; Görls, H.; Schubert, U. S. Unexpected metal-mediated oxidation of hydroxymethyl groups to coordinated carboxylate groups by bis-cyclometalated iridium(III) centers. *New J. Chem.* **2010**, *34*, 2622-2633.

3. Golfmann, M.; Glagow, L.; Giakoumidakis, A.; Golz, C., Walker, J. C. L. Organophotocatalytic [2+2]cycloaddition of electron-deficient styrenes. *Chem. Eur. J.* **2023**, *29*, e202202373.

4. Liu, Z.; Zhou, C.; Lei, T.; Nan, X.-L.; Chen, B.; Ting, C.-H.; Wu, L.-Z. Aggregation-enabled intermolecular photo[2+2]cycloaddition of aryl terminal olefins by visible-light catalysis. *CCS Chem.* **2019**, *1*, 582-588.

5. Patent: CN 108623425A, **2018**.

6. Hossain, M.; Shyu, S.-G.; Biphasic copper-catalyzed C-H bond activation of arylalkanes to ketones with tert-butyl hydroperoxide in water at room temperature. *Tetrahedron* **2016**, *72*, 4252-4257.

7. Kumar, R.; Sharma, N.; Sharma, N.; Sharma, A.; Sinha, A. K.; Metal-free activation of H2O2 by synergic effect of ionic liquid and microwave: chemoselective oxidation of benzylic alcohols to carbonyls and unexpected formation of anthraquinone in aqueous condition. *Mol Divers.* **2011**, *15*, 687-695.

8. Majdumar, B.; Bhattacharva, T.; Sarma, T. K. Gold nanoparticle–polydopamine–reduced graphene oxide ternary nanocomposite as an efficient catalyst for selective oxidation of benzylic C(sp3)−H bonds under mild conditions. *ChemCatChem* **2016**, *8*, 1825-1835.

9. McNally, A.; Prier, C. K.; MacMillan, D. W. C.; Discovery of an α-amino C-H arylation reaction using the strategy of accelerated serendipity. *Science* **2011**, *334*, 1114-1117.

10. Stevenson, B. G.; Spielvogel, E. H.; Locaiani, E. A.; Wembua, V. M.; Nakhamiyayev, R. V.; Swierk, J. R.; Mechanistic investigations of an α-aminoarylation photoredox reaction. *J. Am. Chem. Soc.* **2021**, *143*, 8878-8885.

11. Quintavalla, A.; Carboni, D.; Sepe, C.; Mummolo, L.; Zaccheroni, N.; Lombardo, M.; Towards a more sustainable photocatalyzed α-arylation of amines: green solvents, catalyst recycling and loading. *Adv. Syn. Catal.* **2023**, *365*, 252-262.

12. Taniguchi, T.; Idotaa, A.; Ishibashia, H.; Iron-catalyzed sulfonyl radical formations from sulfonylhydrazides and oxidative addition to alkenes. *Org. Biomol. Chem.* **2011**, *9*, 3151-3153.

13. Srinivasan, B. A guide to the Michaelis-Menten equation: steady state and beyond. *FEBS J.* **2022**, *289*, 6086-6098.
